# Histology and affinity of anaspids, and the early evolution of the vertebrate dermal skeleton

**DOI:** 10.1098/rspb.2015.2917

**Published:** 2016-03-16

**Authors:** Joseph N. Keating, Philip C. J. Donoghue

**Affiliations:** 1 School of Earth Sciences, University of Bristol, Life Sciences Building, Tyndall Avenue, Bristol BS8 1TQ, UK; 2 Department of Earth Sciences, Natural History Museum, Cromwell Road, South Kensington, London SW7 5BD, UK

**Keywords:** histology, anaspid, gnathostome, skeleton, dermal, odontode

## Abstract

The assembly of the gnathostome bodyplan constitutes a formative episode in vertebrate evolutionary history, an interval in which the mineralized skeleton and its canonical suite of cell and tissue types originated. Fossil jawless fishes, assigned to the gnathostome stem-lineage, provide an unparalleled insight into the origin and evolution of the skeleton, hindered only by uncertainty over the phylogenetic position and evolutionary significance of key clades. Chief among these are the jawless anaspids, whose skeletal composition, a rich source of phylogenetic information, is poorly characterized. Here we survey the histology of representatives spanning anaspid diversity and infer their generalized skeletal architecture. The anaspid dermal skeleton is composed of odontodes comprising spheritic dentine and enameloid, overlying a basal layer of acellular parallel fibre bone containing an extensive shallow canal network. A recoded and revised phylogenetic analysis using equal and implied weights parsimony resolves anaspids as monophyletic, nested among stem-gnathostomes. Our results suggest the anaspid dermal skeleton is a degenerate derivative of a histologically more complex ancestral vertebrate skeleton, rather than reflecting primitive simplicity. Hypotheses that anaspids are ancestral skeletonizing lampreys, or a derived lineage of jawless vertebrates with paired fins, are rejected.

## 1. Introduction

**Figure 1.**
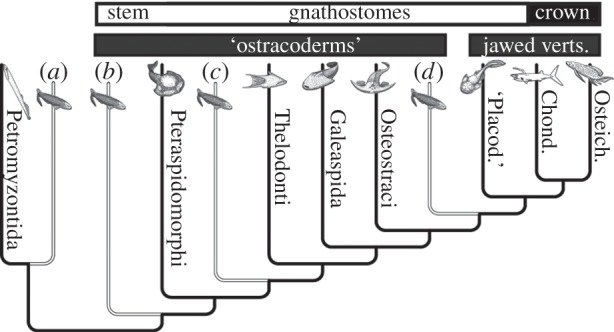
Vertebrate phylogeny showing conflicting hypotheses of anaspid affinity. (*a*) Anaspids are stem-lampreys [3,4]; (*b*) anaspids are the most deeply branching ostracoderms [5–10]; (*c*) anaspids are nested within ostracoderms [3,4,11–13]; (*d*) anaspids are the sister group of jawed vertebrates [14].

Almost all living vertebrates are jawed vertebrates (crown gnathostomes), a clade characterized not only by the possession of jaws, but by a suite of additional features not seen in the living jawless vertebrates, the hagfishes and lampreys. These include a mineralized skeleton, vertebrae, paired nostrils, a stomach, a complex inner ear, endoskeletal sclera, sclerotic ossicles, and paired pectoral and pelvic appendages. This phylogenetic gulf, between living jawless and jawed vertebrates, is bridged by a diverse array of extinct fishes that comprise the gnathostome stem, and are known collectively as the ‘ostracoderms’ because they possess extensively developed dermal armour. These extinct clades are related by degree to crown-gnathostomes, as evidenced by hierarchically nested sets of shared characters, documenting the sequence and tempo of gnathostome bodyplan assembly [1,2]. While the phylogenetic positions and, consequently, the evolutionary significance of most ostracoderm clades are well-resolved, a small number have proven resistant to phylogenetic resolution. Chief among these are the anaspids (figure 1), which have been alternately considered stem-lampreys [3,4], the earliest branching lineage of skeletonizing vertebrates [5–10], nested among the ostracoderms [3,4,11–13], or as the sister group of jawed vertebrates [14]. Only one, if any of these phylogenetic hypotheses can be correct and, in large part, this phylogenetic uncertainty stems from a paucity of knowledge and understanding of the anaspid skeleton—a key source of phylogenetic information for early vertebrates. While the gross anatomy of anaspids has been well characterized, there have been few attempts to resolve the nature of anaspid skeletal tissues and these studies have been stymied by the small size of anaspid skeletal elements and the enigmatic nature of their tissues.

Thus, in an attempt to reduce phylogenetic uncertainty and resolve the evolutionary significance of anaspids, we characterized the nature of the mineralized skeleton in species representative of the breadth of anaspid diversity. From this we infer the plesiomorphic histology of the anaspid dermal skeleton, revise and expand histological characters in existing phylogenetic datasets, and build upon the ensuing phylogenetic hypotheses to realize the evolutionary significance of anaspids in understanding the assembly of the gnathostome bodyplan.

## 2. Historical review

The first description of anaspid scale morphology and histology was provided by Christian Heinrich Pander [15] who observed that the scales, including the visceral ribs and superficial ornament, were entirely formed of a compact lamellar tissue. In some specimens, he noted that lamellae were penetrated by fine calibre spaces. While Pander regarded these peculiar fossils as vertebrates, Rohon [16], could not reconcile the homogeneous lamellar tissue with any known vertebrate skeletal histology and so he rejected vertebrate affinity. A number of early interpretations were confounded by the homogeneous nature of the anaspid mineralized skeleton. For example, Traquair [17] commented ‘the structure of the substance forming dermal scales of *Birkenia* shows neither the bone-lacunae of the Osteostraci nor the dentine tubules of the Heterostraci, but so far as I have been able to examine them microscopically, nothing is seen but a homogeneous, or slightly fibrillated mass, though this may possibly be the result of faulty preservation’. Similarly, Kiaer [18] remarked ‘There is of course a possibility that the dermal skeleton of the Anaspida was extremely simply constructed without bone cells, as is the case with the chief mass of the dermal skeleton of Pteraspidae, but it is doubtless more probable that the original structure has disappeared’. The microstructure of the anaspid dermal skeleton was most thoroughly described by Gross [19–21] who observed that anaspid scales are constructed from a homogeneous tissue of compact concentric lamellae, pervaded by an extremely fine radial fabric of thread-like spaces which he compared to the unmineralized spaces present in the middle and basal layer of the dermal skeleton in psammosteid heterostracans, similarly interpreting these spaces as having housed collagen fibres [19,21]. Anterior of the medial visceral rib, Gross [19] noted that the radial fabric is obscured by an obliquely oriented and less regular fabric of coarse spaces, which he interpreted as Sharpey's fibres.

Although the superficial layer of the anaspid dermal skeleton is composed of discrete tubercles, Gross [19,21] found no evidence of dentine tubules, pulp cavities or an enameloid capping layer. He considered these tubercles as histologically indistinguishable from the underlying lamellar tissue, observing in some particularly well-preserved specimens that the tubercle tissue is also pervaded by fine radial thread-like spaces [19]. He initially described the microstructure of the anaspid dermal skeleton as ‘acellular fibreous bone’ and suggested that it perhaps grew in a manner comparable with the acellular bone of teleosts [21]. However, in later publications he identified the lamellar tissue as ‘aspidin’ [19,20], comparing it directly to the tissue comprising the middle and basal layer in the heterostracan dermal skeleton.

Gross [19] interpreted the concentric lamellar construction of the anaspid scales as evidence of discrete growth intervals. Based on this interpretation, he reasoned the scales exhibit allometric growth. *Rhyncholepis* was shown to possess an extensive branching vascular layer, underlying the superficial sculpture. The canals of the vascular layer are enveloped by centripetal lamellar tissue, which overprints the concentric lamellae of the scale [19]. Gross interpreted the vascular canals as primary osteons, suggesting the three-layered skeleton of the material from Saaremaa and Beyrichienkalk developed via resorptive expansion of the vascular layer.

Anaspid histology has been considered most recently by Blom *et al.* [22–24]. These studies have greatly improved understanding of the diversity of anaspid dermal skeleton architectures, which in turn has allowed for better classification of the taxa known exclusively from scales. Furthermore, integration of these data into phylogenetic analyses has shed light upon the relationships between anaspid taxa. However, these studies provide insight only into the gross histological structure of the scales, insufficient to test among hypotheses of tissue homology. Thus, understanding of anaspid tissue microstructure and homology has not progressed beyond the studies of Gross [19,21]. To this end, we set out to re-characterize the histology of the dermal skeleton in taxa representative of the major lineages of anaspid phylogeny and infer the plesiomorphic nature of anaspid dermal skeletal histology. Finally, we use these data to revise recent phylogenetic datasets in order to resolve the phylogenetic affinity and reveal the evolutionary significance of anaspids.

## 3. Material and methods

### (a) Material

We surveyed the histology of eight anaspid taxa spanning the diversity of the clade [9]. Material assigned to *Birkenia robusta* (NHMUK PV P73701-3), *Rytidolepis quenstedtii*, *Rhyncholepis butriangula* (NHMUK PV P73704-7), *Rhyncholepis parvula* and *Vesikulepis funiforma* (NHMUK PV P73708-10) is from the Early Silurian (Wenlock) age Vesiku beds of Saaremaa, Estonia [22]. Our description focuses on *Birkenia robusta*, *Vesikulepis funiforma* and *Rhyncholepis butriangula*, as these taxa encompass anaspid phylogenetic disparity; only deviations from their gestalt are discussed. Material assigned to *Pterygolepis nitida* is from Gothemshammar, Gotland, and is also Early Silurian (Wenlock) in age [22]. Material assigned to *Manbrookia asperella* and *Septentrionia lancifera* is from the Late Silurian (Přídolí) of Man Brook, near Trimpley, Worcestershire, UK [22]. Data were collected using synchrotron radiation X-ray tomographic microscopy (srXTM), scanning electron microscopy (SEM) and light microscopy (LM). Figured specimens are stored at the Natural History Museum, London, UK (NHM UK).

### (b) Synchrotron radiation X-ray tomographic microscopy

The srXTM experiments were conducted at the TOMCAT beamline of the Swiss Light Source, Paul-Scherrer Institut, Villigen, Switzerland. Measurements were taken using 20× and 40× objective lenses, 15–24 keV and 100–200 ms exposures. For each dataset, 1501 equiangular projections were acquired over 180 degrees. These were post-processed and rearranged into flat- and dark-field-corrected sinograms. Reconstruction was performed on a 60-core Linux PC farm, using a highly optimized routine based on the Fourier transform method and a regridding procedure, resulting in volumetric data with voxel dimensions of 0.325 µm (20× objective) and 0.1625 µm (40× objective). The data were analysed in Aviso 8.0. Tomographic sections were produced using the orthoslice module. Virtual thin sections were produced using the volume rendering module. The data were cropped producing thin sections 10–100 slices thick. Three-dimensional virtual models of scales were produced using the isosurface module. Growth stages were visualized using the segmentation module.

### (c) Scanning electron microscopy and light microscopy

Specimens were embedded in Struers EpoFix^®^ resin and cured for 24 h. Sections were cut with a Buehler IsoMet^®^ low speed saw. Cut surfaces were impregnated using low viscosity Buehler EPO-THIN^®^ resin. The impregnated surface was ground manually using P600 to P2500 grit sizes. EPO-Thin^®^ resin was used to attach cut specimens to glass slides for light microscopy. Specimens were polished manually using Buehler MetaDi^®^ 6 µm and 1 µm diamond paste. Polished samples were then etched with 0.5% orthophosphoric acid for 10  min. Samples were carbon coated using an Emitech K450 carbon coater. The specimens were imaged using a Hitachi S-3500N SEM. This work was undertaken at the University of Bristol School of the Earth Science's Electron Microbeam Facility. Thin sections were examined using a Leica M205C microscope with a 2× Plan Apochromat lens and imaged with a Leica DFC425C digital camera.

### (d) Phylogenetic analyses

We combined and updated the most recent analyses of lower vertebrate interrelationships [5,8,9,11]. For a comprehensive list of revisions, see the electronic supplementary material. The final matrix consisted of 120 characters and 21 taxa (including a single outgroup). Our analyses assume a monophyletic Cyclostomata (hagfish plus lampreys) even though it is not the most parsimonious hypothesis of hagfish, lamprey and crown-gnathostome relationships recovered from unconstrained phylogenetic analysis of these morphological data. We did this because Cyclostomata is supported unequivocally by a wealth of molecular data and because morphological data cannot discriminate statistically between cyclostome monophyly and paraphyly [25]. The matrix was otherwise subjected to parsimony analysis using TNT v.1.1 and statistical tests (Templeton test [26], Kishino–Hasegawa test [27]; approximate Shimodaira–Hasegawa test [28]) of competing phylogenetic positions for anaspids were conducted in PAUP 4.0b 10. Consensus networks were generated using SplitsTree v. 4.13.1.

## 4. Results

### (a) Histological analyses

#### (i) Birkenia robusta

**Figure 2.**
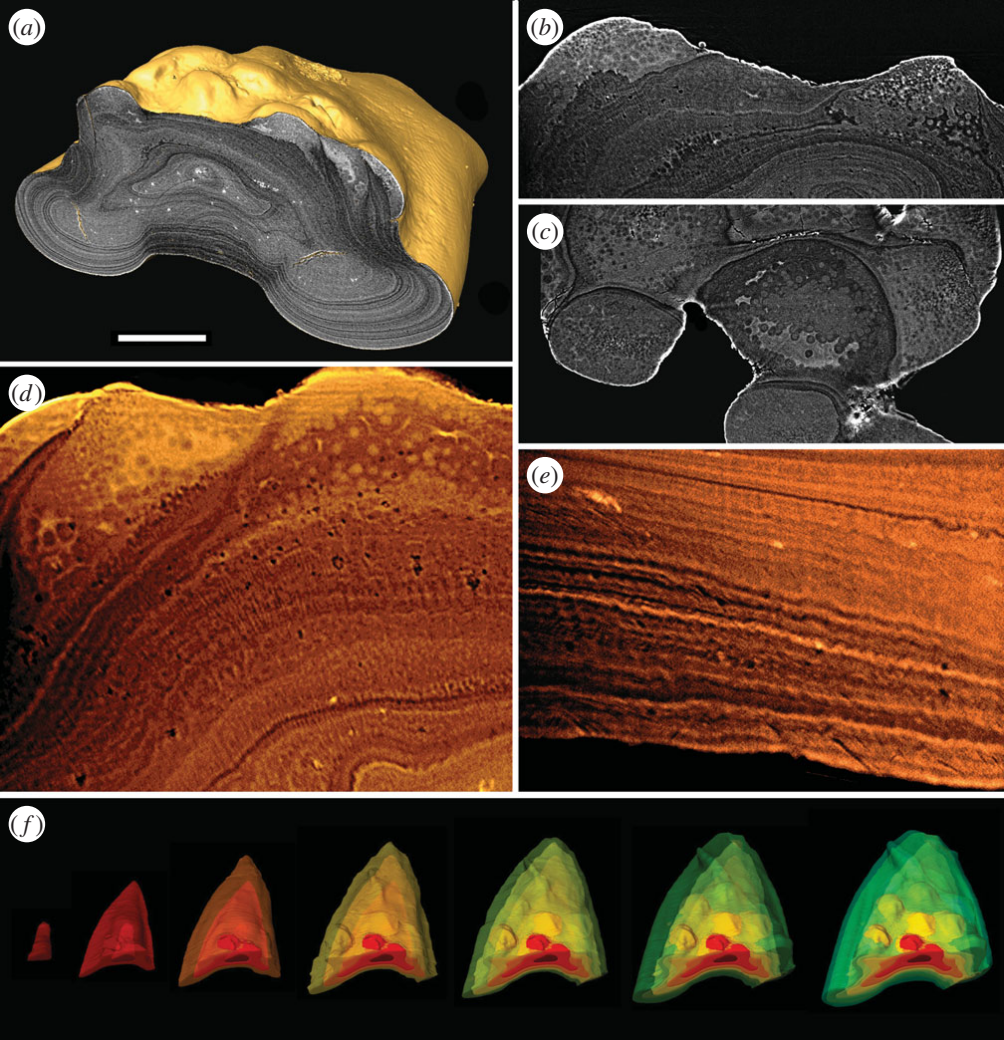
srXTM histological sections and models of the dermal skeleton of *Birkenia robusta* (specimen lost). (*a*) Isosurface model with transverse section showing highly attenuating superficial tubercles overlying a basal layer composed of compact lamellae; transverse (*b*) and horizontal (*c*) sections through the spheritic superficial tubercles; (*d*) volume rendered virtual thin section showing the superficial tubercles and underlying acellular bone with pervading fabric of linear thread-like spaces; (*e*) volume rendered virtual thin section of the basal compact lamellae of the visceral rib; (*f*) segmented growth series of an a body scale. Scale bar equals 172 µm in (*a*), 42 µm in (*b*), 92 µm in (*c*), 36 µm in (*d*), 36 µm in (*e*) and 514 µm in (*f*).

Scales are constructed from concentric bands of lamellae, interpreted as growth increments. Each scale consists of two discrete layers: a superficial layer of spheteric tubercles and a basal laminated layer (figure 2*a*). *Superficial layer*: measures around 10–15% of the total scale thickness (approx. 25–35 µm). Scales ornamented with large obtuse tubercles, approximately equally spaced over the surface. Overlapped area is smooth and devoid of tubercles. The tubercles exhibit a heterogeneous spheritic microstructure (figure 2*b*–*d*). The core of each tubercle consists of a compact tissue composed of tiny mineralized spheres (approx. 1–4 µm in diameter), about which a second fabric of coarse spheres (each measuring 5–10 µm in diameter) is developed (figure 2*c*). The coarse spheres exhibit a concentric microstructure, indicating mineralization occurred about a nucleating centre. Spaces between the coarse spheres are infilled by a conspicuous highly attenuating mineral. This is unlikely a product of digenetic alteration, as the infilling material intergrades imperceptibly with the underlying lamellar tissue. In a similar fashion, the spheritic tissue at the base of the tubercle intergrades with the underlying lamellar tissue (figure 2*b*,*d*). *Basal layer*: comprises around 85–90% of the total thickness of the dermal skeleton (approx. 230 µm). It is composed a concentric lamellar tissue, which is avascular. The core of each scale contains an intrinsic linear fabric of elongate spaces infilled with pyrite (figure 2*a*). The spaces measure approximately 30–50 µm in length and are orientated parallel to the longitudinal axis of the scale. These are consistent with voids left by intrinsic collagen fibre-bundles. The outer most concentric lamellae, which form the lateral margins and base of each scale, are highly compact forming sharp regular bands differentiated by differences in X-ray attenuation (figure 2*e*). A radial fabric of tightly packed linear spaces pervades these lamellae. The spaces are aligned orthogonal to, and appear to warp the boundaries of the contiguous lamellae through which they pervade. The spaces each measure less than 1 µm in diameter. In the lamellar tissue underlying the tubercles, the radial fabric is particularly well developed (figure 2*d*). The spaces terminate abruptly at the tubercles and do not pervade the spheritic tissue. Between the visceral ribs, the lamellae are less regular and the radial linear fabric cannot be discerned.

The concentric lamellae of the basal layer are organized in discrete bands that reflect incremental growth of the scale. They reveal a pattern of appositional growth in which the scale is enveloped by each subsequent concentric growth increment (figure 2*a*). The scale shows allometric growth. The visceral ribs, which are poorly developed in the earliest growth stage, are incrementally thickened through ontogeny. The spheritic tubercles are not typically enveloped by successive growth increments; rather apposition of new tissue occurs on the flanks of the pre-existing tubercle, leaving the crown exposed. New tubercles are added marginally with each successive growth increment (figure 2*f*).

#### (ii) Vesikulepis funiforma

**Figure 3.**
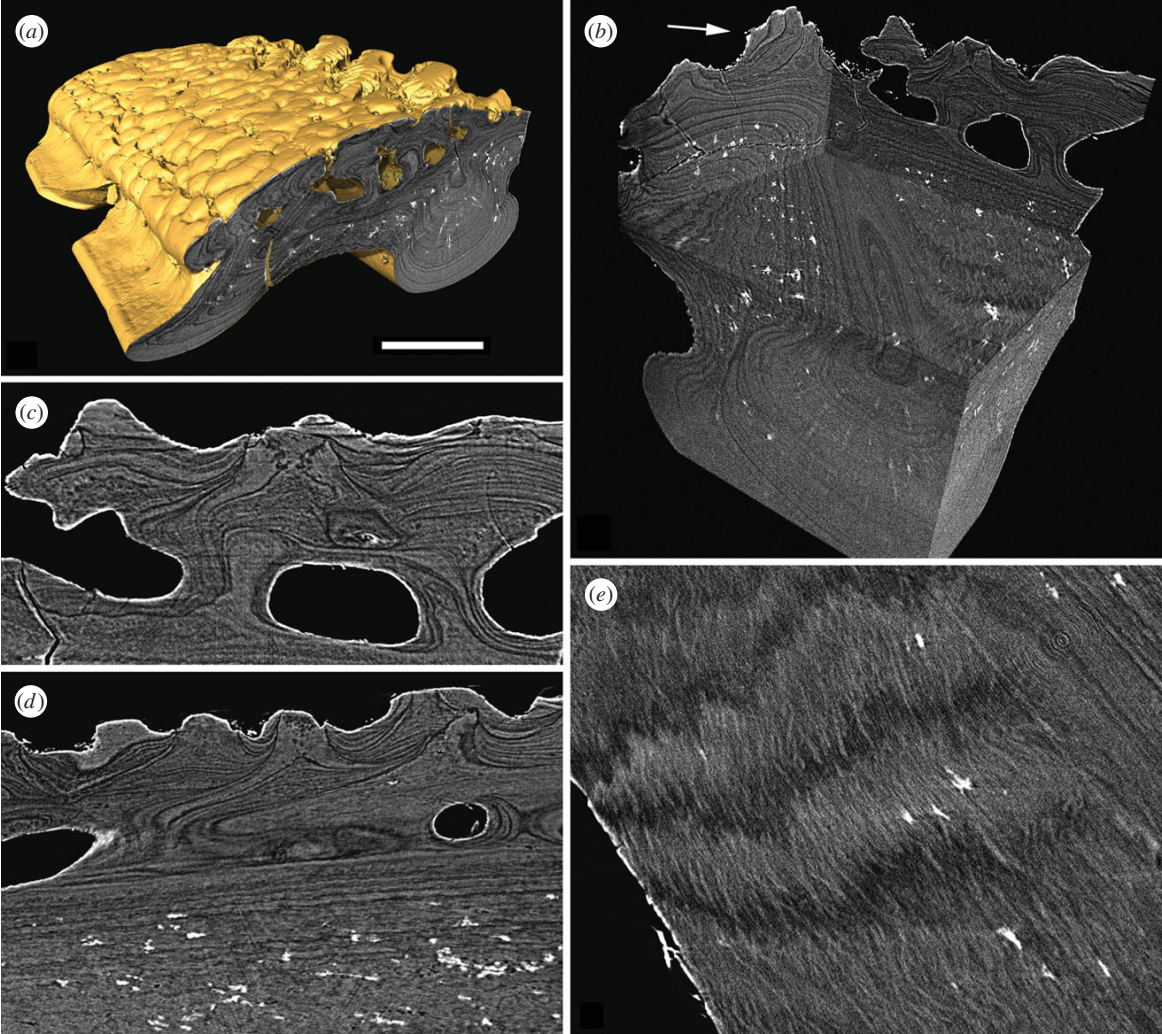
srXTM histological sections of the dermal skeleton of *Vesikulepis funiforma* (NHM PV P73708). (*a*) Transverse section through a body scale; (*b*) block model constructed from histological slices showing three-dimensional microstructure of the dermal skeleton. The arrow points to a posterior tubercle ridge constructed from discrete, wedge-shaped tubercles; (*c*,*d*) transverse sections through the superficial layer and pore canal network of the overlapped area; (*e*) horizontal section through lamellae of the basal layer showing parallel intrinsic collagen bundles between contiguous lamellae. Scale bar equals 140 µm in (*a*), 103 µm in (*b*), 44 µm in (*c*), 60 µm in (*d*) and 39 µm in (*e*).

Scales are constructed from concentric bands of lamellae, interpreted as discrete growth increments. Each scale is two-layered, consisting of a superficial layer of spheritic tubercles supplied by a shallow series of vascular canals and a laminated basal layer (figure 3*a*,*b*). *Superficial layer*: comprises around 10% of the total dermal skeletal thickness (measuring approx. 60–80 µm). Scales exhibit two distinct areas of ornament on the external surface. The tubercles of the overlapped area are small, irregular and tightly packed while the posterior of the scale is ornamented with finely segmented ridges, which run obliquely to the long axis of the scale. Both sets of tubercles exhibit heterogeneous spheritic microstructure similar to the condition in *Birkenia* (figure 3*c*,*d*). Each tubercle comprises a core of minute mineralized spheres (each measuring less than 1 µm in diameter) enclosed within a fabric of coarser spheres (each approx. 2 µm in diameter). As in *Birkenia*, the space between the spheres is infilled with a highly attenuating material. The spheretic tubercles intergrade imperceptibly with the underlying lamellae. The overlapped area shows evidence of superpositional growth, with late generation tubercles overriding early generation tubercles (figure 3*d*). The ridges ornamenting the posterior surface of the scale comprise multiple overlapping wedge-shaped tubercles, giving them a finely segmented appearance (figure 3*b*). Between the tubercles and ridges, the external surface is perforated by evenly distributed pores, which open into a series of shallow longitudinal vascular canals, measuring approximately 20 µm in diameter. The mineralized walls that define the canals are formed of acellular and afibreous lamellar tissue. The lamellae appear to wrap around the canals, indicating vasculogenesis preceded or occurred simultaneously with skeletogenesis (figure 3*c*,*d*). The lamellar tissue, underling the tubercles, is pervaded by a fine fabric of orthogonal linear thread-like spaces, each measuring less than 1 µm in diameter (figure 3*c*). These spaces terminate abruptly at the boundary between the spheritic tissue and the underlying lamellae. *Basal layer*: measures approximately 700 µm comprising around 90% of the total skeletal thickness. The basal layer is composed of lamellar tissue, circumscribing the base of the scale and the visceral ribs. Each lamella contains an intrinsic fabric of collagen fibre bundles, aligned obliquely to the lamellar boundaries and parallel to the intrinsic fibre bundles of contiguous lamellae (figure 3*e*). Under crossed polarized light, contiguous lamellae show corresponding anisotropic extinction, consistent with an interpretation as parallel fibred bone [29]. Within the visceral ribs, the tissue is compact, extremely regular and perforated by a similarly regular fabric of long radiating spaces infilled by pyrite. On either side, adjacent to the visceral ribs, the lamellar tissue is pervaded by a meshwork of coarse unmineralized spaces, infilled with pyrite (figure 3*b*). These spaces have previously been identified as Sharpey's fibres [19], However, while these spaces are consistent with extrinsic fibres of some description, the fabric is poorly organized and the spaces do not end abruptly at lamellar boundaries or perforate the base of the scale, as is the case in Sharpey's fibres, which are essentially anchoring tethers that bind the base of the dermal skeleton to the underlying dermis.

#### (iii) Rhyncholepis butriangula

**Figure 4.**
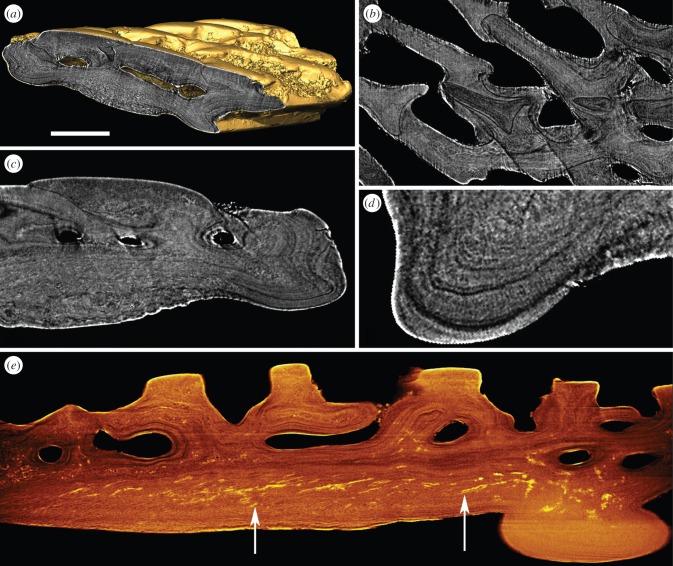
srXTM histological sections of the dermal skeleton of *Rhyncholepis butriangula* (NHM PV P73705). (*a*) Transverse section through an isosurface model; (*b*) horizontal section through the spheritic tubercles of the superficial layer; (*c*) transverse section showing the spheritic superficial layer; (*d*) transverse section showing the compact basal layer with pervading fabric of linear thread-like spaces; (*e*) volume rendered virtual thin section. Arrows point to the coarse extrinsic fabric of fibre spaces infilled with pyrite. Scale bar equals 126 µm in (*a*), 93 µm in (*b*), 70 µm in (*c*), 24 µm in (*d*) and 69 µm in (*e*).

Scales are constructed from concentric bands of lamellae, interpreted as discrete growth increments. The scales are formed of two distinct layers: a superficial layer of spheritic tubercles supplied by a series of shallow vascular canals and a laminated basal layer (figure 4*a*). *Superficial layer*: comprises around 10–20% of the total scale thickness (measuring approx. 30–40 µm). The scale ornament consists of parallel rows of posteriorly pointing elongate triangle-shaped tubercles. These tubercles are added superpositionally from posterior to anterior, so that the apexes of the most recent tubercle generation overrides the base of the previous tubercle generation. The tubercles are composed of spheritic tissue comparable with that of *Birkenia* and *Vesikulepis*. The core of the tubercles is composed of densely compacted mineralized spheres, each measuring around 1–2 µm in diameter. The spheritic texture of the outer layer of the tubercles is less clearly defined, and it appears to pass imperceptibly into the underlying lamellar tissue (figure 4*b*,*c*). Between the tubercle rows are numerous pores, which open into the vascular canals via small ascending canals. The vasculature consists of a longitudinal series of canals measuring 25–30 µm in diameter. The concentric bands of lamellae, interpreted as growth increments, appear to wrap around the vascular spaces, indicating that vasculogenesis preceded or coincided with skeletogenesis (figure 4*c*). Some of the vascular spaces are enveloped by a centripetal lamellar tissue, suggesting the canals developed in a similar fashion to osteons. *Basal layer*: 170–230 µm (approx. 80–90% of the total dermal skeletal thickness). It consists of continuous lamellar tissue, which circumscribes the base and lateral margins of the scale, as well as the visceral ribs. Under crossed polarized light, the lamellar tissue shows corresponding anisiotropic extinction of contiguous lamellae, suggesting the matrix is parallel fibred. The lamellae of the visceral ribs and lateral margins are perforated by a radial fabric of tightly packed linear spaces, each measuring less than 1 µm in diameter (figure 4*d*). Between the median and anterior visceral ribs, the lamellar tissue is highly distorted and contains a fabric of coarse extrinsic fibre spaces, many of which are infilled by pyrite (figure 4*e*). These spaces measure approximately 20–40 µm in length. The fibre fabric is inclined relative to, and cross-cut the lamellae. The spaces are consistent with extrinsic collagen fibre spaces.

#### (iv) Summary

The results of the histological analysis reveal that anaspids exhibit considerable variation, both with respect to the architecture of the dermal skeleton and the microstructure of their component tissues. Yet these diverse dermal skeletons can be rationalized to a hypothetical ancestral morphotype. Anaspid scales are composed primarily of compact concentric lamellar tissue; both optically and histologically compatible with acellular parallel fibre bone. In *Birkenia*, the entire scale body is composed of simple concentric lamellae showing onion-skin development similar to acanthodian scales [30]. The core of the scale, representing the earliest growth increments, contains a coarse intrinsic longitudinally aligned fabric of collagen fibre bundles. In all taxa, a radial fabric of extremely fine linear spaces, described by Gross as ‘Scheitelung’ [19], pervades the acellular parallel fibre bone. This fabric is particularly well-developed directly under the tubercles and within the visceral ribs, the latter because the spaces are often filled with pyrite, increasing the absorption contrast in our srXTM data. The radial fabric is associated with warping of the boundaries between contiguous lamellae, suggesting that the fabric was formed during ossification by distortion of the unmineralized osteoid matrix. In *Rhyncholepis* and *Vesikulepis*, the body of the scales is stratified into two layers: a basal compact layer and an overlying loosely compact vascularized layer. The vasculature comprises a series of longitudinal canals, which open externally via numerous pores between tubercles. The canals are enveloped by complex lamellar tissue that is optically indistinguishable from the underlying compact acellular parallel fibre bone. Gross [19] suggested that the vasculature in *Rhyncholepis*-type anaspids arose via resporption of a simple *Birkenia* like scale. However, we found no evidence of resorption in the vascularized scales of *Rhyncholepis* and *Vesikulepis.* Instead, the lamellae appear to wrap around the canals, indicating apposition and mineralization of lamellae occurred during or after vasculogenesis. Thus, the lack of a canal system in *Birkenia* can be explained by absence of vasculogenesis prior to, or during, appositional growth of acellular parallel fibre bone lamellae.

All surveyed anaspid scales are ornamented with a superficial layer of tubercles composed of spheritic mineralized material. Individual spheres exhibit concentric apposition around a nucleating centre, while the space between spheres is infilled by a discrete homogeneous highly attenuating material. Spheritic mineralization is commonly not only associated with cartilage but it is also encountered in dermal bone [31] and dentine [32], reflecting rapid mineralization in the absence of a coherent matrix [33]. Clearly, spheritic microstructure alone is insufficient to establish tissue homology. Instead homology must also be established on grounds of topology and development. The spheritic tubercle tissue is topologically incompatible with cartilage, which never occurs in the dermal skeleton [34]. Tubercles composed of dermal bone are encountered in antiarch placoderms [31], galeaspids [35] and ctenaspid heterostracans [36], however, the tubercle microstructure in these taxa is indistinguishable from underlying bone. By contrast, the anaspid dermal tubercles form a histologically discrete superficial layer accreted upon, and truncating the microstructure of, a basal layer of bone. Computed tomographic segmentation of the growth history of the scales (e.g. figure 2*f*) shows that the tubercles exhibit odontode-like apposition in association with areal growth of the scale. The high attenuation (bright shades, reflecting relatively high X-ray absorption) of the homogeneous material defining the outer surface of the tubercles and filling the space between calcospheres indicates that this tissue is hypermineralized; its topology, structure and attenuation profile is compatible with enameloid seen in the dermal scales of heterostracans [37], thelodonts [38] and placoderms [39]. Enameloid matrix is of mixed ameoblastic and odontoblastic origin and consequently only develops in contact with dentine [40]. This combination of discriminatory evidence supports the interpretation of the tissue characterized by spheritic mineralization as spheritic (globular) dentine (a ubiquitous mode of dentine mineralization [41]). In most vertebrates, enameloid is topologicaly superficial to tubular dentine, however, the boundary between these tissues is often irregular, e.g. in heterostracans [37] and placoderms [39]. The enameloid–dentine junction comprises a transitional interface in which the dentine organic collagen matrix intertwines with enameloid crystal, resulting in strong cohesion [42]. Anaspid tubercles seem to lack a collagen matrix entirely and this may explain the disorganized interface between these tissues. The histological data obtained from our srXTM study are compatible with the data obtained using LM and SEM (see the electronic supplementary material).

The interpretation of the tissues comprising the superficial tubercles as enameloid and dentine also supports the interpretation of these structures as odontode derivatives. The alternative hypothesis that the spheritic tissue represents a type of dermal bone derived from a non-collagenous matrix, is less plausible because of its topology and its failure to explain the presence of the hypermineralized interstitial tissue.

### (b) Phylogenetic analyses

**Figure 5.**
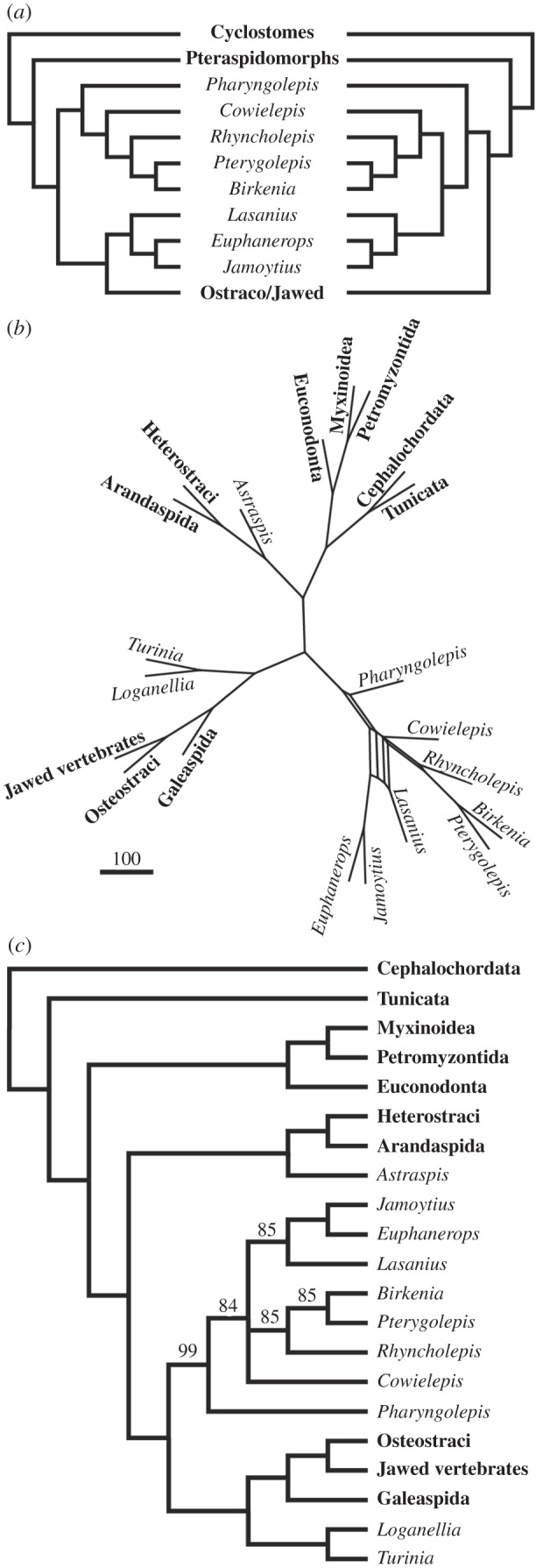
Results of the phylogenetic analyses. (*a*) Two most parsimonious trees recovered from an equal weights TBR search in TNT v.1; (*b*) consensus network of 100 SCC trees derived from 100 different weighting parameters (including *k* = ∞). Edge lengths are proportional to the number of weighting parameters which recover the edge; (*c*) 50% majority rule consensus of 100 SCC trees derived from 100 different weighting parameters (including *k* = ∞). Branch annotations equal the number of weighting parameters that recover the given branch.

A tree bisection and reconnection (TBR) search yielded two most parsimonious trees (MPTs) at 174 steps (figure 5*a*,*b*). In both trees, cyclostomes + conodonts are recovered as the sister group to total-group gnathostomes. Within total-group gnathostomes, pteraspidomorphs are recovered as the most deeply branching clade. Anaspids are recovered either as a grade (figure 5*a*) or a clade (figure 5*b*). In both cases, anaspids subtend a clade comprising thelodonts, galeaspids, osteostracans and jawed vertebrates. To further test the phylogenetic placement of anaspids, we conducted Templeton tests and one-tailed Kishino–Hasegawa tests (approximate Shimodaira–Hasegawa test) in order to evaluate whether the data are decisive between alternative hypotheses of topology (see the electronic supplementary material, table S1). These reject the hypothesis that anaspids are the sister group of jawed vertebrates, but fail to reject alternative hypotheses of anaspid affinity.

In order to assess the impact of weighting parameters on tree topology, the dataset was reanalysed in TNT under equal weighting (concavity constant (*K*) = ∞) and implied weighting at 99*K* values derived from a log-normal distribution. MPTs generated under each concavity constant are recorded in the electronic supplementary material. The results of the implied weighting analysis show that all MPTs under all concavity constants resolve anaspids as the sister group of thelodonts + galeaspids + osteostracans + jawed vertebrates, with pteraspidomorphs subtending this clade as the most deeply branching stem-gnathostomes. Anaspids are recovered as a clade in all MPTs under all concavity constants (with the exception of 1 MPT under equal weighting, which we have discussed above). Different weighting paramaters recover conflicting topologies within anaspids. Most notably, at low values of *K* (less than 1), the naked anaspids *Jamoytius* and *Euphanerops* are recovered as the most deeply branching clade of anaspids, while at higher values of *K* (greater than 1), they are recovered as nested within more extensively skeletonized anaspids, as the sister group of *Lasanius*. *K*-values below 1 are generally perceived as unrealistic because they suggest characters with multiple steps across the tree possess no phylogenetic signal [43]. Conflicting topologies supported by different values of *K* can be visualized using a splits consensus network of SCC trees derived from 100*K* values (figure 5*c*). In this diagram, edge lengths are proportional to the number of concavity constants that support the edge. The data can also be displayed using a majority rule consensus tree (figure 5*d*). In this diagram, node annotations correspond to the number of weighting parameters that support the branch.

## 5. Discussion

### (a) Phylogenetic affinity of anaspids

The results of our phylogenetic analyses support the placement of anaspids as stem-gnathostomes, though topology tests do not allow us to reject the possibility that anaspids are stem-cyclostomes. Topology tests also fail to discriminate between whether anaspids or pteraspidomorphs constitute the most deeply branching clade of stem-gnathostomes. Yet while these scenarios appear to be equally good explanations of the data under equal weighting, only one of these hypotheses, that anaspids are the sister group of thelodonts, galeaspids, osteostracans and jawed vertebrates, is recovered under all weighting parameters. This indicates that transformation costs are optimized and, as such, it is a superior explanation of the data. The results of the phylogenetic analyses, therefore, suggest that it is the pteraspidomorphs, rather than the anaspids, that are the earliest branching lineage of stem-gnathostomes.

### (b) Evolutionary significance of the anaspid skeleton

Resolution of anaspid phylogenetic affinity allows us to interpret their histology within the context of vertebrate dermal skeleton evolution. Anaspids primitively possess a superficial layer of tubercles, here interpreted as dermal odontodes. These are composed of a mixed layer of enameloid and spheritic dentine. The sister group of anaspids (thelodonts + jawed vertebrates) also primitively possess dermal odontodes (secondarily lost in galeaspids) [35,37,39,44], however, these are composed of tubular dentine capped with a thin layer of enameloid. Comparable stratified dermal odontodes are possessed by pteraspidomorphs, the most deeply branching skeletonizing vertebrates [37,45]. Thus, based on the phylogenetic distribution of odontode microstructures, we infer the ancestor of skeletonizing vertebrates possessed dermal odontodes composed of tubules dentine and capped with enameloid. We interpret the spheritic microstructure of anaspid dermal odontodes as appomorphic.

All surveyed anaspids possess polyodontode scales. In *Rhyncholepis* and *Vesikulepis*, the odontodes are supplied by a pervasive underlying canal system. Polyodontode scales supplied by canal systems are also characteristic of heterostracan, osteostracan and jawed vertebrate scales [37,46]. As such, we infer that scales in the ancestor of skeletonizing vertebrates were polyodontode, supplied by a pervasive underlying canal systems.

Anaspids lack an extensive osteonal middle layer capable or resorption. This character is possessed by osteostracans and jawed vertebrates [39], but is absent in galeaspids and thelodonts [35,37,44]. However, pteraspidomorphs primitively possess an extensive osteonal layer of polygonal cancellae that exhibits evidence of resorption [37]. This indicates that either the ancestor of skeletonizing vertebrates possessed an extensive osteonal middle layer subsequently lost in anaspids, thelodonts and galeaspids, or else an extensive osteonal middle layer evolved independently in pteraspidomorphs and osteostracans + jawed vertebrates. While the independent evolution of an osteonal layer within two vertebrate lineages may seem implausible, this solution is more parsimonious than alternative scenarios. As such, it is perhaps more plausible that the plesiomorphic vertebrate dermal skeleton lacked such a layer.

Anaspids primitively possess a laminated basal layer composed of acellular parallel fibre bone. Pteraspidomorphs, galeaspids, osteostracans and jawed vertebrates also possess a laminated basal layer, although in these taxa it is composed of isopedine—a type of laminated tissue in which the collagen fibrils are organized into orthogonal bundles much like plywood [35,37,44]. Thelodonts lack a mineralized basal laminated layer; their dermal skeletons correspond only to the superficial layer [44]. Given that a laminated basal layer is present in all skeletonizing vertebrate lineages (with the exception of thelodonts), it seems likely that it was manifest in the primitive vertebrate dermal skeleton. Anaspids are phylogentically bracketed by taxa that possess basal isopedine. Consequently, we interpret the acellular parallel fibre bone organization of the anaspid basal layer as independently derived.

Not all anaspids possess an extensive mineralized dermal skeleton. The so-called naked anaspids ostensibly lack mineralized scales and plates; their elongate bodies are instead subdivided into W, Z or V-shaped serially repeating segments. Naked anaspids have frequently been interpreted as more closely related to lampreys than to other anaspids, owing to their apparent lack of mineralized tissues, their elongate slanting branchial series and, in the case of *Jamoytius*, the presence of terminal structures thought to be composed of cartilage [3,6]. However, recent work by Sansom *et al.* [5] has cast some doubt on the validity of these characters. For instance, the supposition that *Jamoytius* was ‘naked’ relies on an interpretation of the W, Z or V-shaped serially repeating trunk segments as myomeres. Yet microscopic reanalysis of the segments in *Jamoytius* found no trace of structures compatible with muscle fibres*.* Instead, the authors report a dendric pattern of phosphatic material, interpreted as the result of splitting through thin, weakly mineralized scales [5]. The identity of the segments in *Euphanerops* remains unresolved. If these structures are interpreted as weakly mineralized scales, they must have been strongly anchored to the underlying musculature, as they do not occur scattered around the body, even in disarticulated specimens. Our phylogenetic analyses place *Jamoytius* and *Euphanerops*, together with the equally enigmatic taxon *Lasanius*, as nested within a monophyletic Anaspida, contrary to some previous studies [2,5,9]. Consequently, we interpret the poorly mineralized dermal skeletons in these taxa as a derived state within anaspid evolution, rather than representing the plesiomorphic condition in anaspids, or vertebrates more generally.

**Figure 6.**
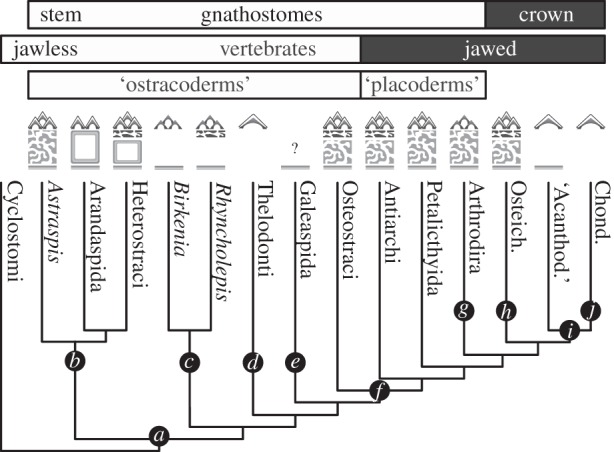
Evolution of the dermal skeleton within total group gnathostomes. (*a*) Origin of a mineralized skeleton, origin of odontodes; (*b*) acellular osteons; (*c*) spheritic denine; (*d*) loss of basal layer; (*e*) loss of odontodes; (*f*) origin of cellular osteons; (*g*) loss of enameloid; (*h*) origin of true enamel; (*i*) loss of cellular osteons and (*j*) loss of basal layer.

Based on the phylogenetic distribution of characters (figure 6), we suggest that the plesiomorphic vertebrate dermal skeleton was bipartite, consisting of a polyodontode superficial layer composed of tubular dentine and capped by enameloid overlying a laminated basal layer of isopedine. This ancestral skeleton was subsequently modified independently within each major skeletonizing vertebrate lineage. Pteraspidomorphs and osteostracans + jawed vertebrates independently evolved a tripartite skeleton via the addition of an extensive intermediate layer composed of centripetal osteons. In pteraspidomorphs, these osteons were primitively acellular [37], where as in osteostracans + jawed vertebrates the osteons primitively contained osteocyte lacunae [44]. The basal isopedine layer was lost within the lineage leading to thelodonts, leaving only the superficial layer of odontodes [44]. By contrast, the superficial odontode layer was lost in the lineage leading to galeaspids so that only the basal isopedine layer remains [35]. The ancestral biapartite skeleton was retained in the lineage leading to anaspids, although each of the component tissues appears to have become secondarily simplified. Anaspids are characterized by loss of a stratified superficial layer, loss of dentine tubules and a reduction in complexity of the collagen matrix within the basal laminated layer. Within anaspids, the dermal skeleton was reduced in several lineages. The *Birkenia* + *Pterygolepis* clade is characterized by the loss of a shallow canal system, whereas *Jamoytius +* *Euphanerops* + *Lasanius* clade shows reduced mineralization.

## 6. Conclusion

Anaspids possess a character complement that challenges our expectations of a neat hierarchal series of nested innovations leading to jawed vertebrates. As such, these intriguing fossil fishes have proved difficult to place within a phylogenetic context. At the same time, palaeohistologists have struggled to interpret their skeletal remains, due to their simple construction and homogeneous appearance. We have re-characterized the skeletal histology of representatives spanning anaspid diversity using srXTM. Our results reveal that anaspids primitively possess a stratified dermal skeleton comprising an odontogenic layer of spheritic tubercles, overriding a basal laminated layer. Phylogenetic analyses place anaspids as a monophyletic group nested within skeletonizing vertebrates. As such, we interpret their dermal skeleton as apomorphic, rather than reflecting the primitive vertebrate dermal skeleton. We reject previous hypotheses that anaspids are stem-lampreys or the sister group to jawed vertebrates.

## Supplementary Material

Electronic Supplementary Information 1 - Histology

## Supplementary Material

Electronic Supplementary Information 2 - Phylogenetics

## Data accessibility

Synchrotron reconstructions used in this manuscript are archived at http://dx.doi.org/10.5523/bris.1sog3n5dupyvs1vzwdwdk191hs. The phylogenetic matrix is archived at http://dx.doi.org/10.5061/dryad.k2qc4.
