# Supplementary material for: Histology and affinity of anaspids, and the early evolution of the vertebrate dermal skeleton
Source: Proc Biol Sci. 2016 Mar 16;283(1826):20152917. doi: 10.1098/rspb.2015.2917 (PMC4810860; doi:10.1098/rspb.2015.2917)
Supplement: Electronic Supplementary Information 1 - Histology [file rspb20152917supp1.pdf]

Supplementary Information

1. Histology

Birkenia

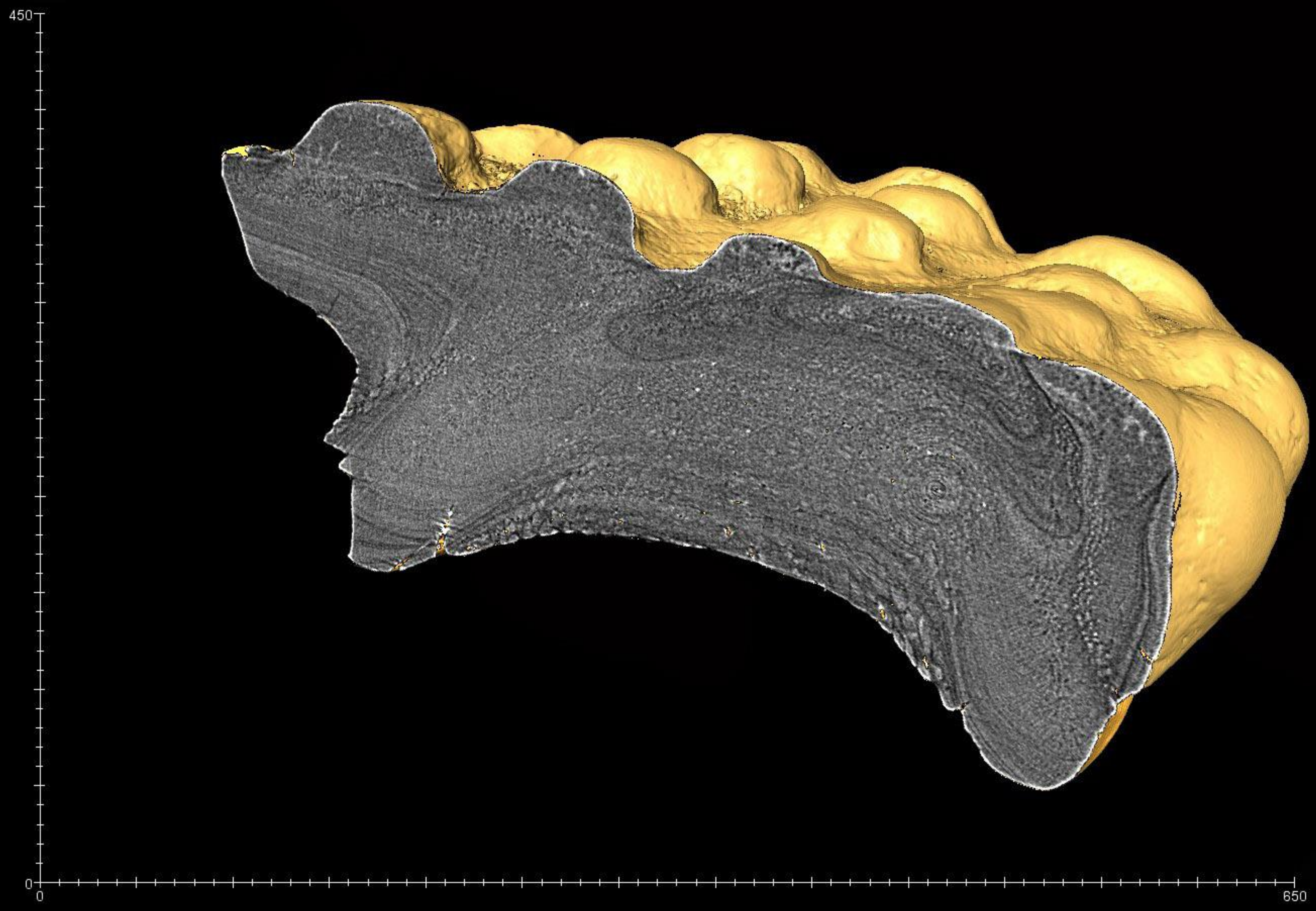

Fig. 1. Specimen NHMUK PV P73701. SRXTM slice through a body scale of *Birkenia*. Data collected using 20X objective. Scale bars in µm.

Birkenia

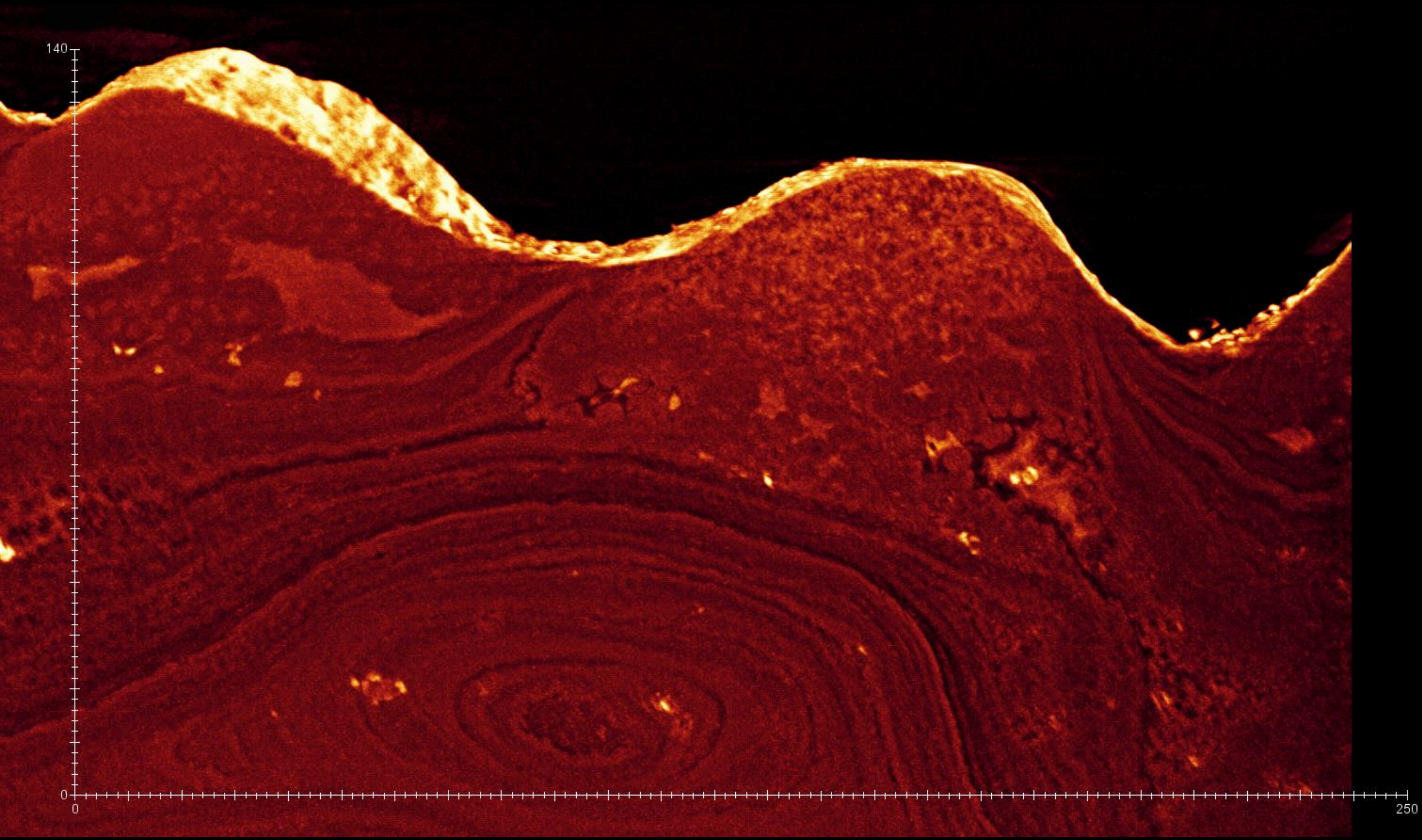

Fig. 2. Specimen ANASP_02. SRXTM volume rendered thin section through the superficial layer of *Birkenia*. Data collected using 40X objective. Scale bars in μm.

Birkenia

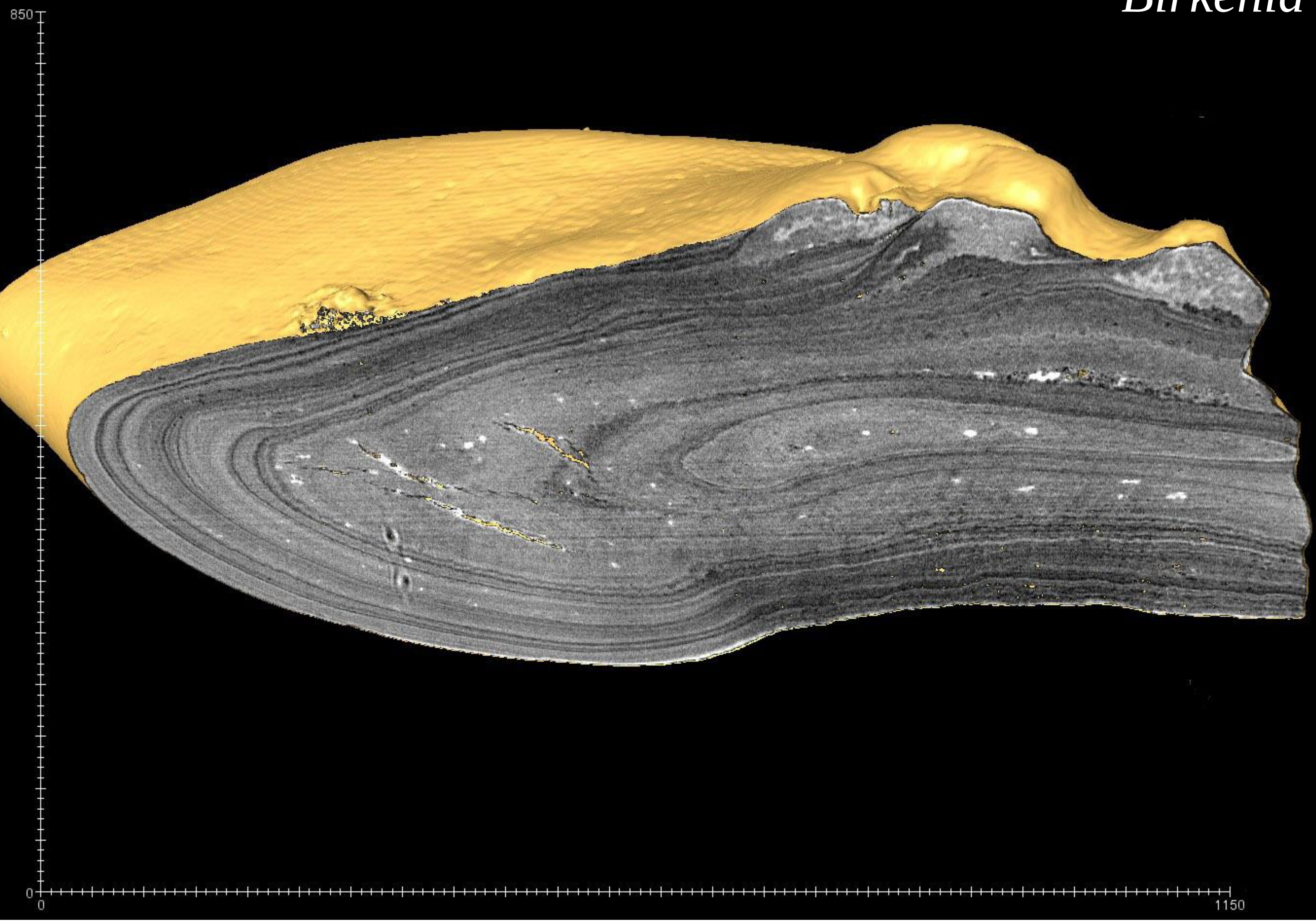

Fig. 3. Specimen ANASP_02. SRXTM longitudinal slice through a body scale of *Birkenia*. Data collected using 10X objective. Scale bars in μm.

Birkenia

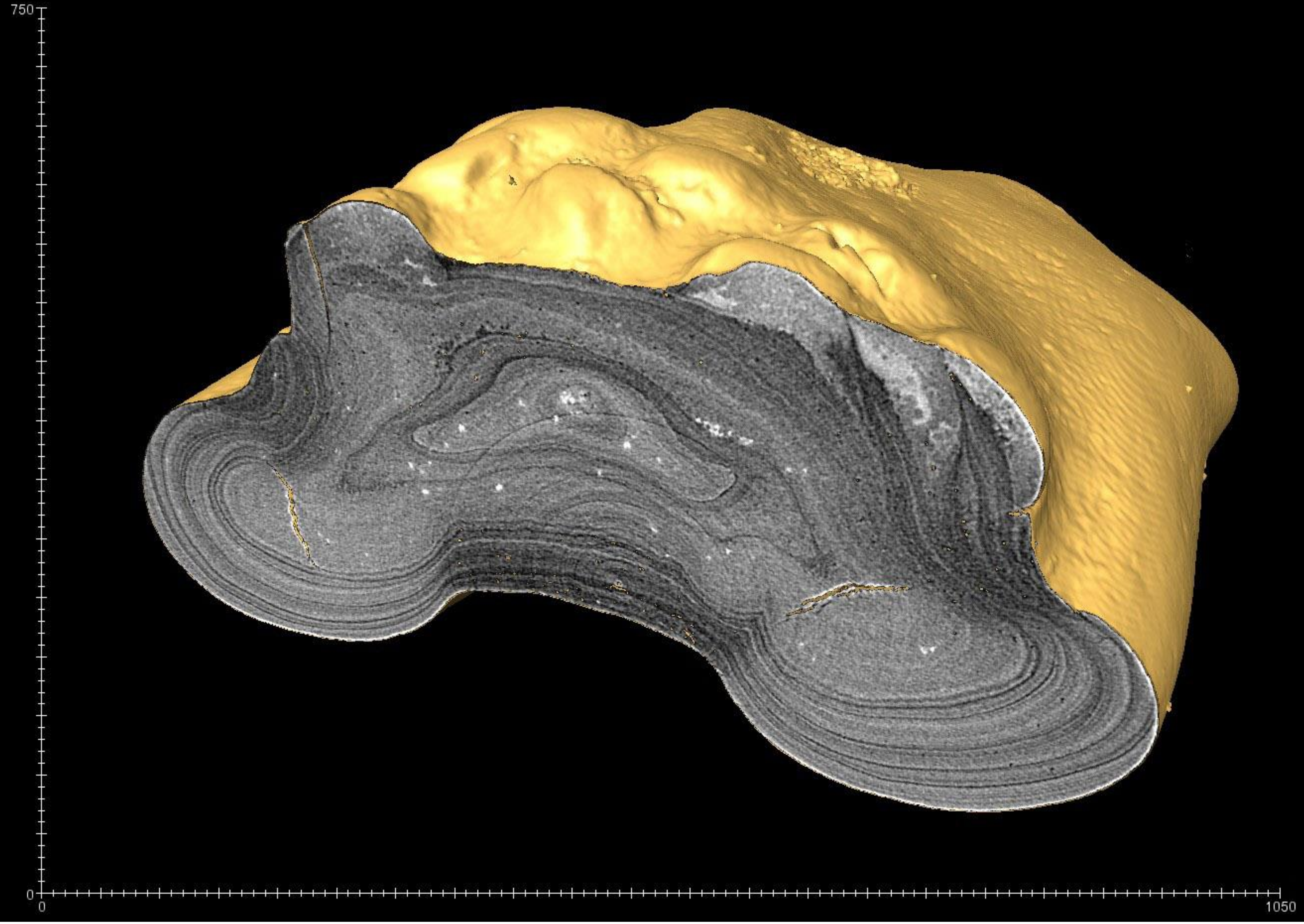

Fig. 4. Specimen ANASP_02. SRXTM slice through a body scale of *Birkenia*. Data collected using 10X objective. Scale bars in μm.

Birkenia

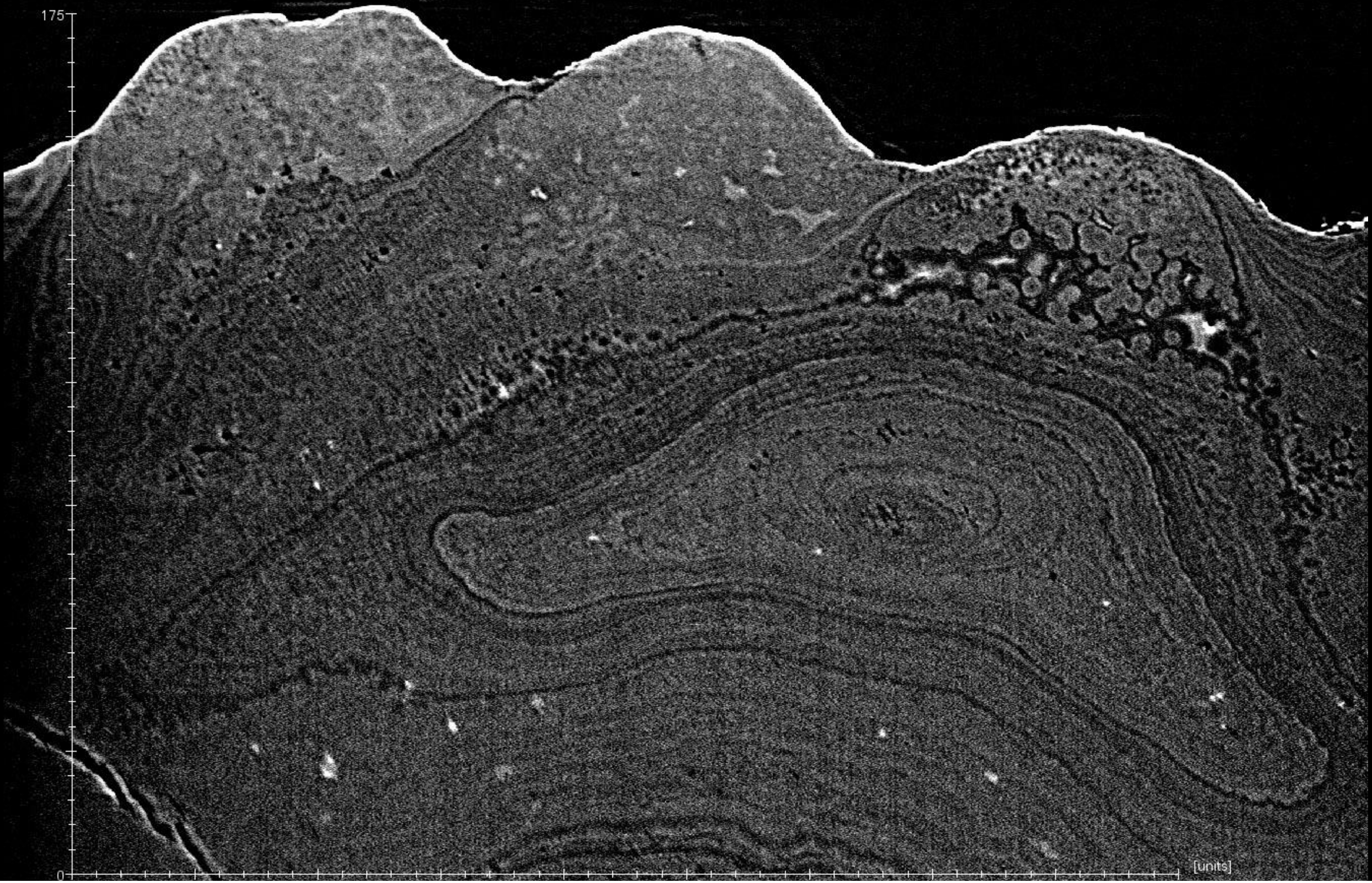

Fig. 5. Specimen ANASP_02. SRXTM slice through the superficial layer of *Birkenia*. Data collected using 40X objective. Scale bars in μm.

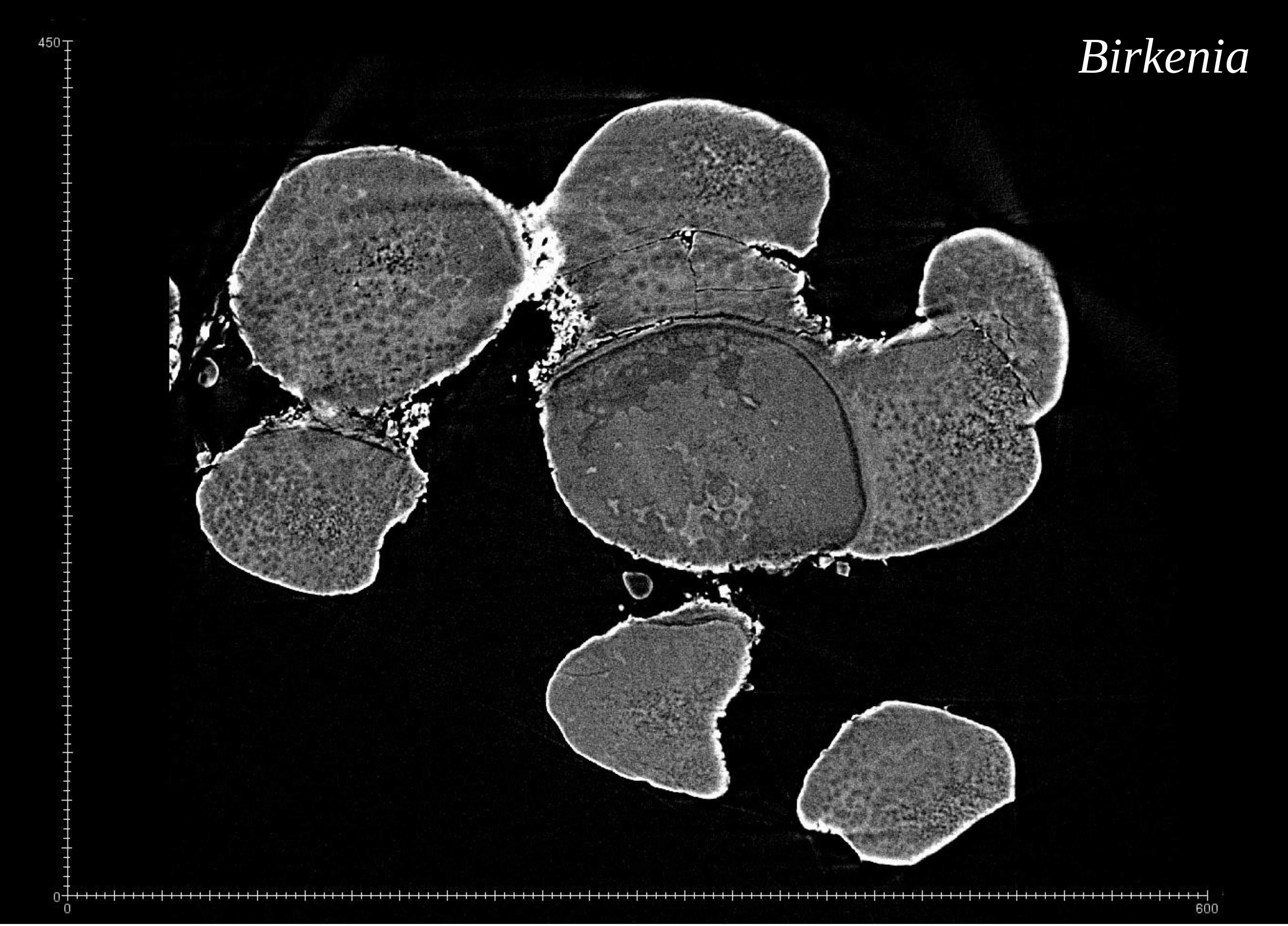

Fig. 6. Specimen ANASP_02. SRXTM horizontal slice through the superficial layer of *Birkenia*. Data collected using 40X objective. Scale bars in μm.

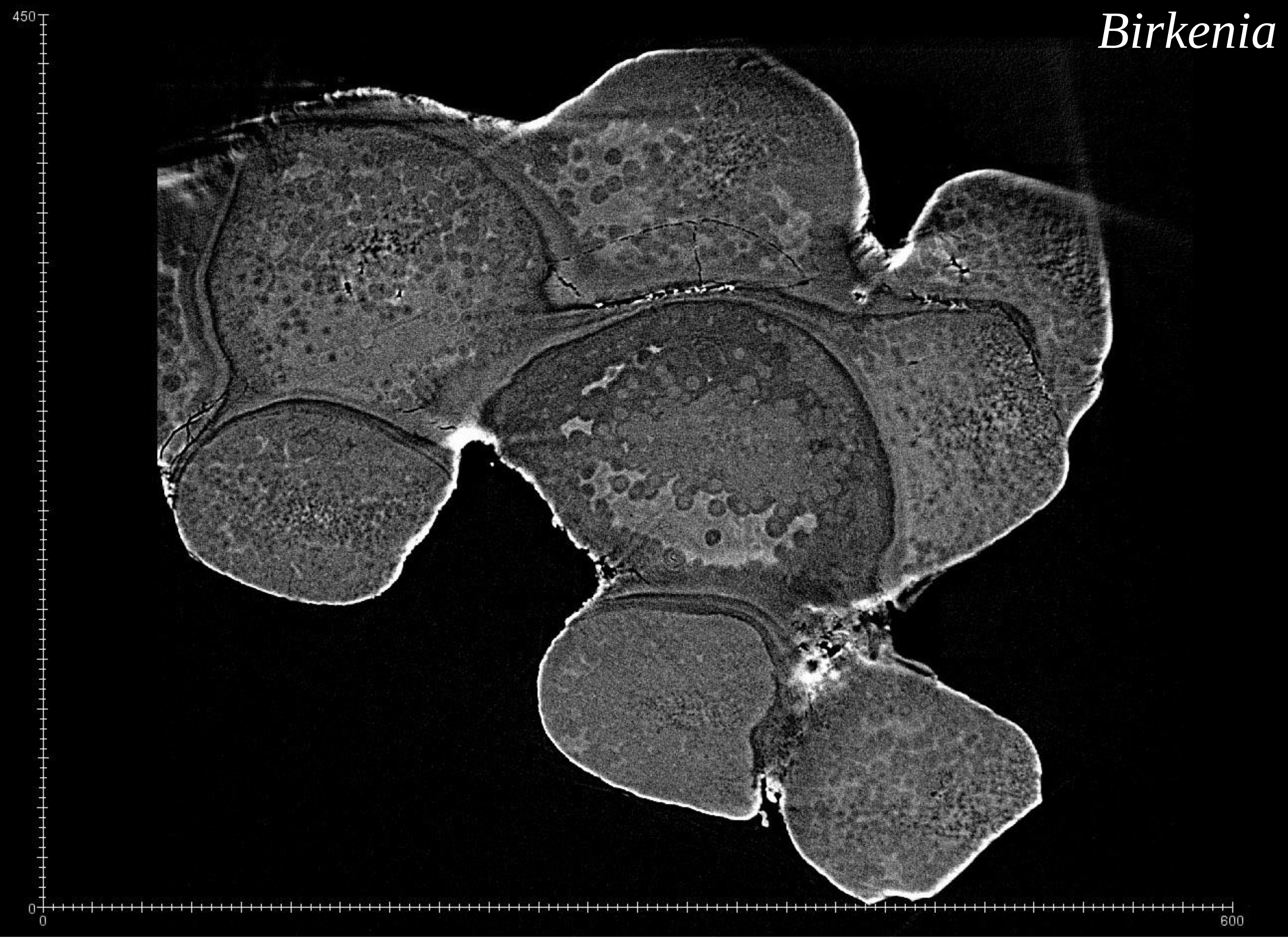

Fig. 7. Specimen ANASP_02. SRXTM horizontal slice through the superficial layer of *Birkenia*. Data collected using 40X objective. Scale bars in μm.

Birkenia

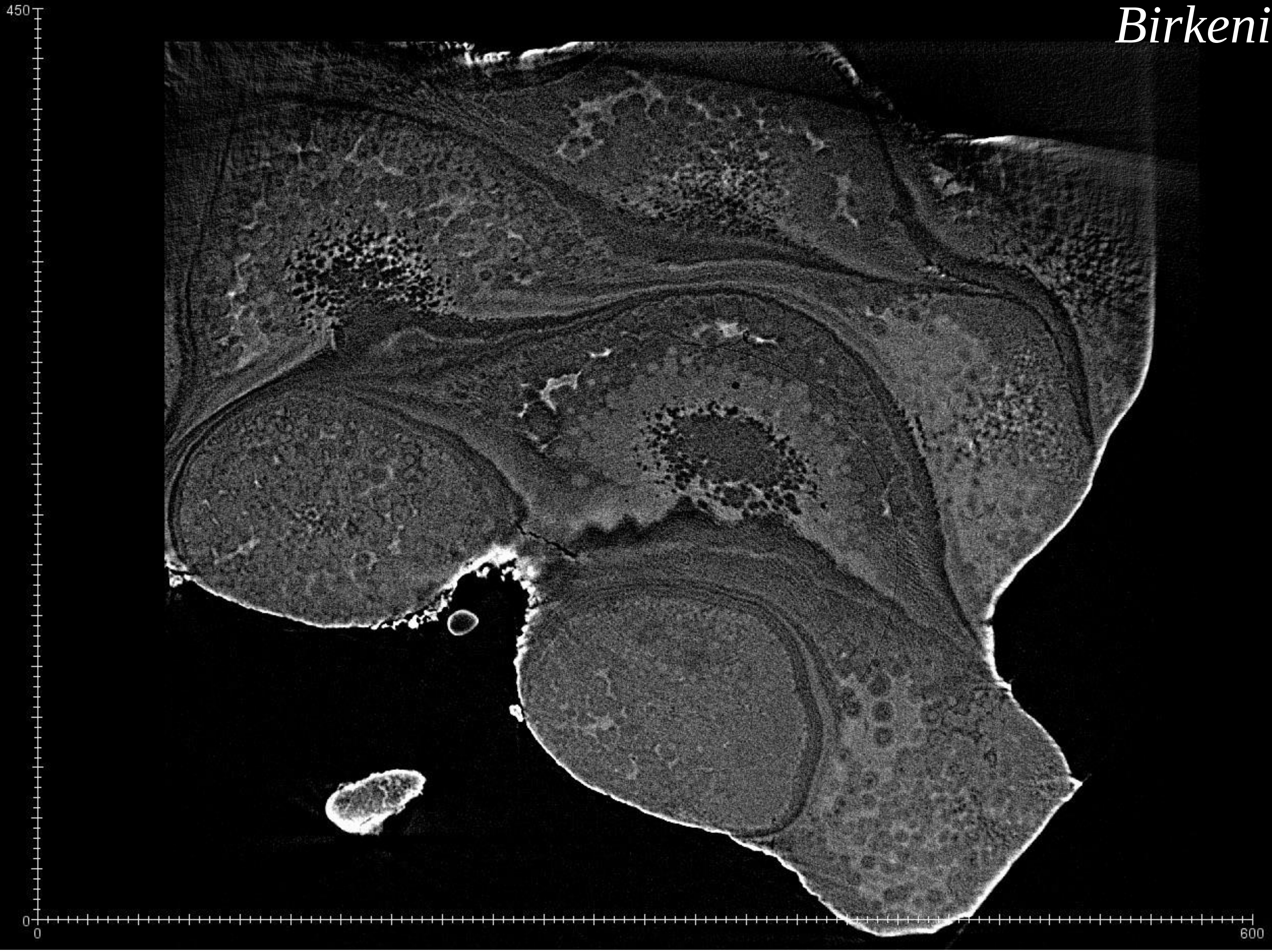

Fig. 8. Specimen ANASP_02. SRXTM horizontal slice through the superficial layer of *Birkenia*. Data collected using 40X objective. Scale bars in μm.

Birkenia

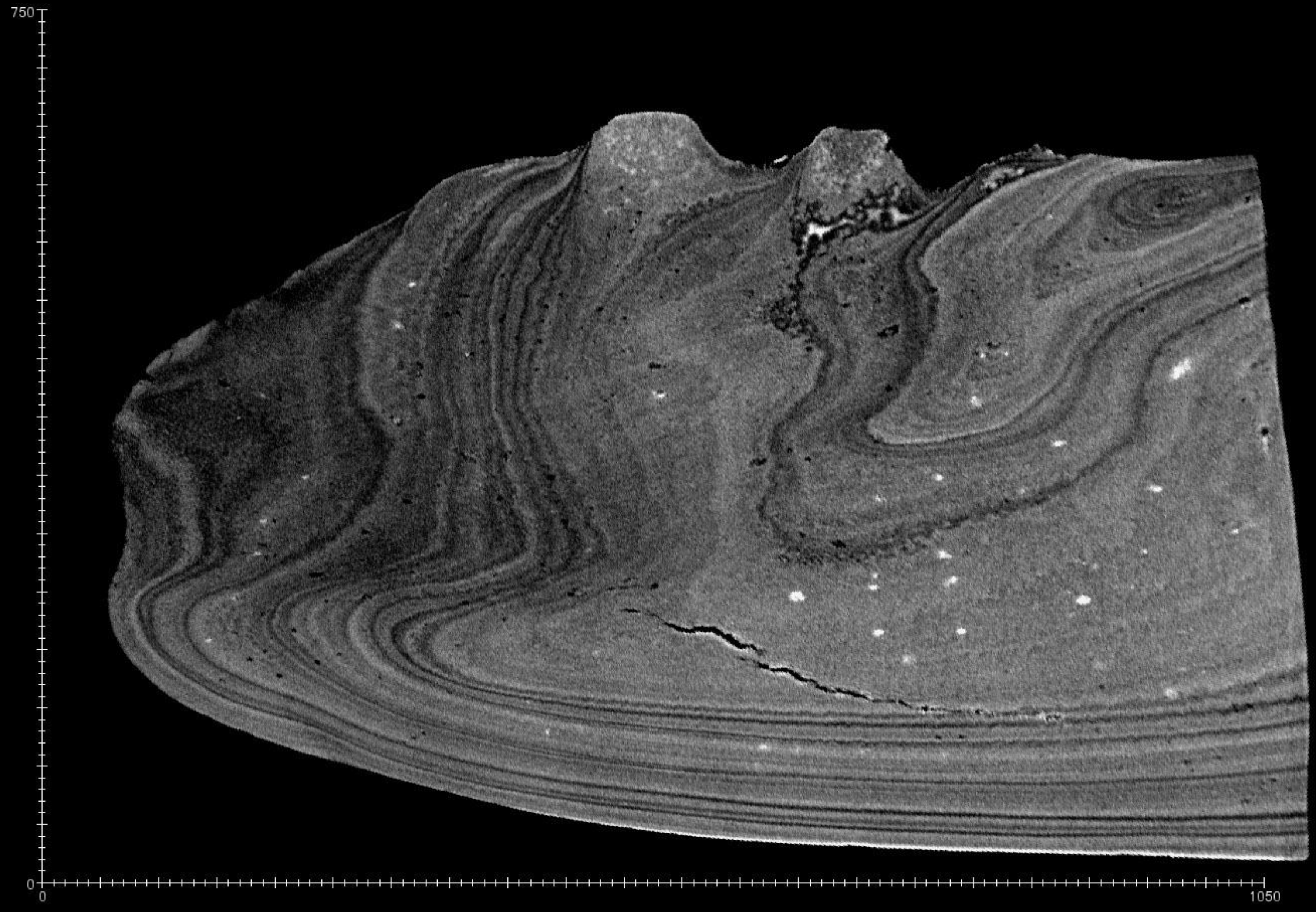

Fig. 9. Specimen ANASP_02. SRXTM longitudinal slice through a body scale of *Birkenia*. Data collected using 10X objective. Scale bars in μm.

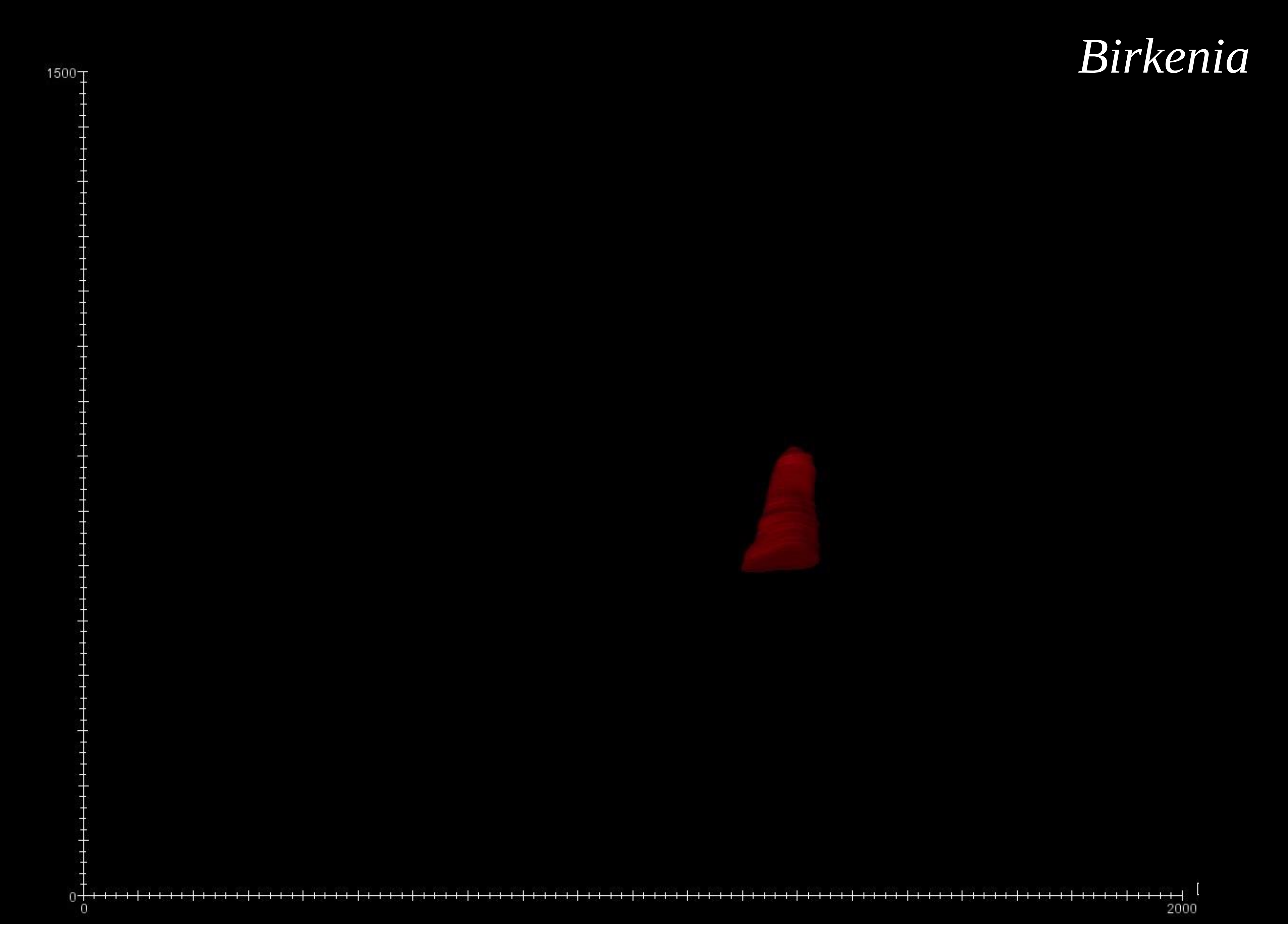

Fig. 10. Specimen ANASP_02. SRXTM segmentation of discrete growth increments. Data collected using 10X objective. Scale bars in μm.

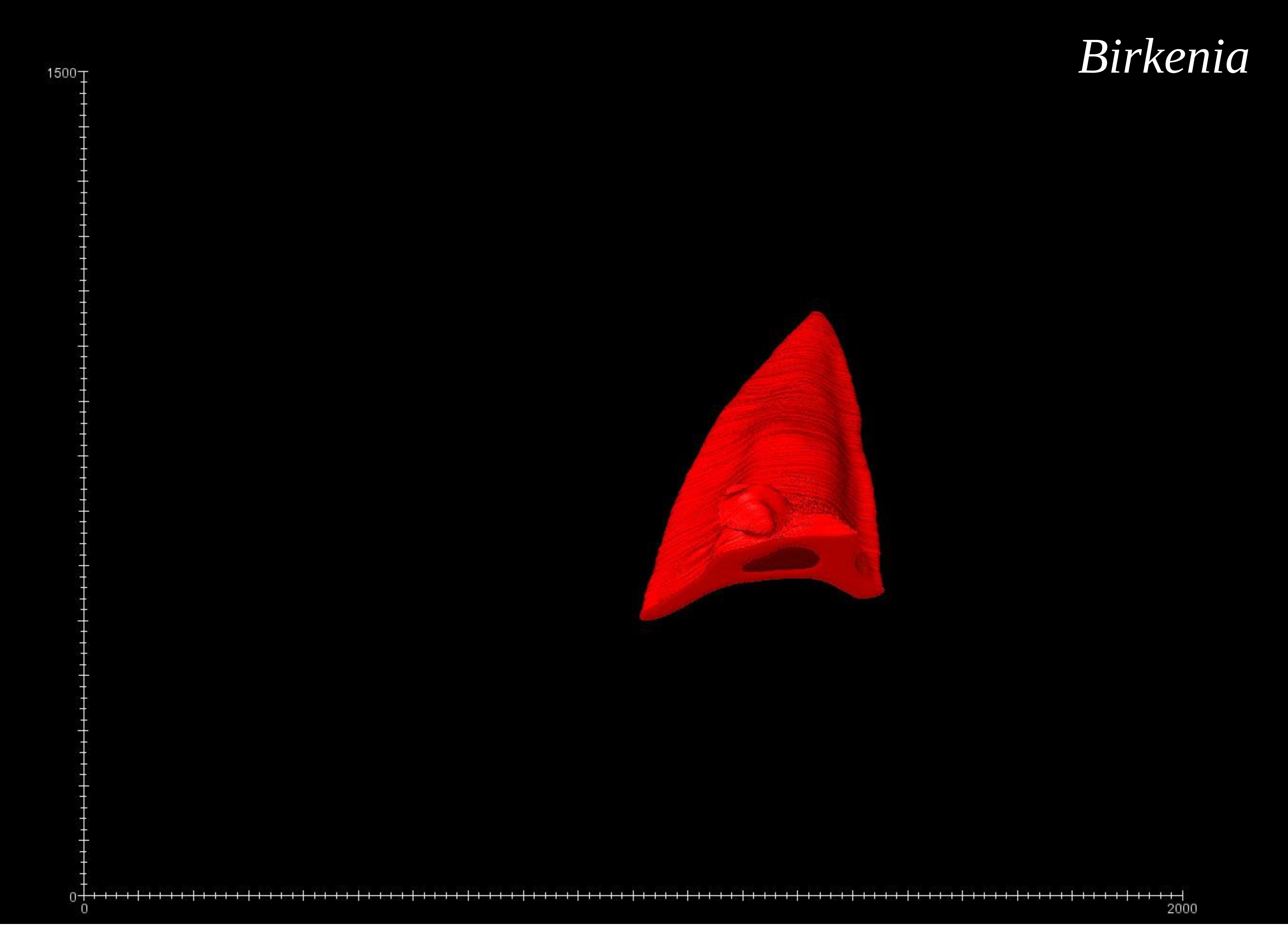

Fig. 11. Specimen ANASP_02. SRXTM segmentation of discrete growth increments. Data collected using 10X objective. Scale bars in μm.

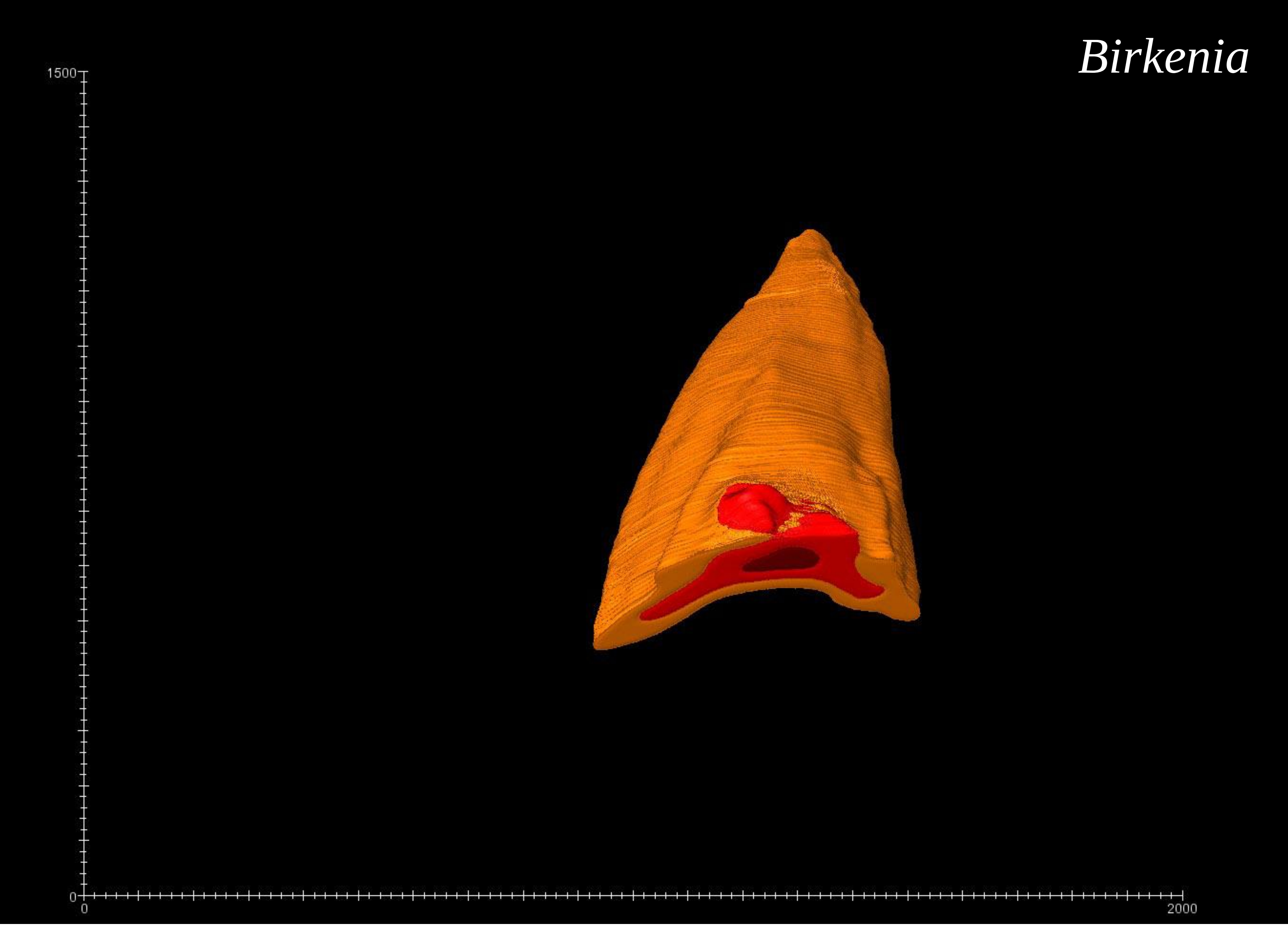

Fig. 12. Specimen ANASP_02. SRXTM segmentation of discrete growth increments. Data collected using 20X objective. Scale bars in μm.

Birkenia

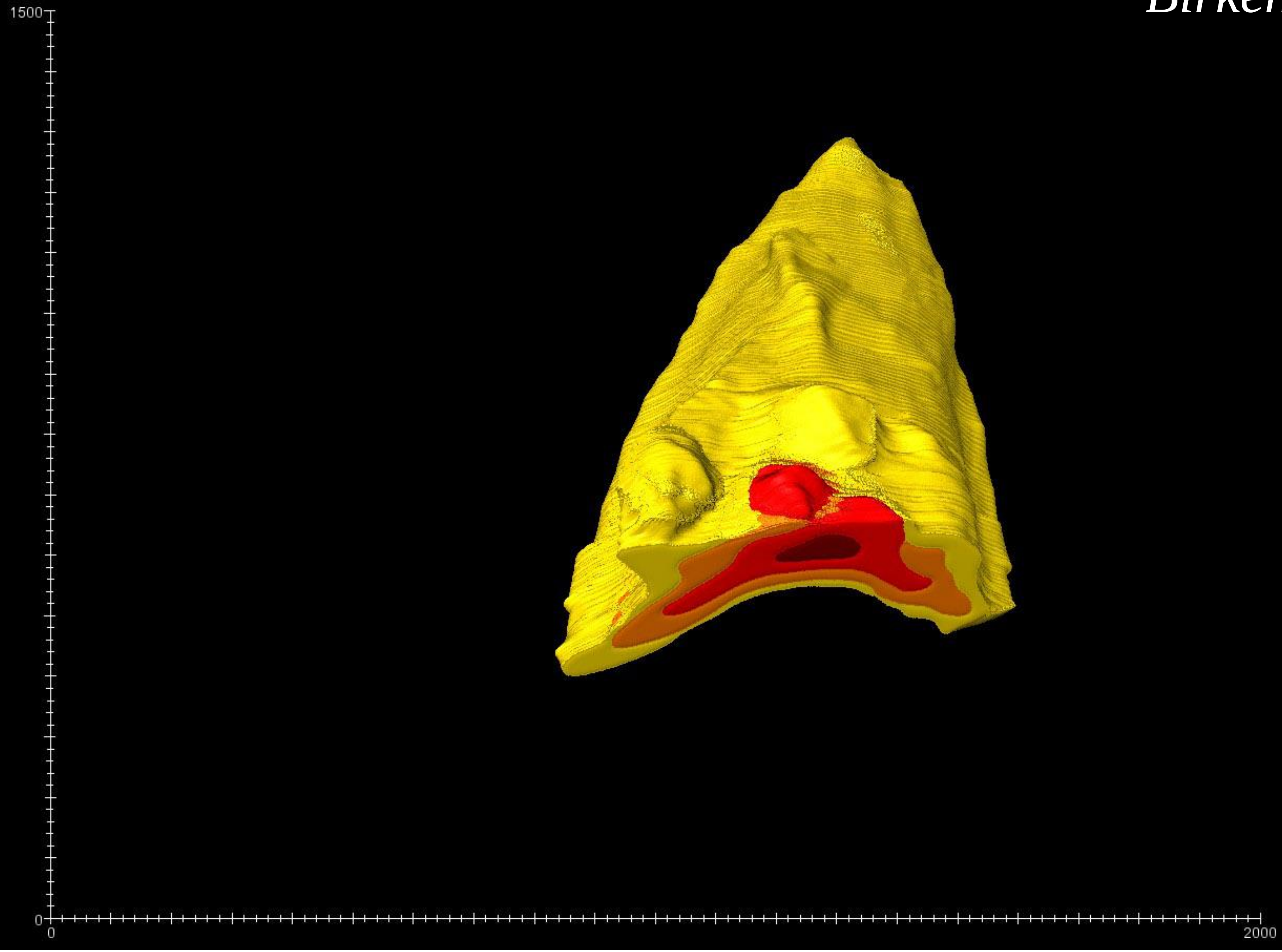

Fig. 13. Specimen ANASP_02. SRXTM segmentation of discrete growth increments. Data collected using 10X objective. Scale bars in μm.

Birkenia

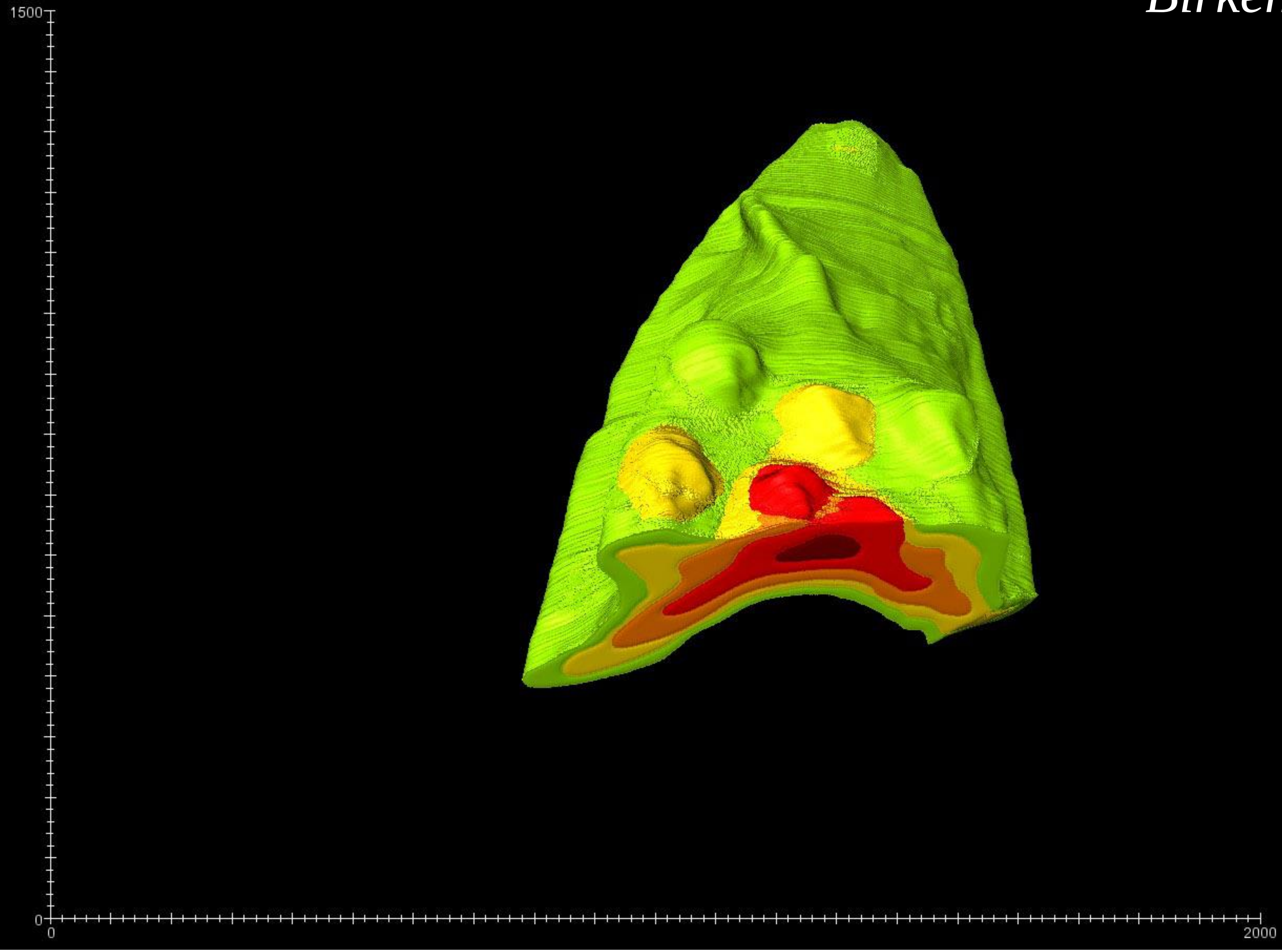

Fig. 14. Specimen ANASP_02. SRXTM segmentation of discrete growth increments. Data collected using 10X objective. Scale bars in μm.

Birkenia

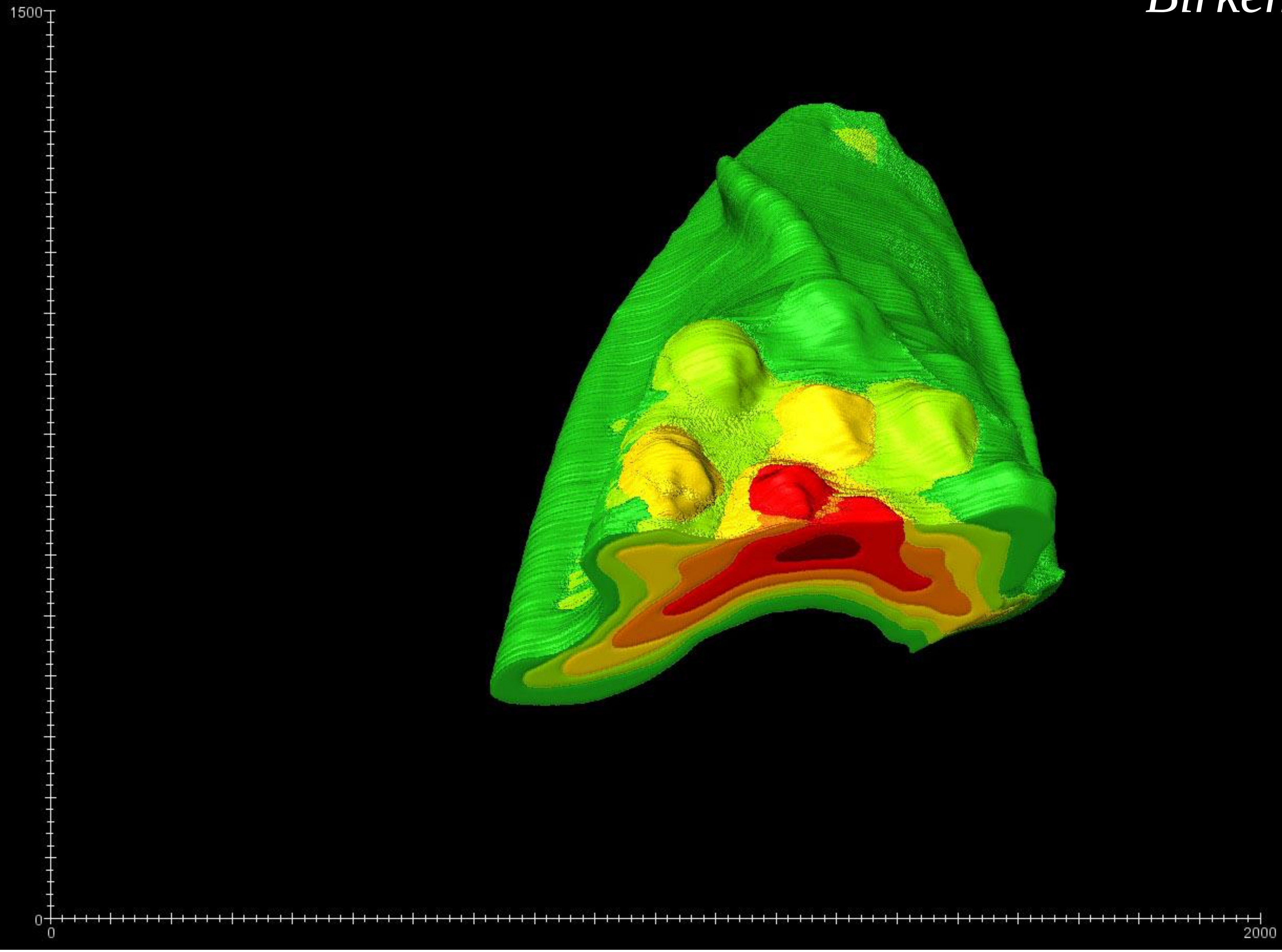

Fig. 15. Specimen ANASP_02. SRXTM segmentation of discrete growth increments. Data collected using 10X objective. Scale bars in μm.

Birkenia

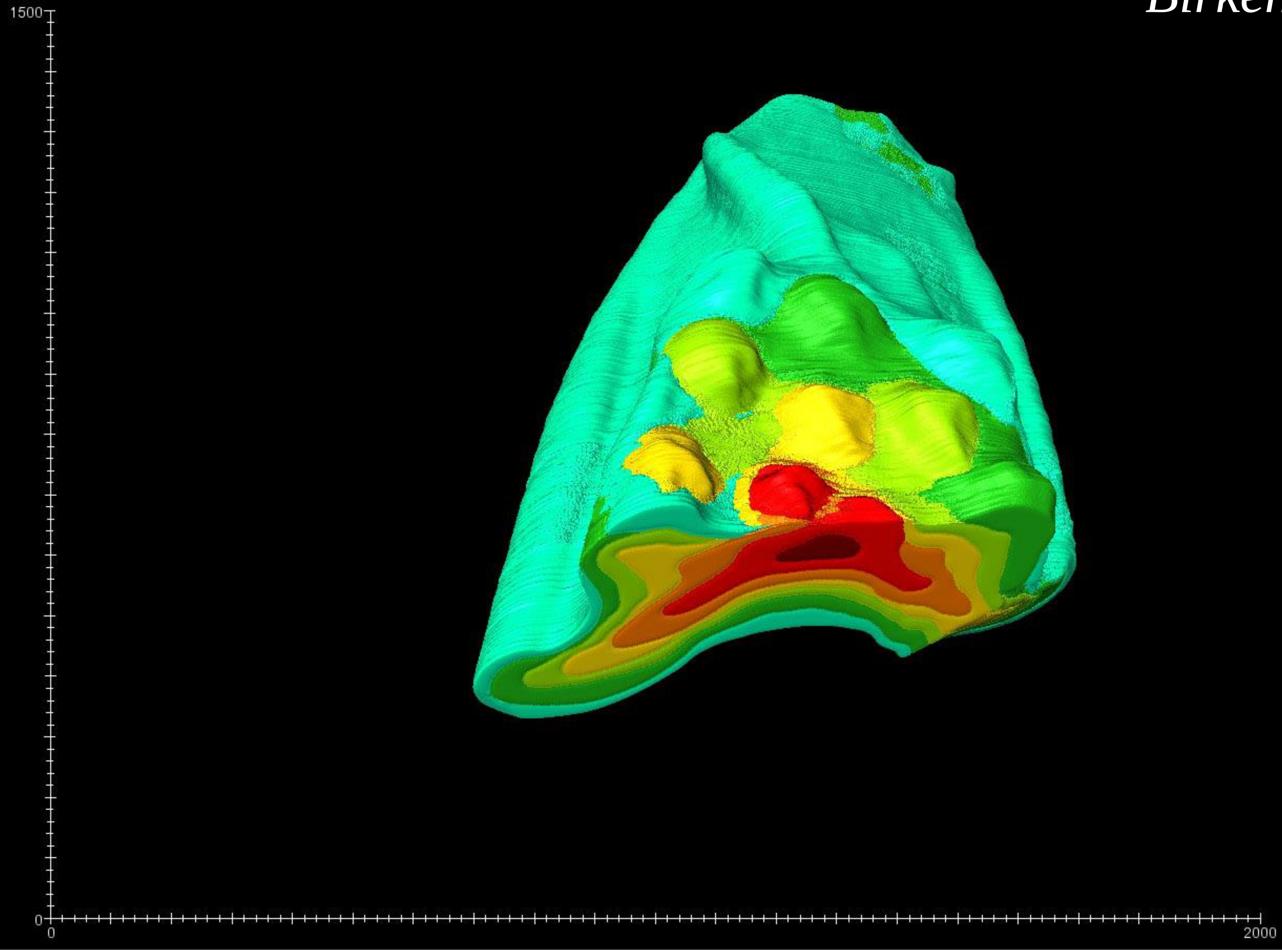

Fig. 16. Specimen ANASP_02. SRXTM segmentation of discrete growth increments. Data collected using 10X objective. Scale bars in μm.

Birkenia

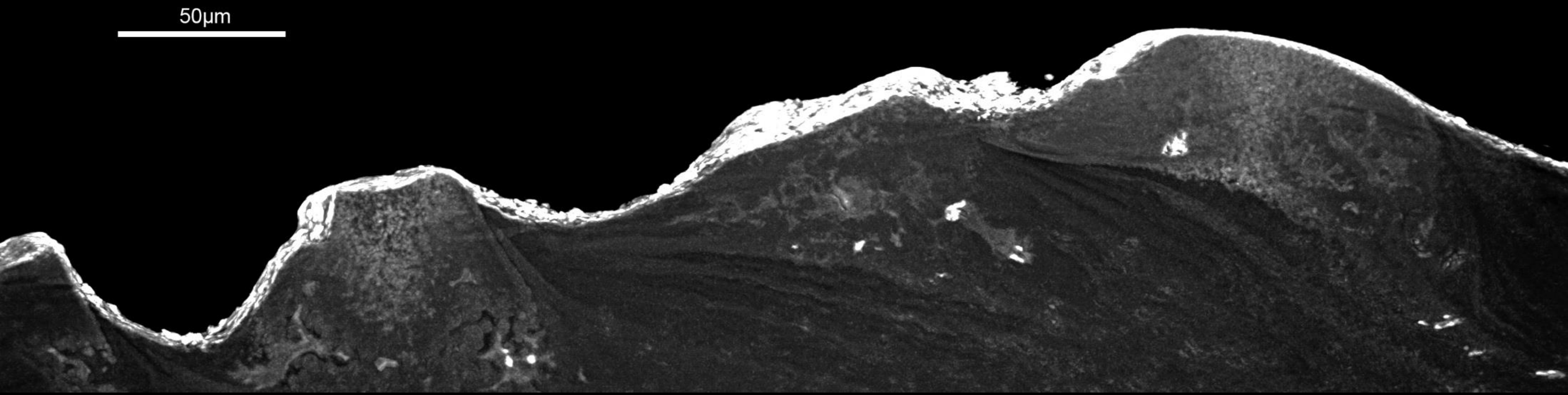

Fig. 17. Specimen ANASP_02. SRXTM volume rendered thin section of the superficial layer. Data collected using 40X objective. Scale bars in µm.

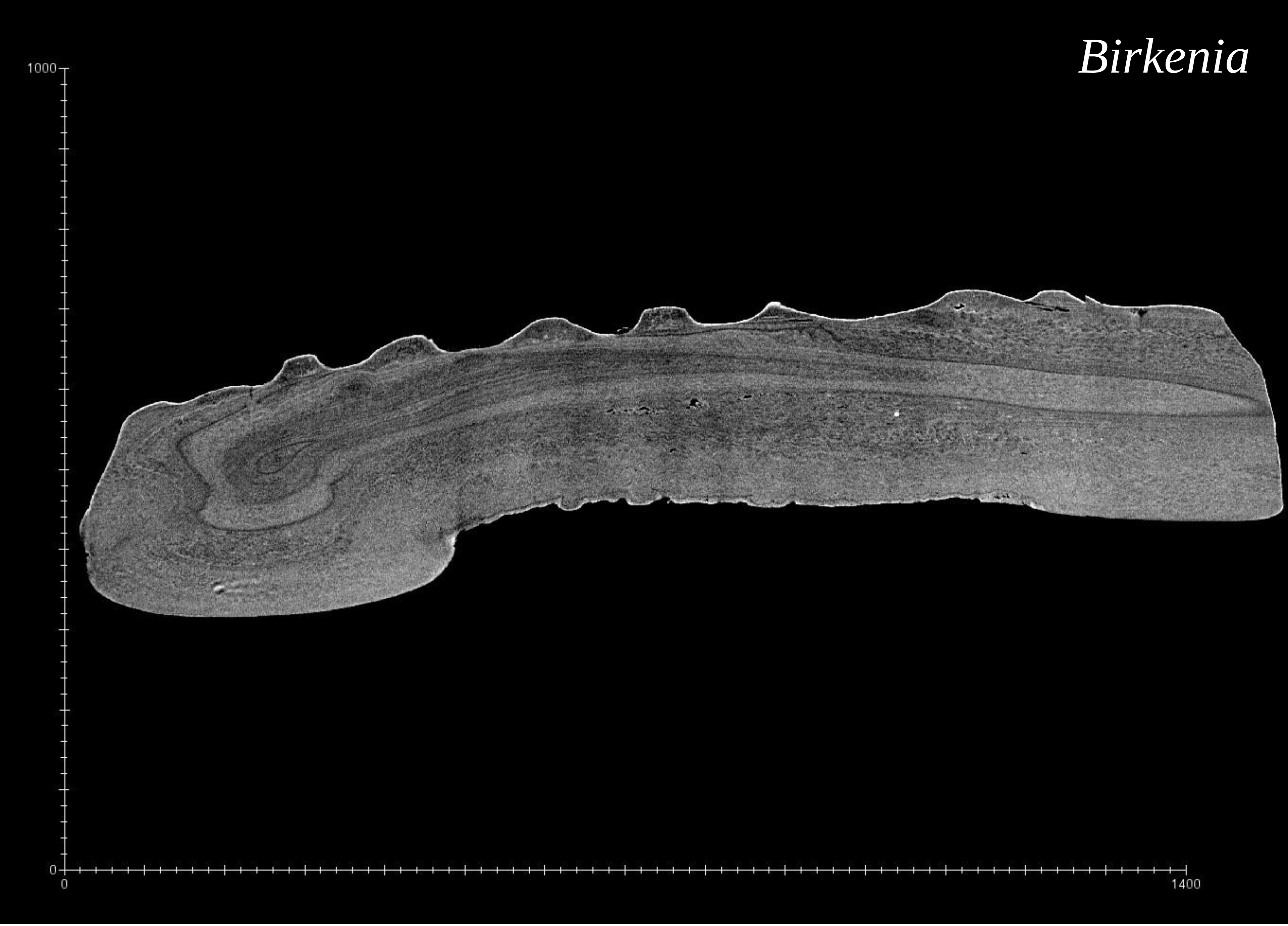

Fig. 18. Specimen NHMUK PV P73702. SRXTM longitudinal slice through a body scale of *Birkenia.* Data collected using 10X objective. Scale [illegible]

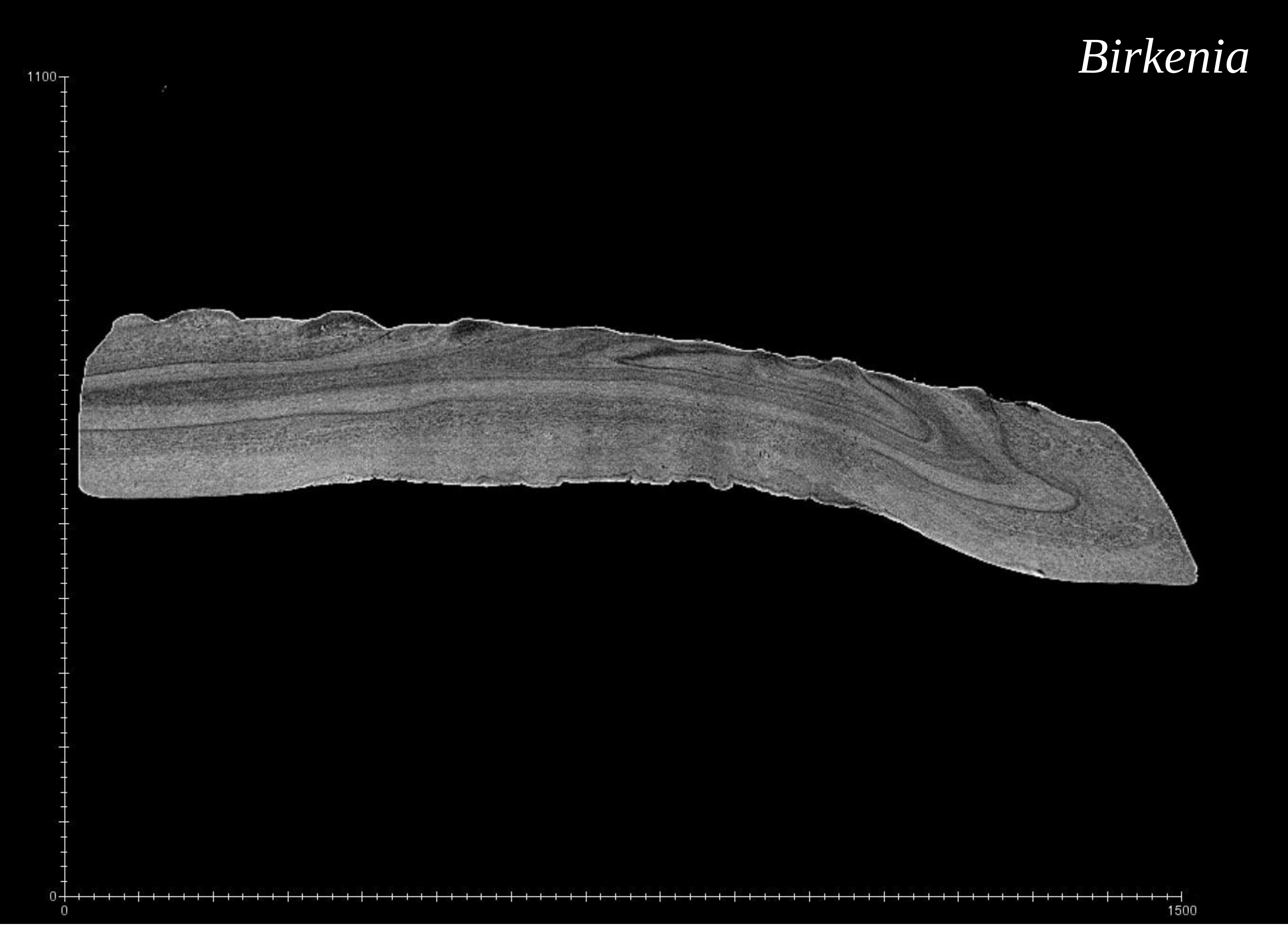

Fig. 19. Specimen NHMUK PV P73702. SRXTM longitudinal slice through a body scale of *Birkenia.* Data collected using 10X objective. Scale [illegible]

Birkenia

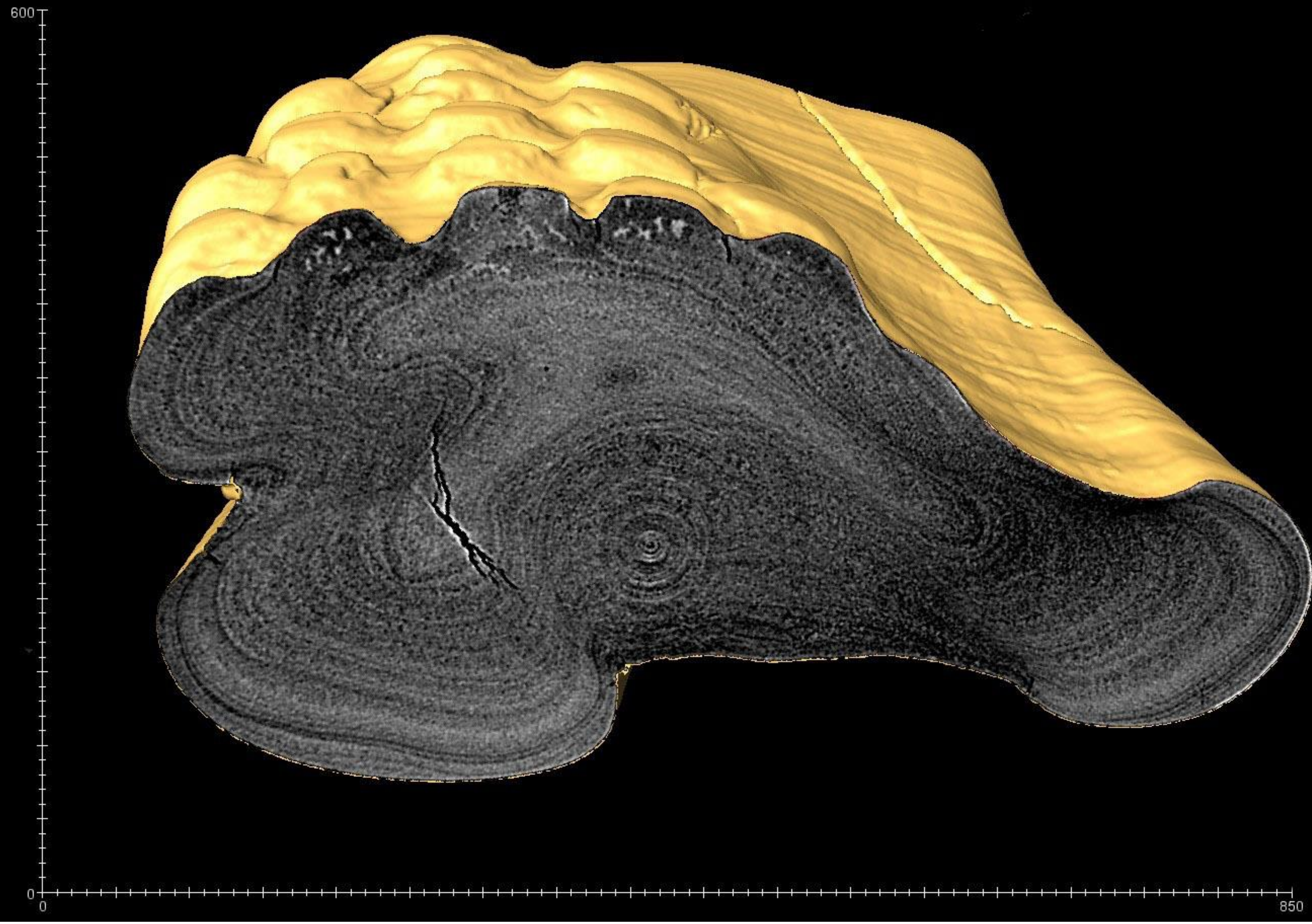

Fig. 20. Specimen NHMUK PV P73703. SRXTM slice through a body scale of *Birkenia*. Data collected using 10X objective. Scale bars in μm.

Rhyncholepis

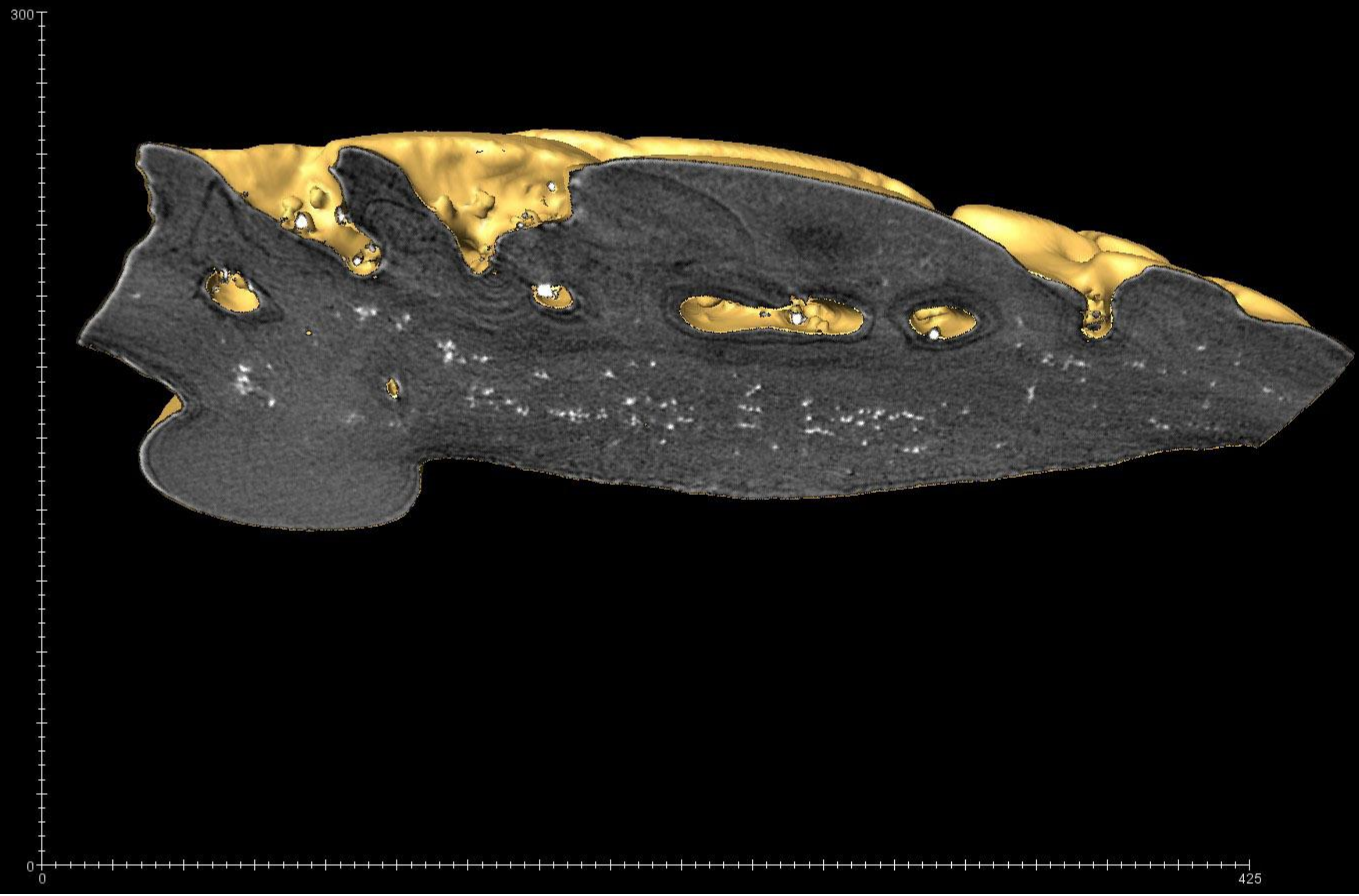

Fig. 21. Specimen NHMUK PV P73704. SRXTM slice through a body scale of *Rhyncholepis.* Data collected using 10X objective. Scale bars in

Rhyncholepis

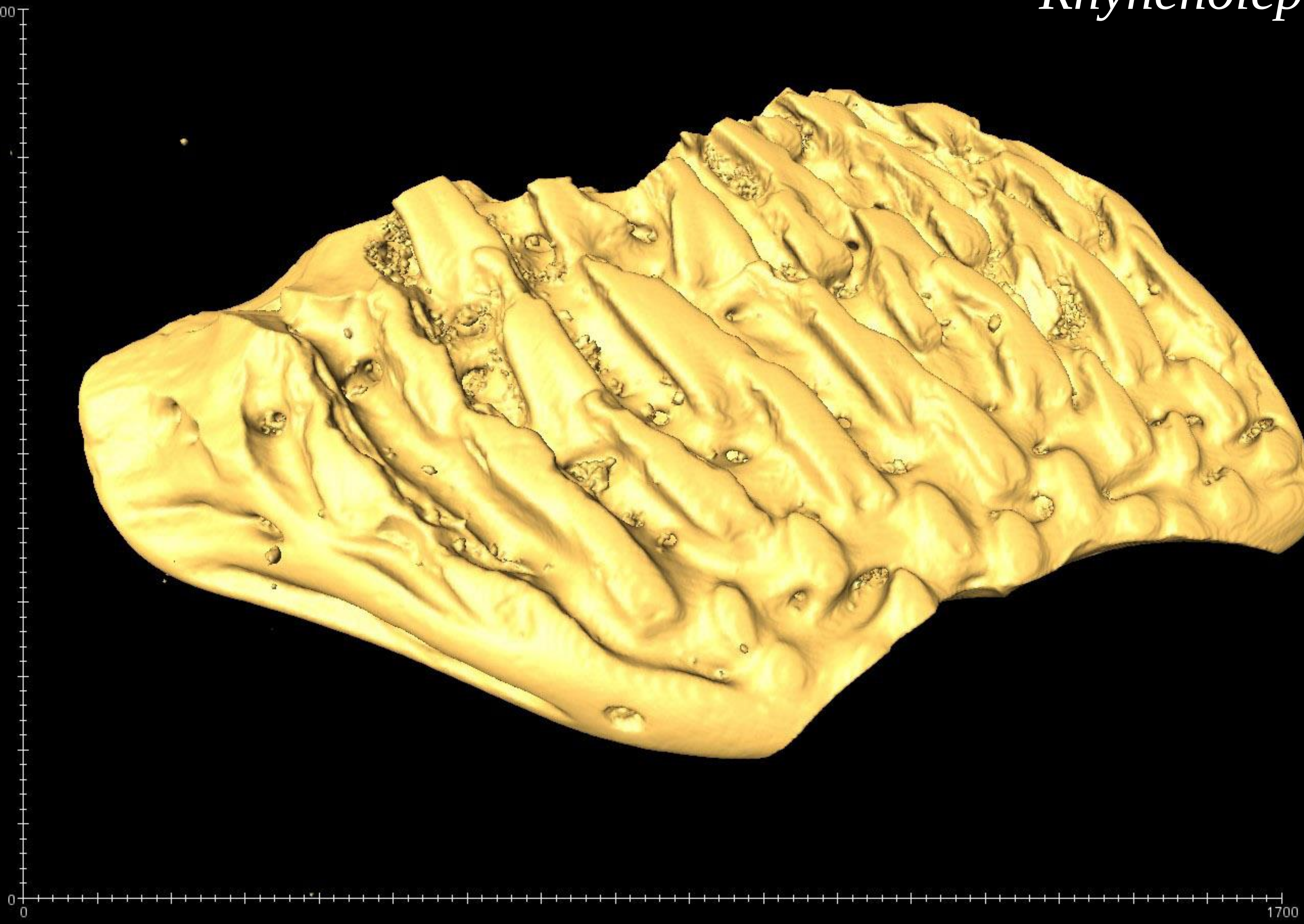

Fig. 22. Specimen NHMUK PV P73704. SRXTM isosurface of a *Rhyncholepis* body scale Note the pores between the tubercles, which open into the pore canal network. Data collected using 10X objective. Scale bars in μm.

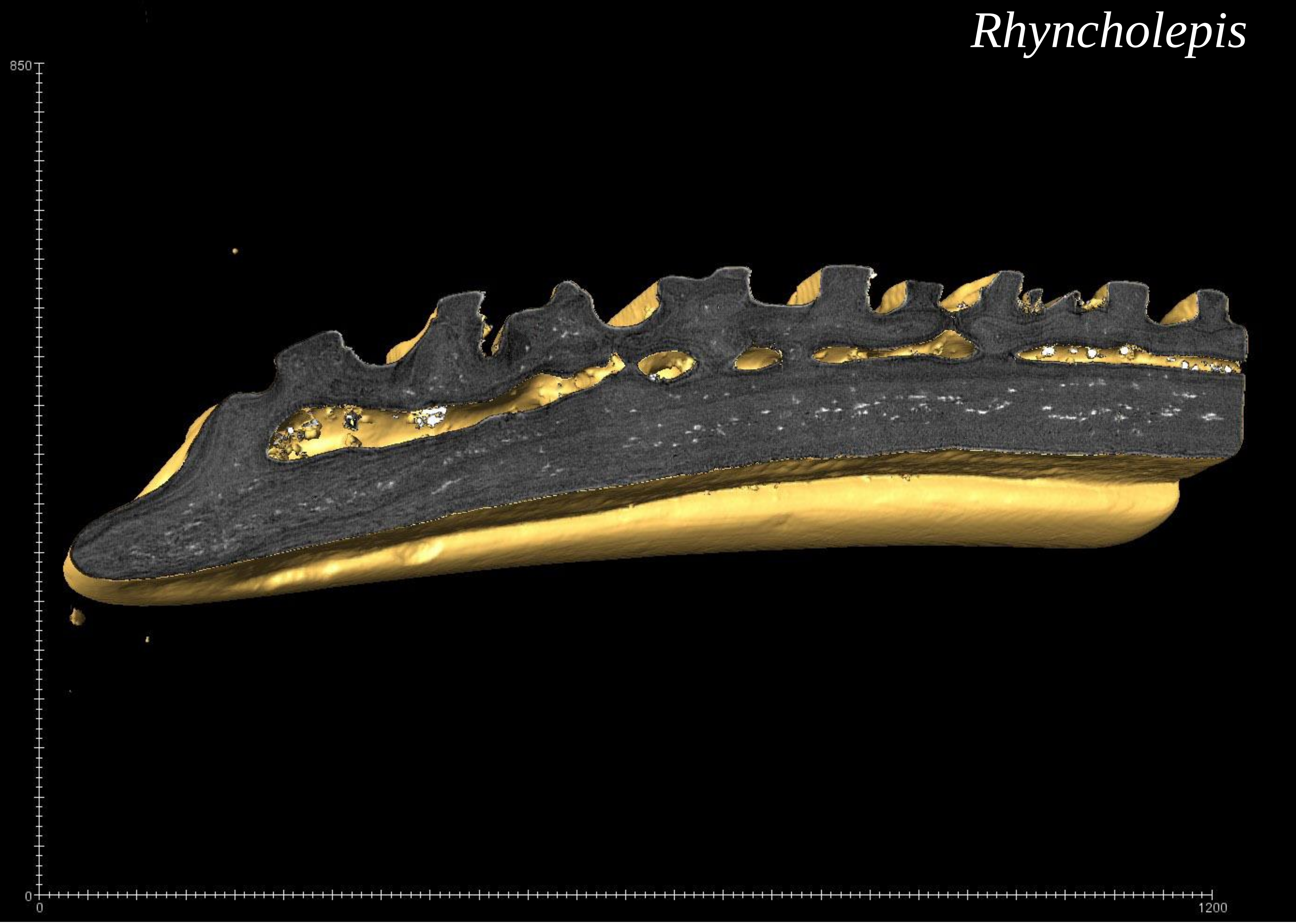

Fig. 23. Specimen NHMUK PV P73704. SRXTM longitudinal slice of a *Rhyncholepis* body scale Data collected using 20X objective. Scale bars in

Rhyncholepis

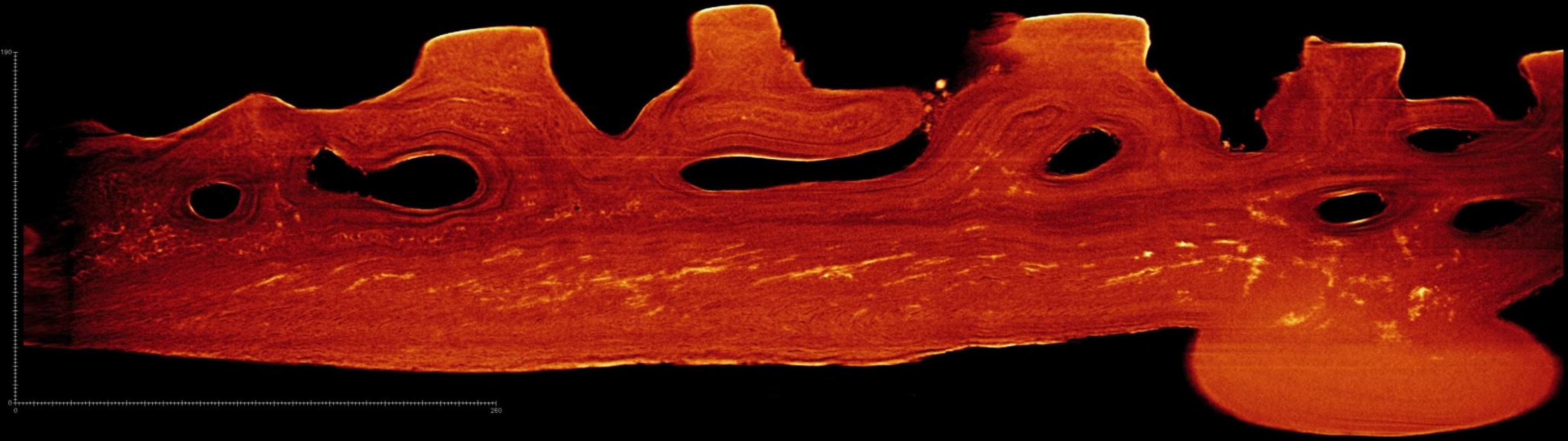

Fig. 24. Specimen NHMUK PV P73704. SRXTM longitudinal volume rendered thin section of a *Rhyncholepis* body scale Data collected using 10X objective. Scale bars in μm.

Rhyncholepis

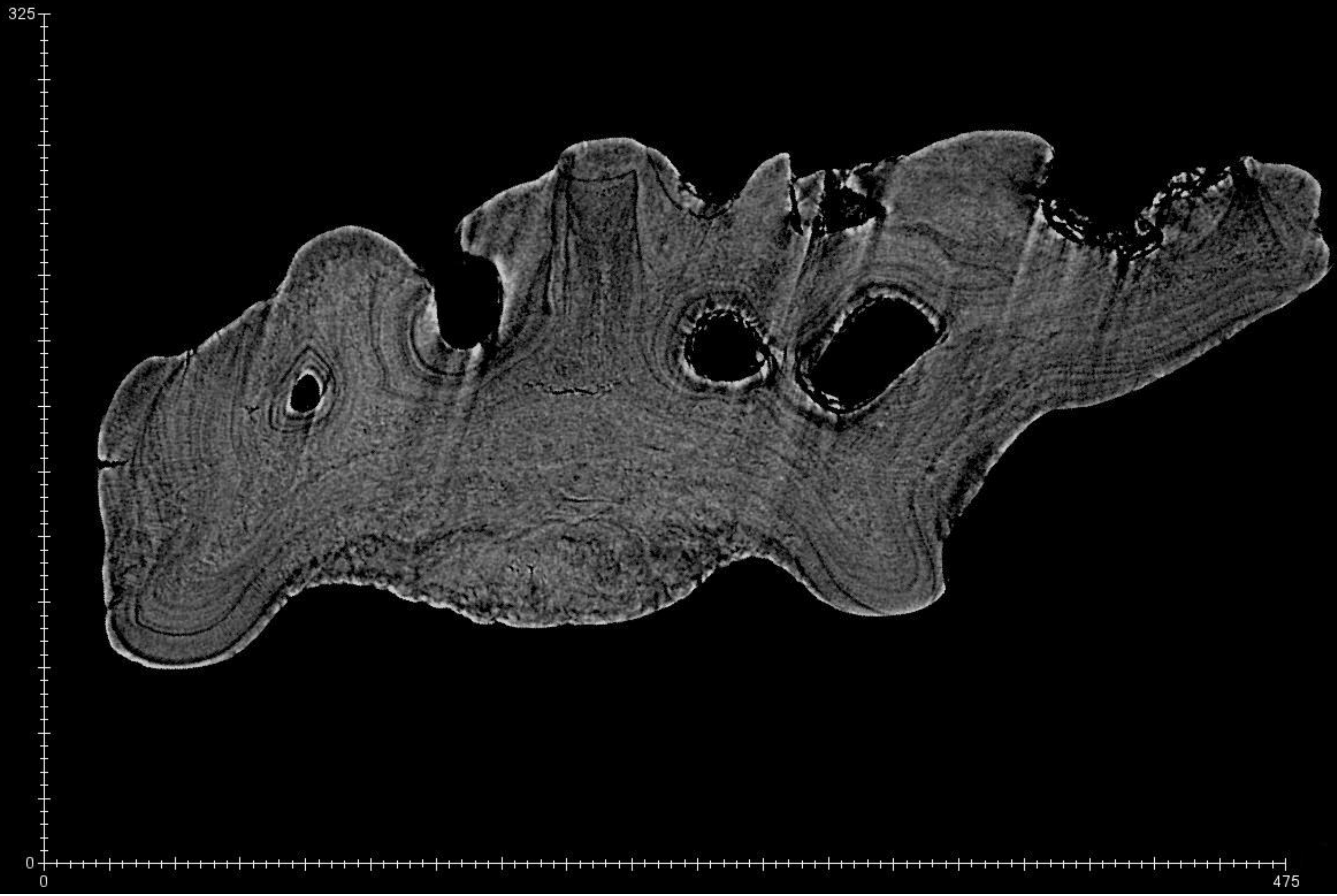

Fig. 25. Specimen NHMUK PV P73705. SRXTM slice of a *Rhyncholepis* body scale Data collected using 20X objective. Scale bars in μm.

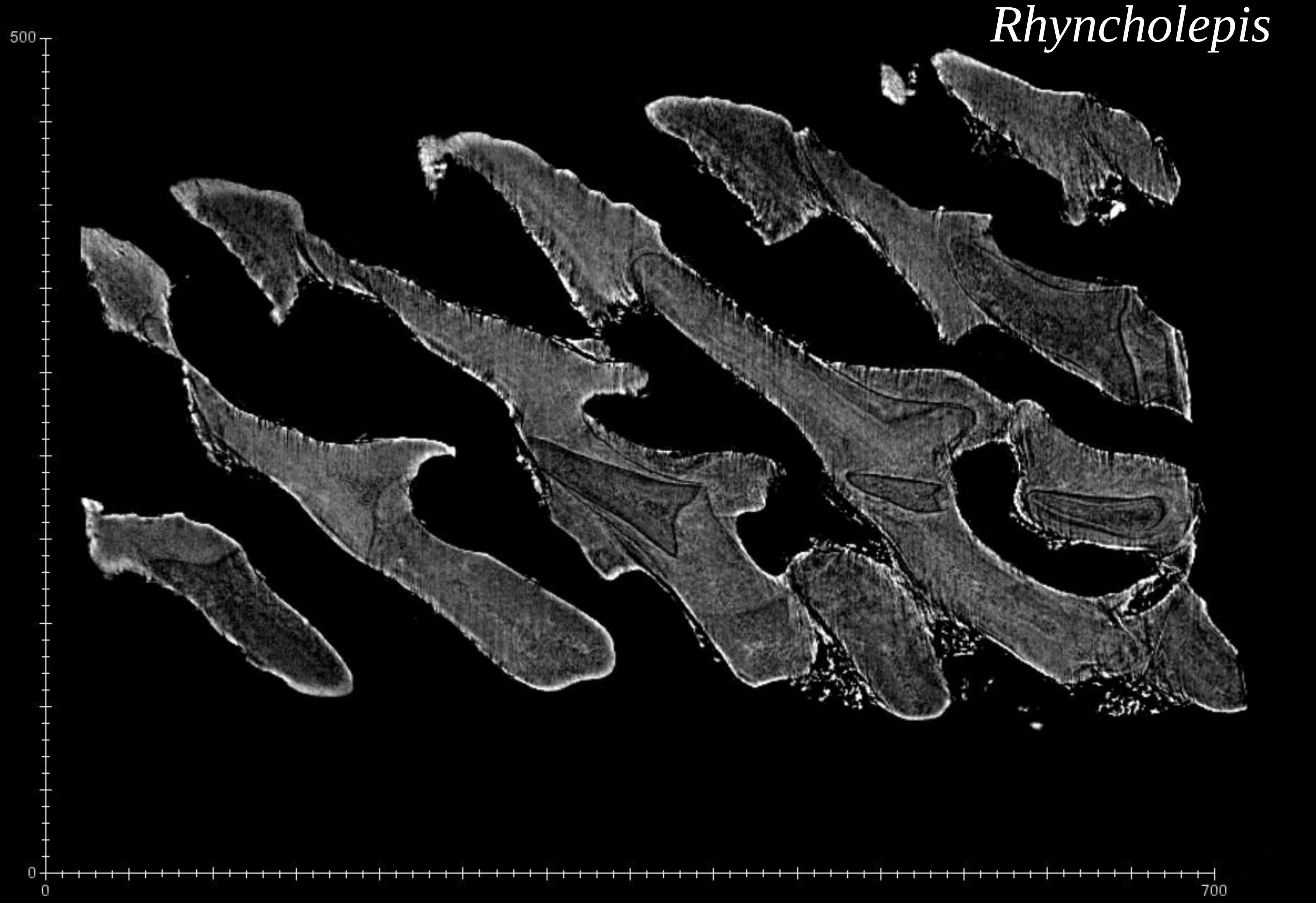

Fig. 26. Specimen NHMUK PV P73705. SRXTM horizontal slice through the superficial layer of a *Rhyncholepis* body scale Data collected using 20X objective. Scale bars in µm.

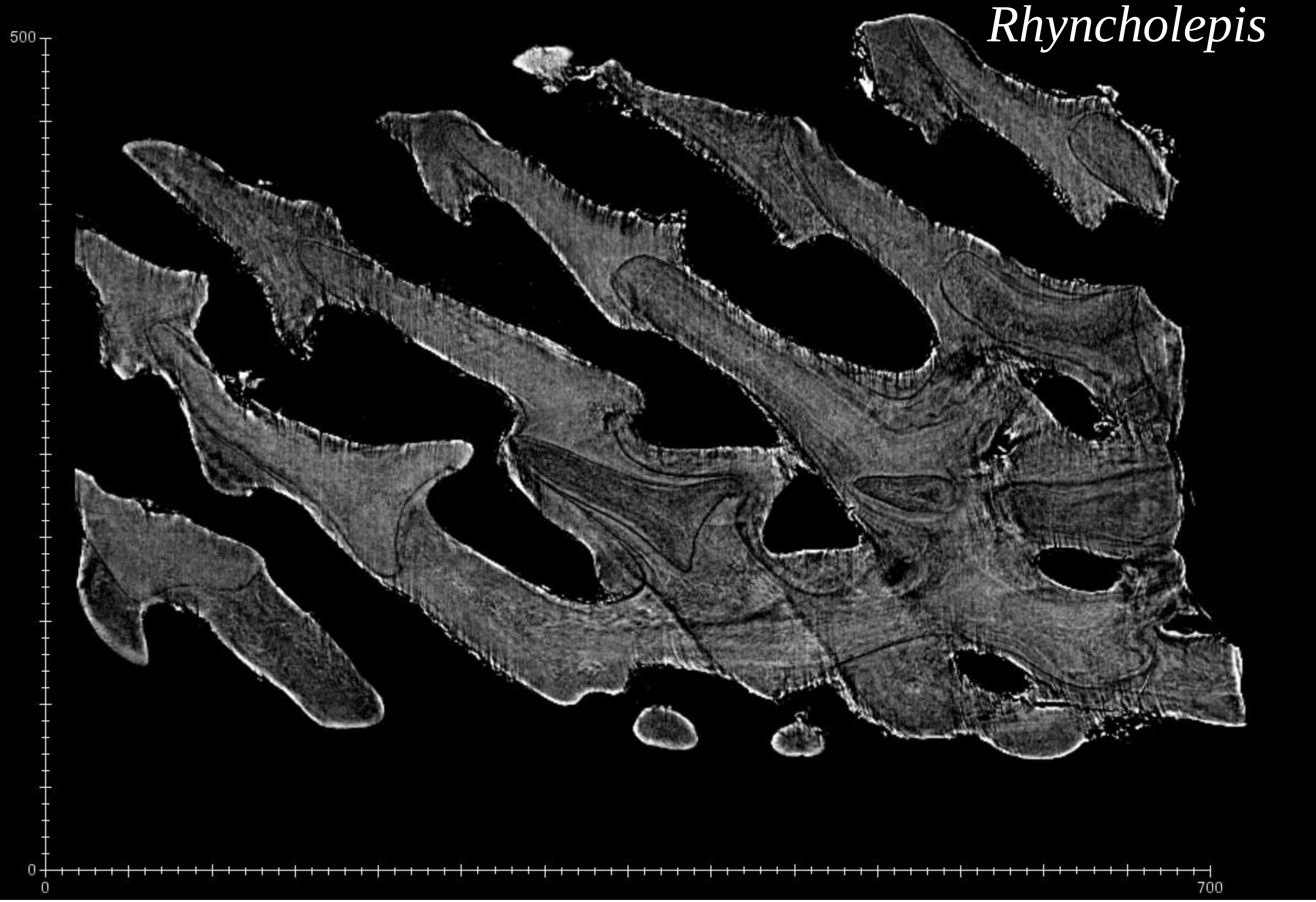

Fig. 27. Specimen NHMUK PV P73705. SRXTM horizontal slice through the superficial layer of a *Rhyncholepis* body scale Data collected using 20X objective. Scale bars in µm.

Rhyncholepis

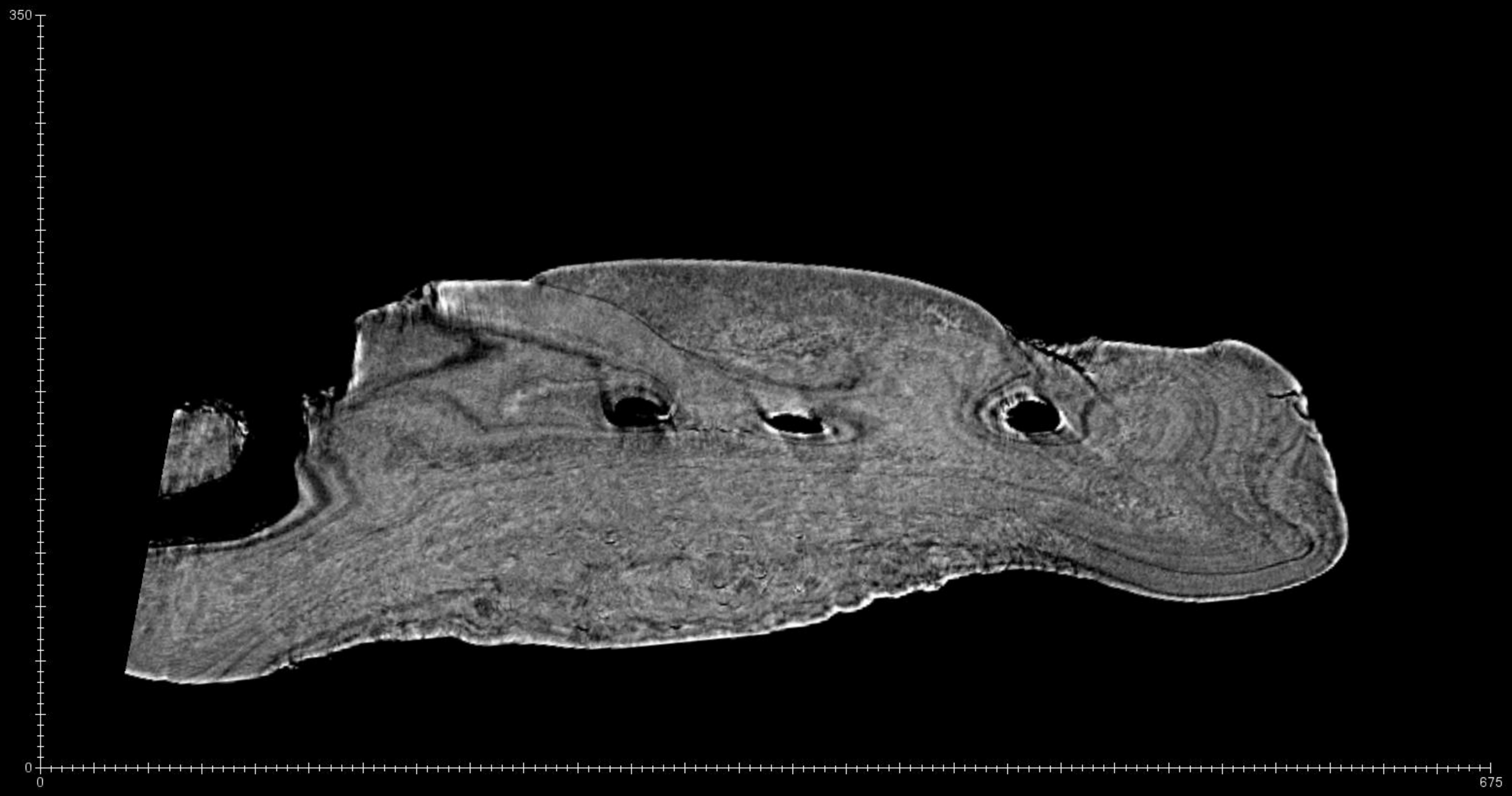

Fig. 28. Specimen NHMUK PV P73705. SRXTM slice of a *Rhyncholepis* body scale Data collected using 20X objective. Scale bars in µm.

Rhyncholepis

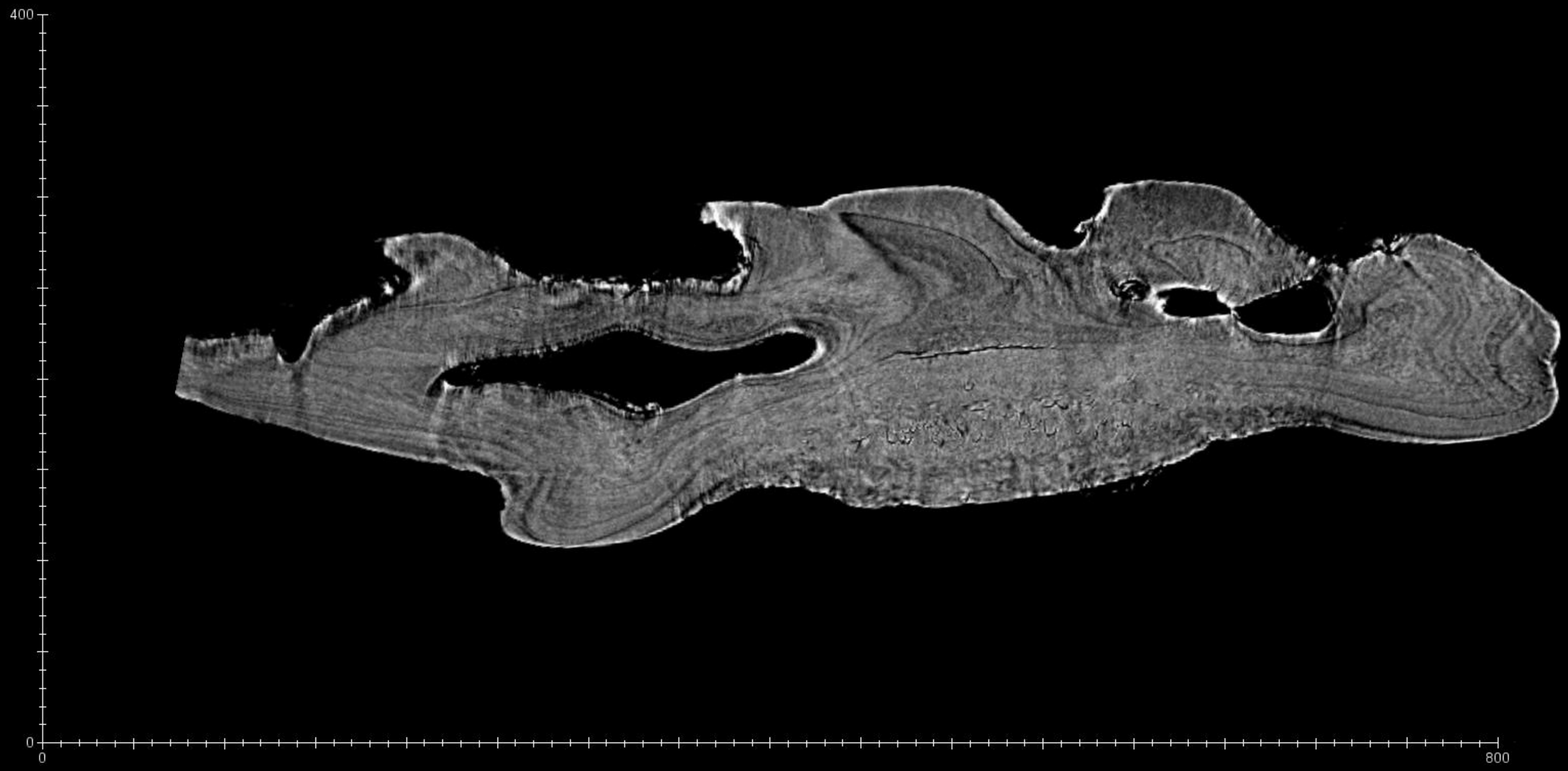

Fig. 29. Specimen NHMUK PV P73705. SRXTM slice of a *Rhyncholepis* body scale Data collected using 20X objective. Scale bars in μm.

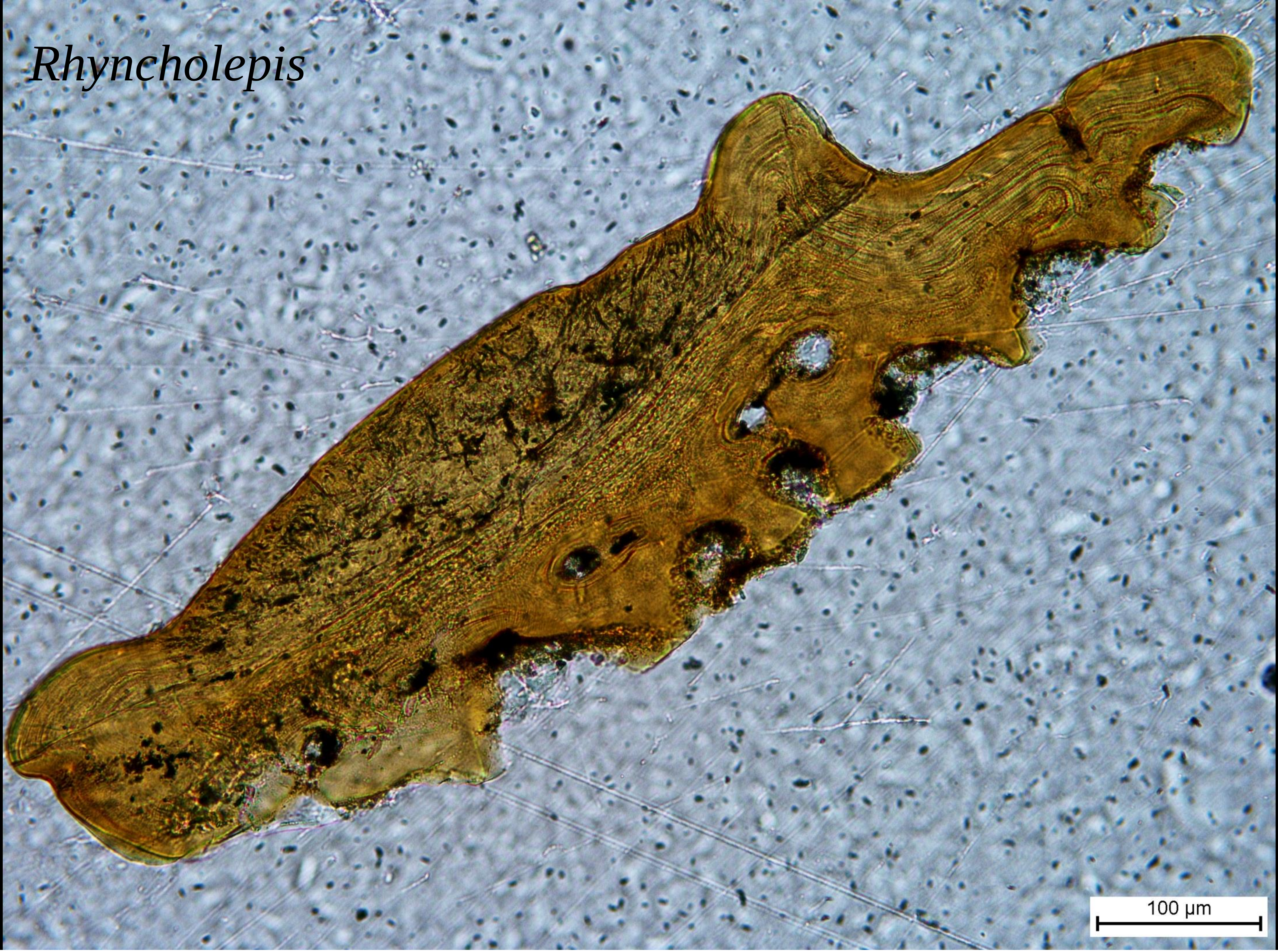

Fig. 30. Specimen NHMUK PV P73706. LM thin section of a *Rhyncholepis* body scale.

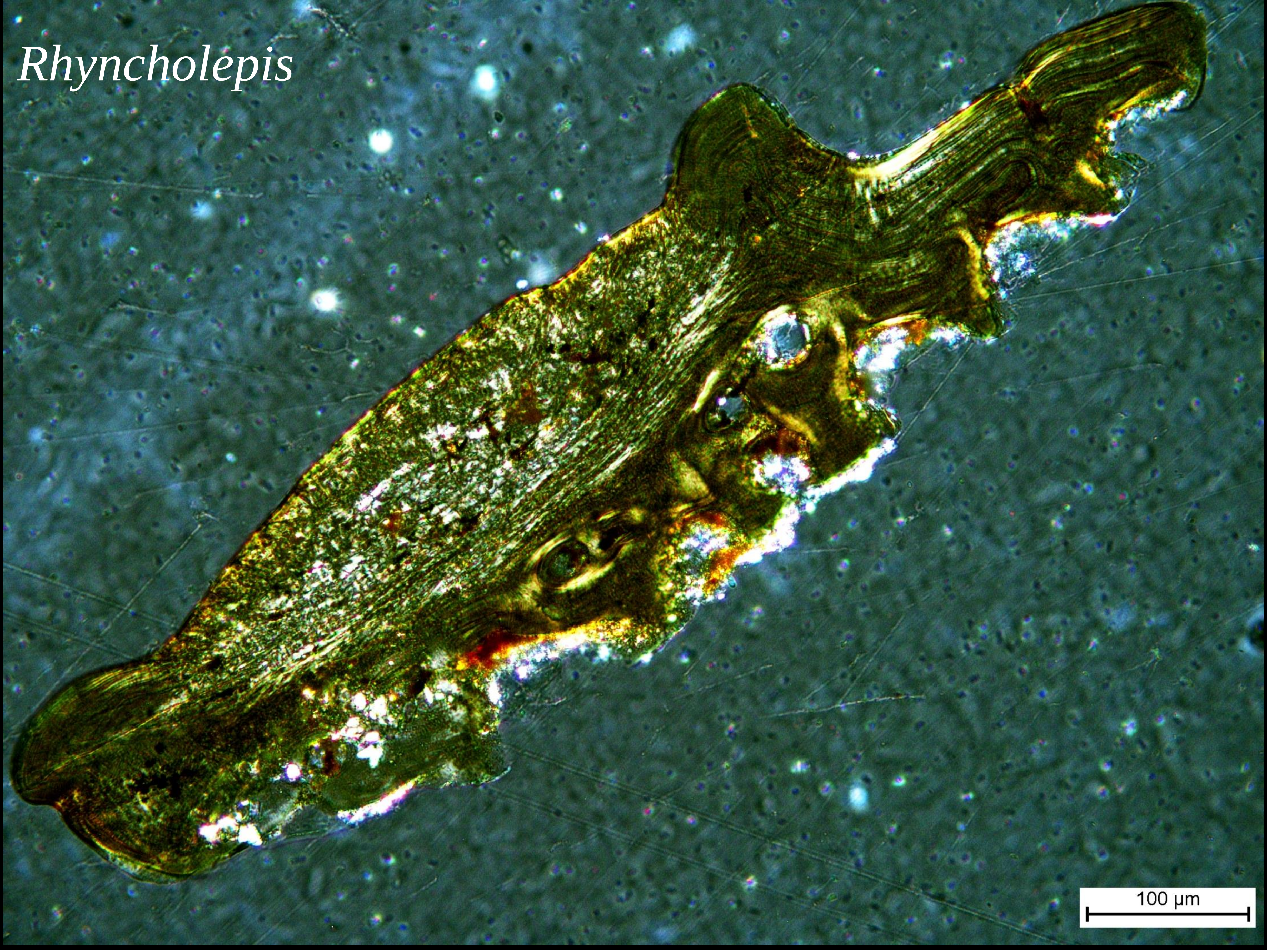

Fig. 31. Specimen NHMUK PV P73706. LM thin section of a *Rhyncholepis* body scale under cross-polarized light.

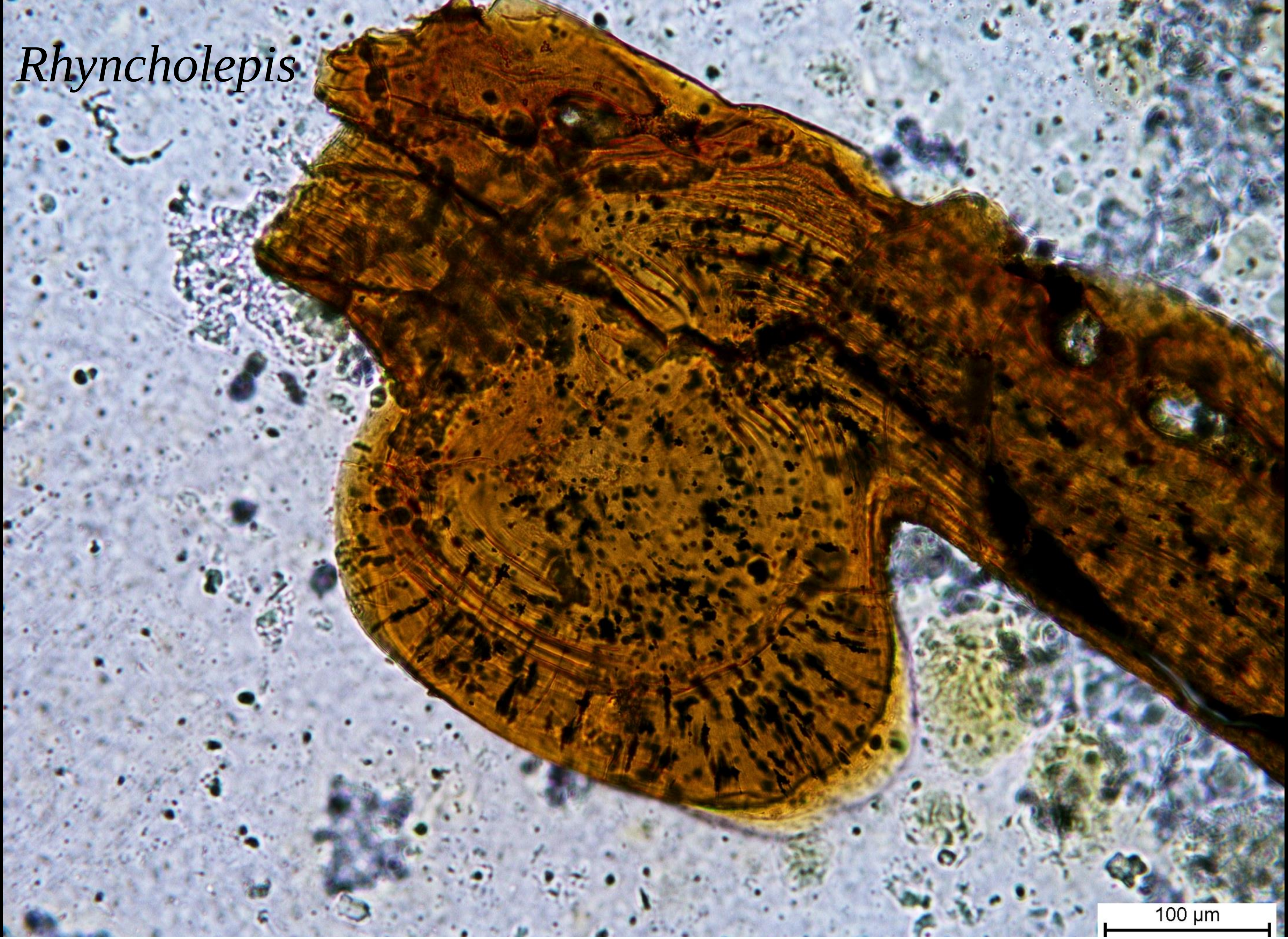

Fig. 32. Specimen NHMUK PV P73707. LM thin section of the mendian visceral rib of *Rhyncholepis*. Note the radial thread-like spaces in filled with pyrite.

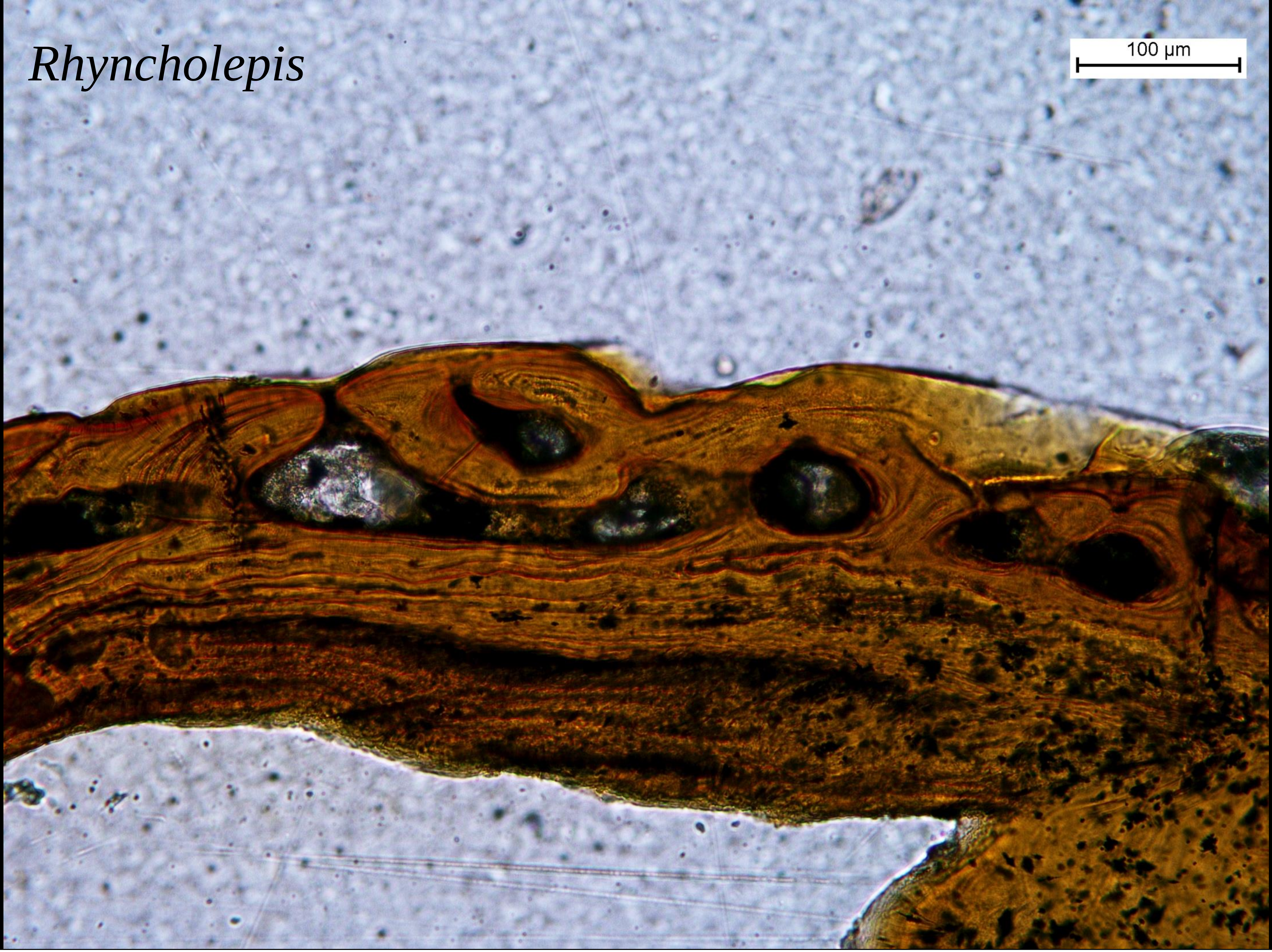

Fig. 33. Specimen NHMUK PV P73707. LM thin section of a *Rhyncholepis* body scale.

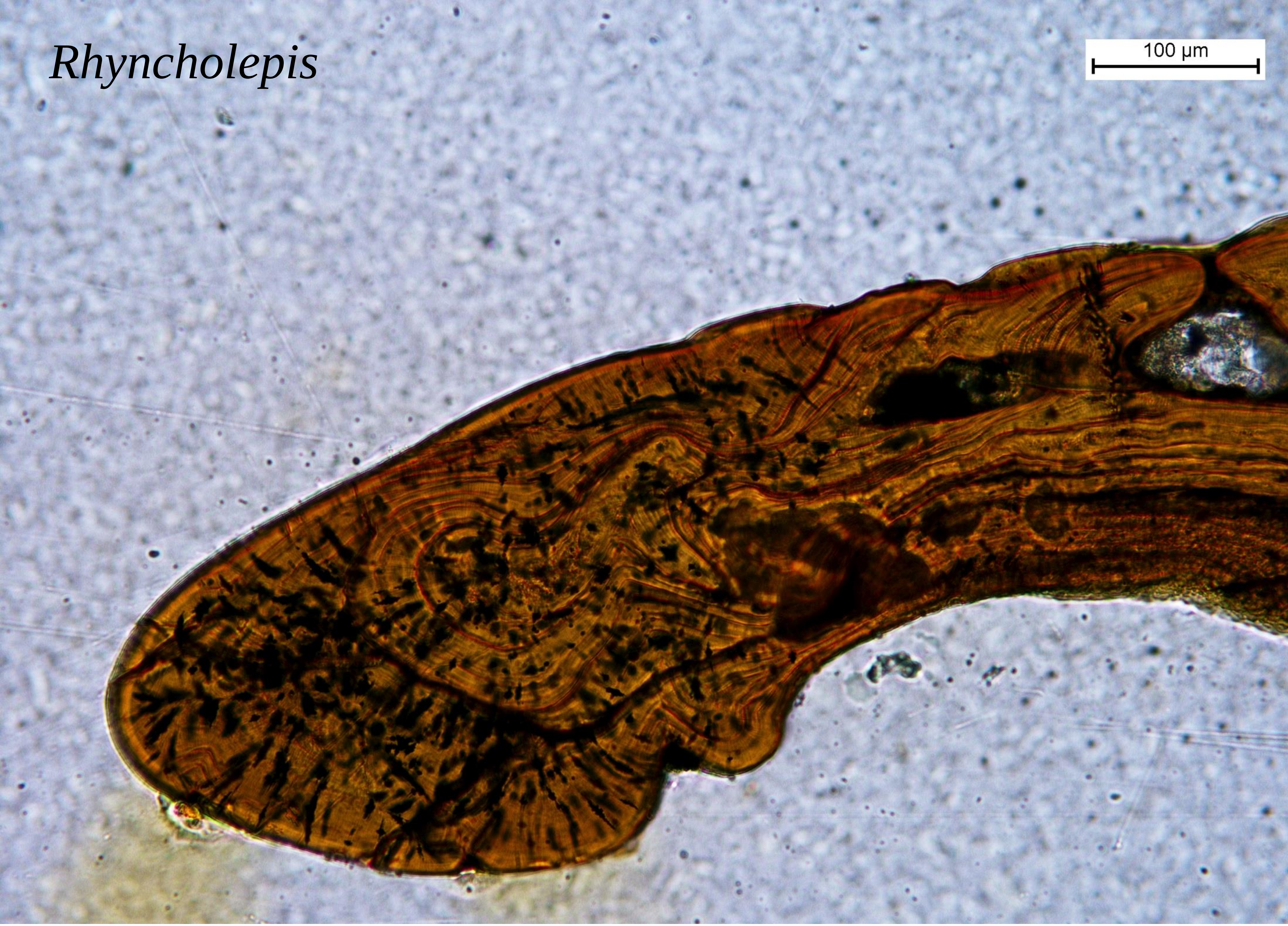

Fig. 34. Specimen NHMUK PV P73707. LM thin section of the anterior visceral rib of a *Rhyncholepis* body scale.

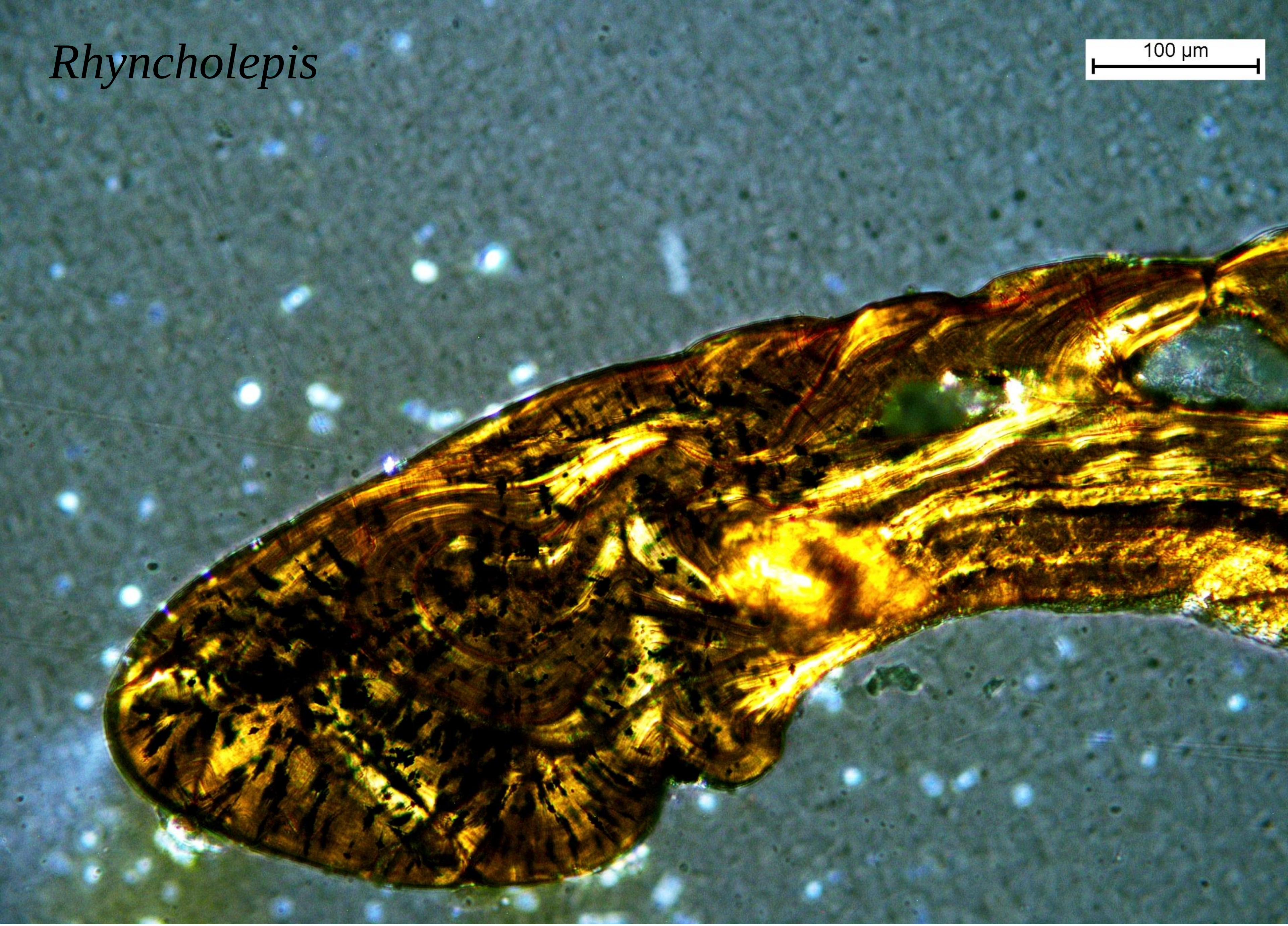

Fig. 35. Specimen NHMUK PV P73707. LM thin section of the anterior visceral rib of a *Rhyncholepis* body scale under cross polarized light.

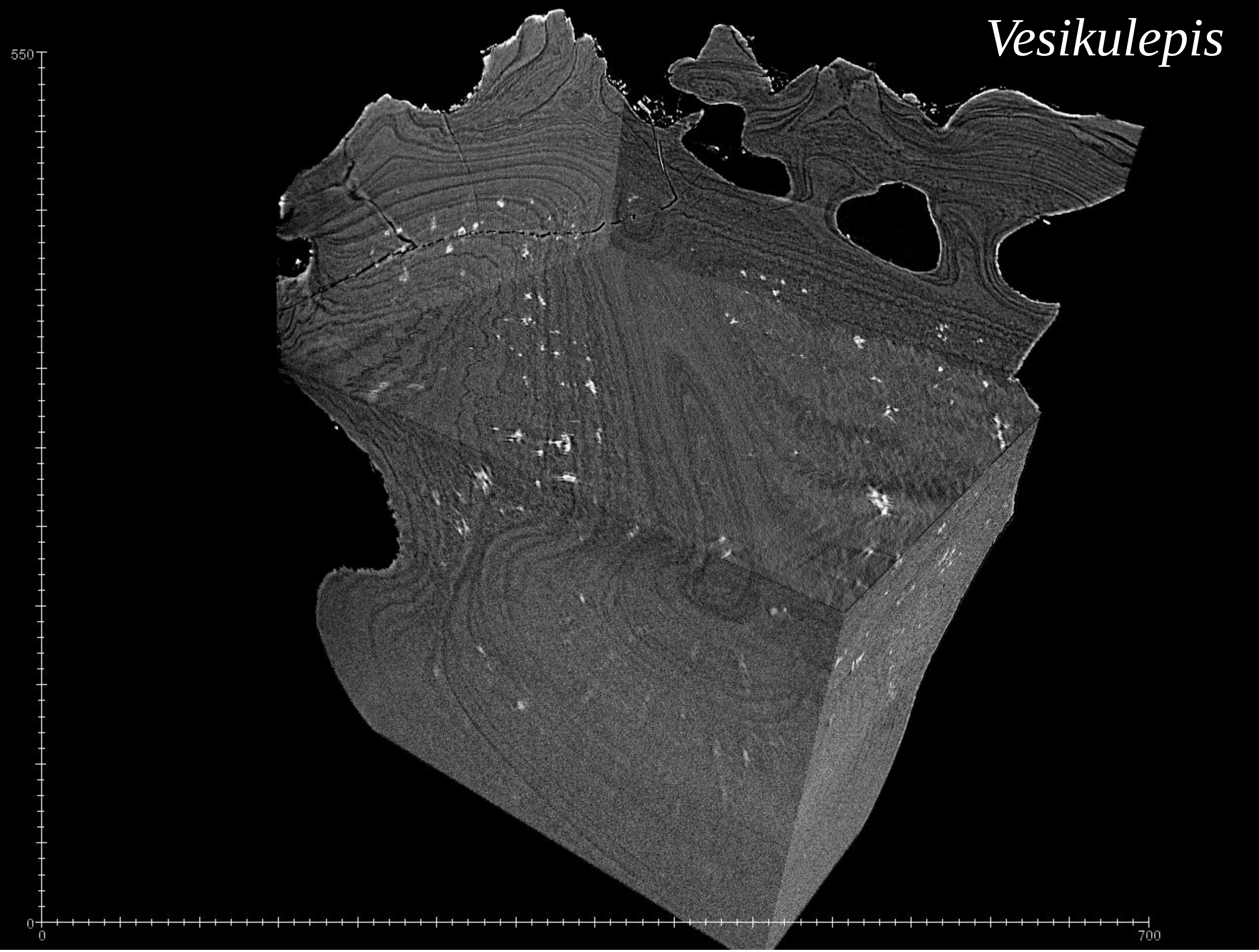

Fig. 36. Specimen NHMUK PV P73708. Block model of SRXTM slices through a body scale of *Vesikulepis*. Data collected using 40X objective. Scale bars in μm.

Vesikulepis

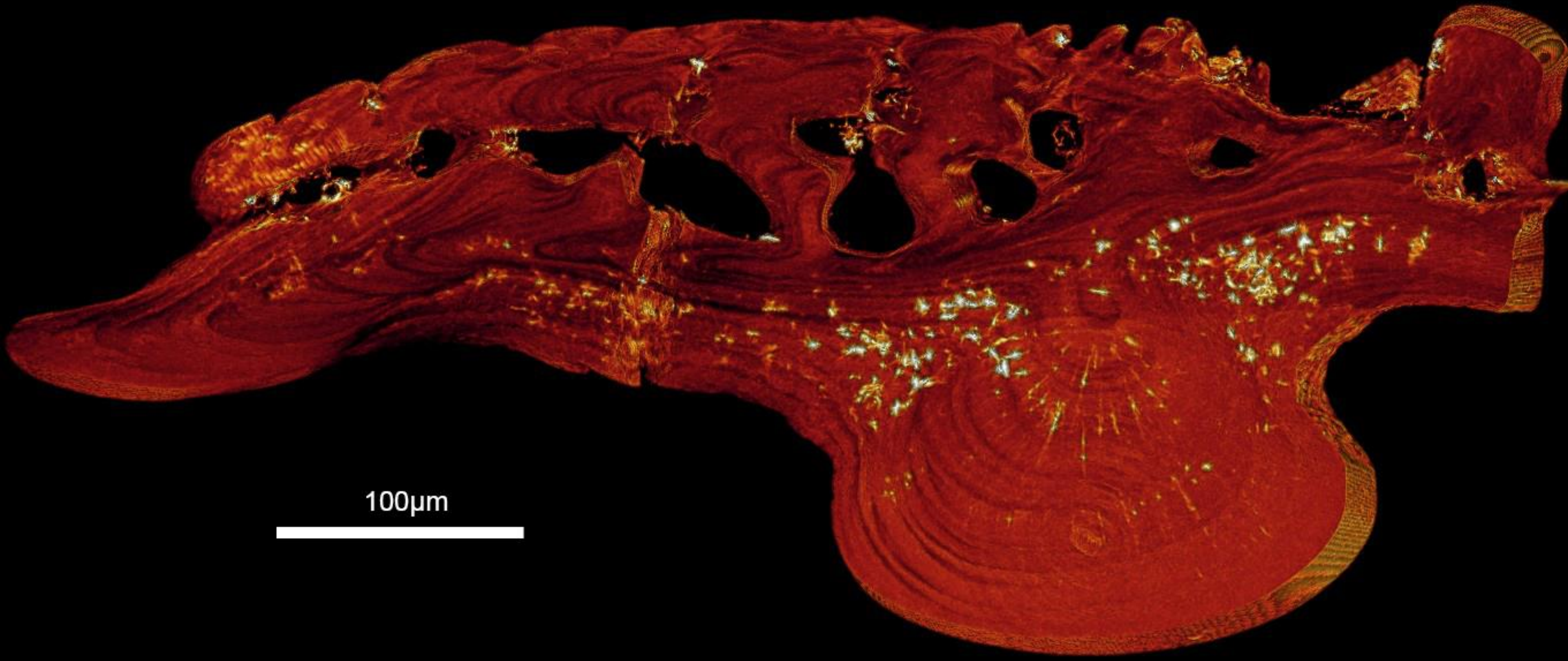

Fig. 37. Specimen NHMUK PV P73708. SRXTM volume rendered virtual thin section of a body scale of *Vesikulepis*. Data collected using 10X objective. Scale bars in µm.

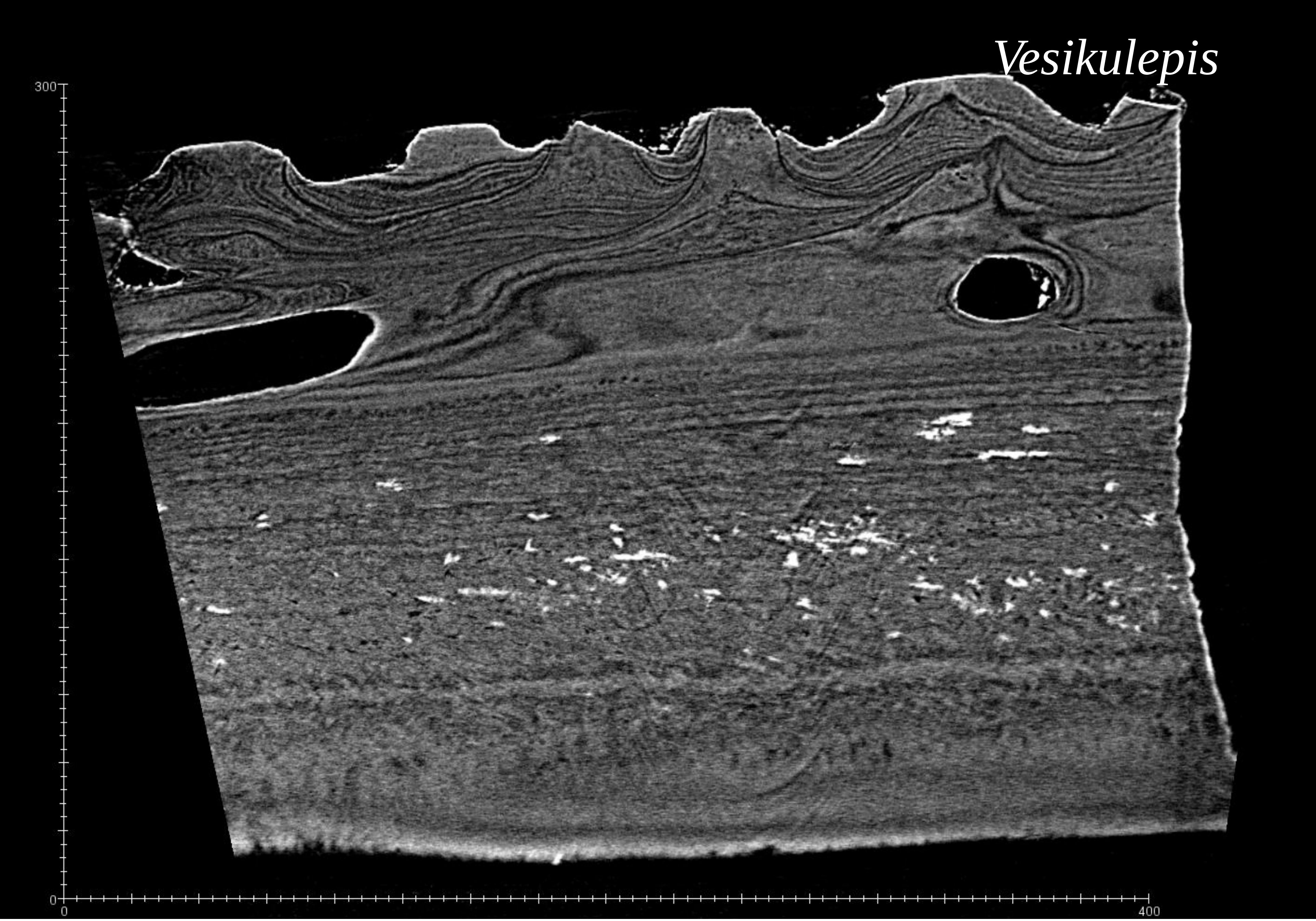

Fig. 38. Specimen NHMUK PV P73708. SRXTM longitudinal slice through the superficial layer and median visceral rib of a *Vesikulepis* body scale. Data collected using 40X objective. Scale bars in µm.

Vesikulepis

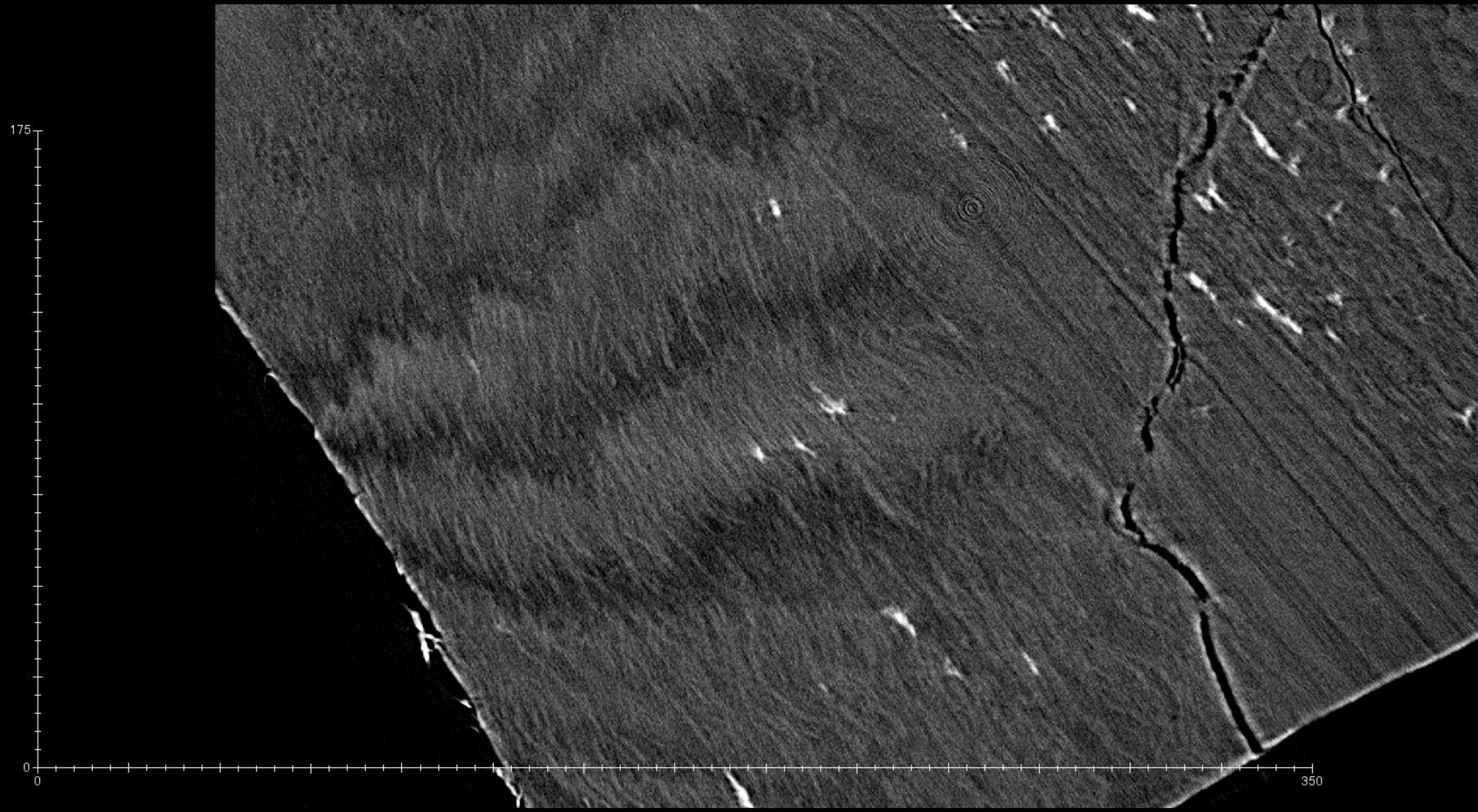

Fig. 39. Specimen NHMUK PV P73708. SRXTM horizontal slice through the basal layer of a *Vesikulepis* body scale. Data collected using 40X objective. Scale bars in μm.

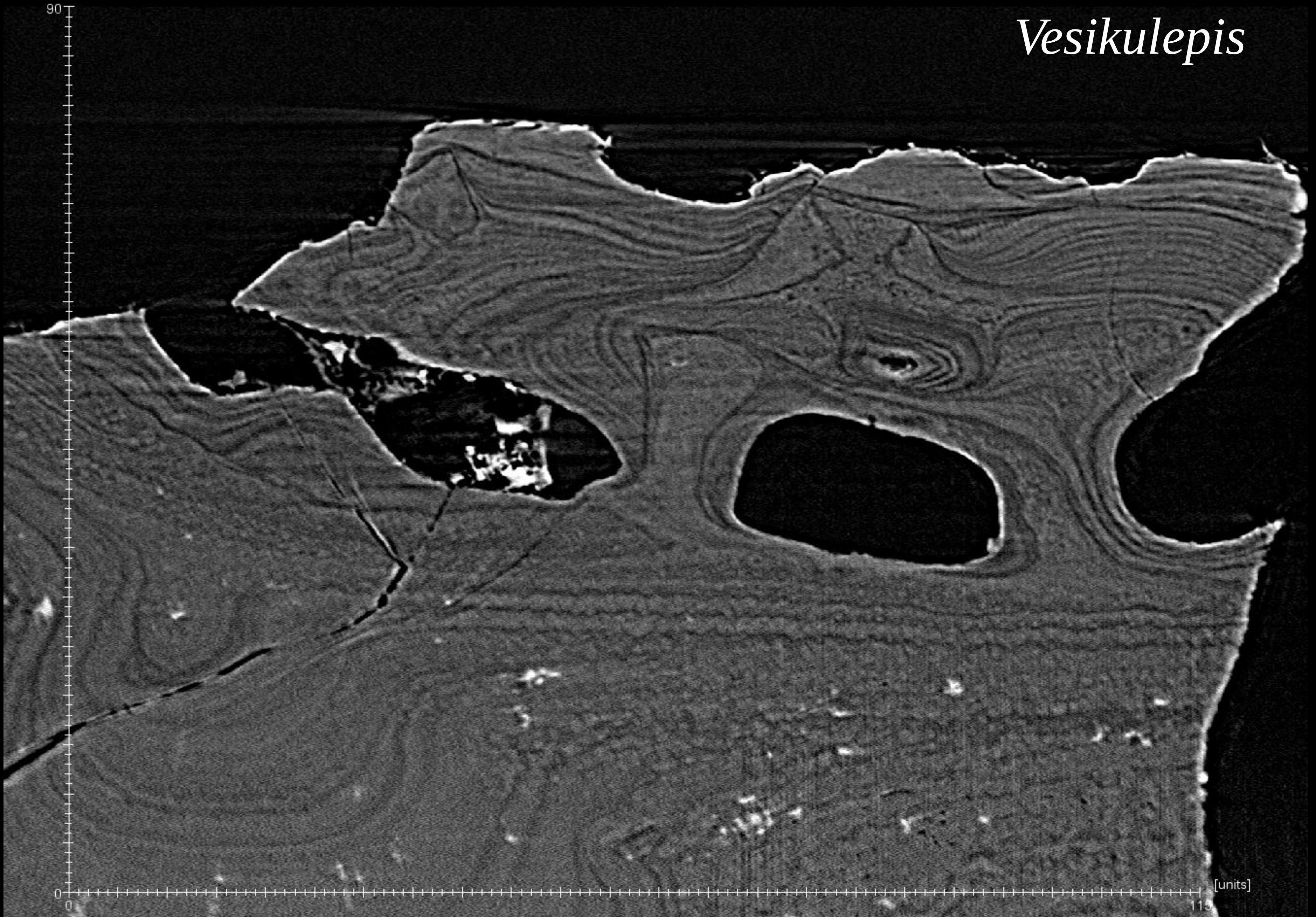

Fig. 40. Specimen NHMUK PV P73708. SRXTM slice through the superficial layer and canal network of a *Vesikulepis* body scale. Data collected using 40X objective. Scale bars in µm.

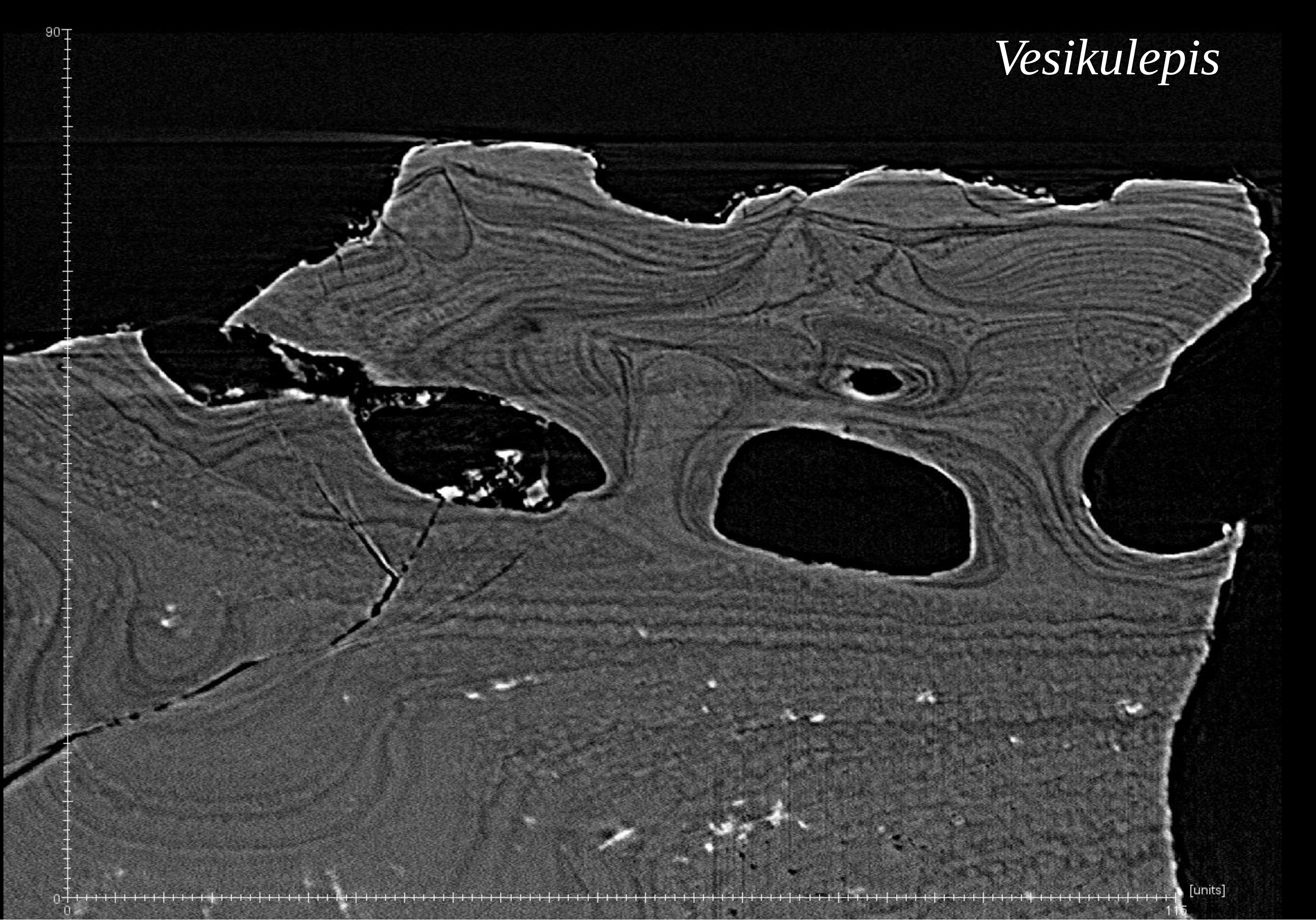

Fig. 41. Specimen NHMUK PV P73708. SRXTM slice through the superficial layer and canal network of a *Vesikulepis* body scale. Data collected using 40X objective. Scale bars in µm.

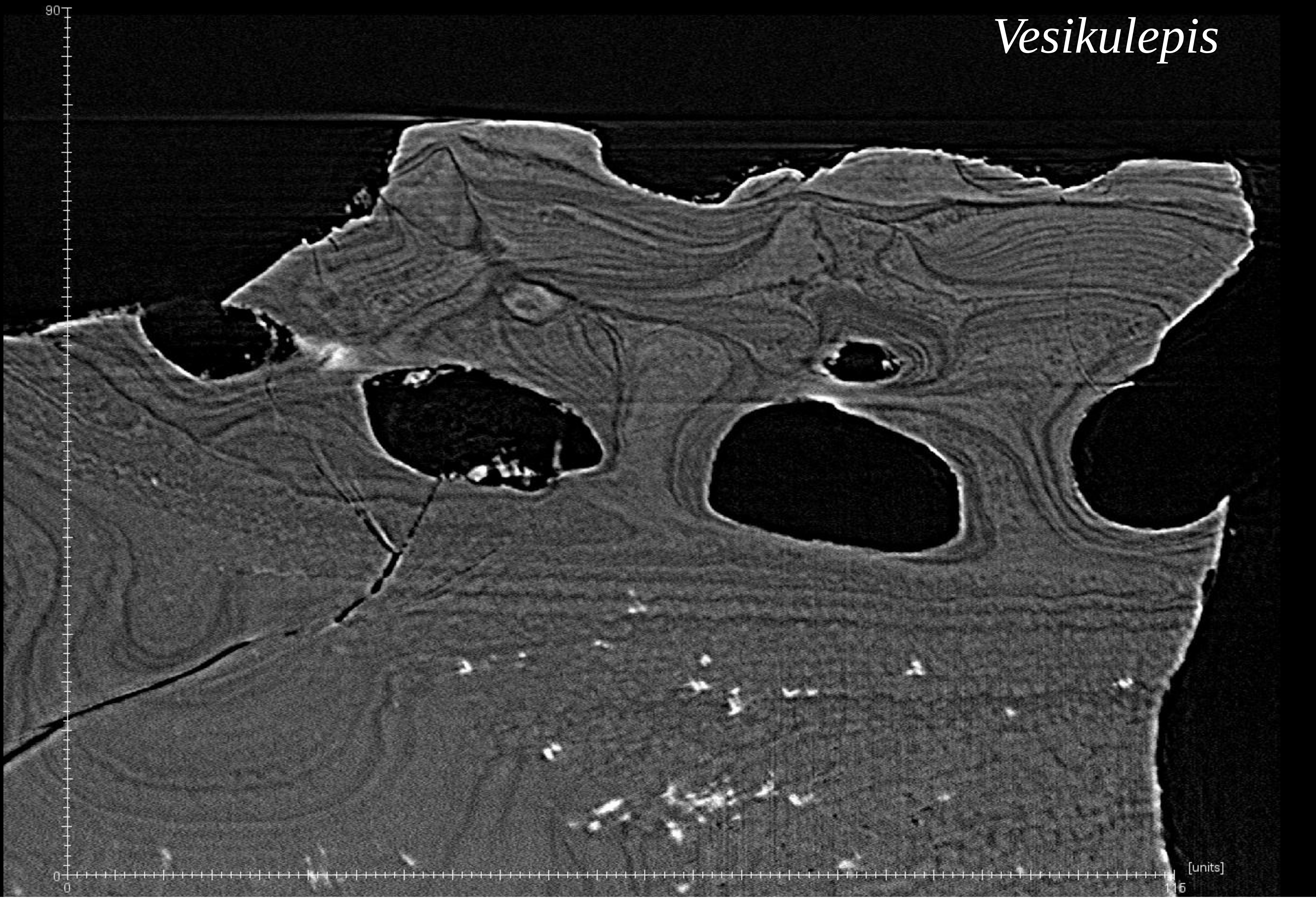

Fig. 42. Specimen NHMUK PV P73708. SRXTM slice through the superficial layer and canal network of a *Vesikulepis* body scale. Data collected using 40X objective. Scale bars in µm.

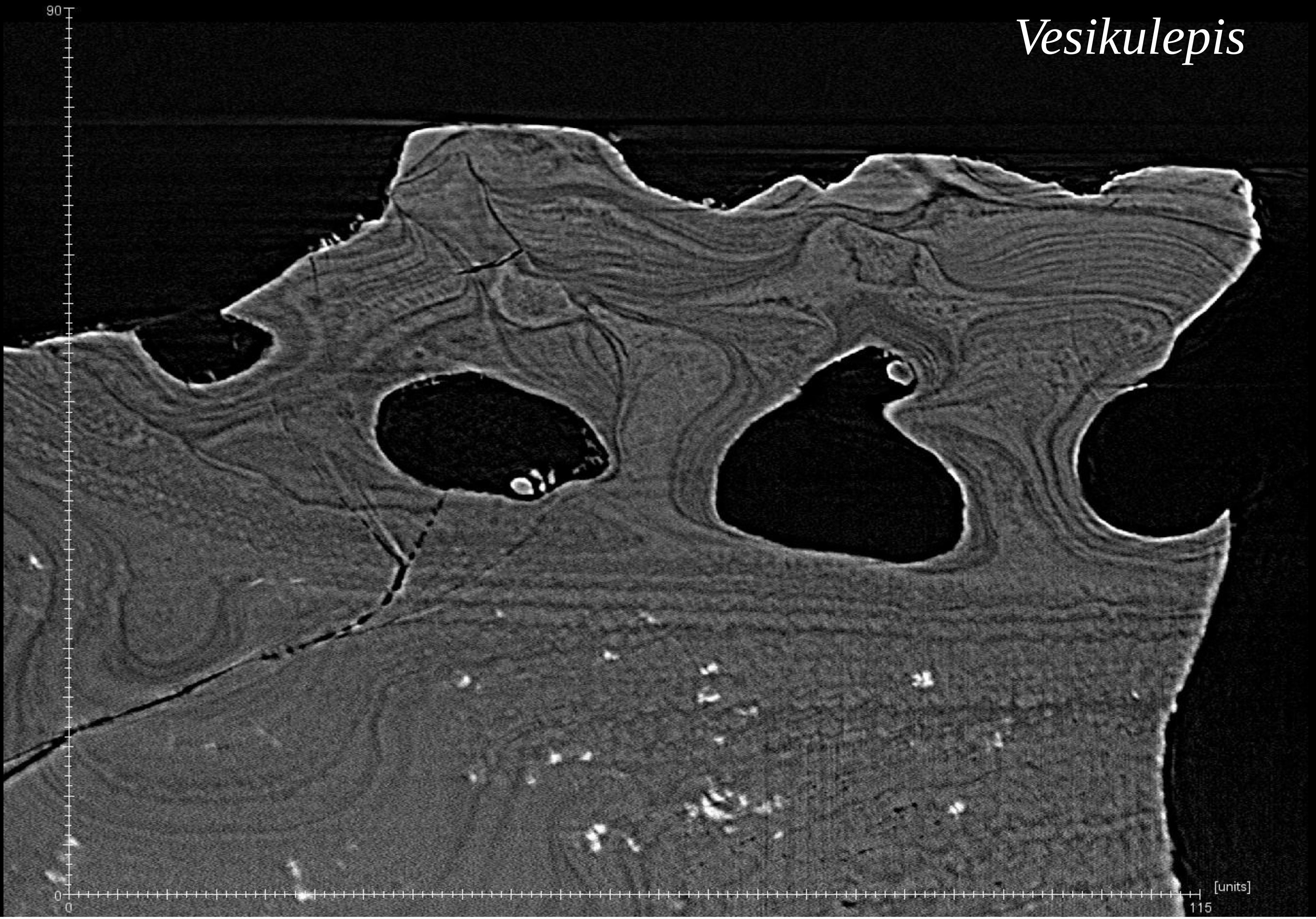

Fig. 43. Specimen NHMUK PV P73708. SRXTM slice through the superficial layer and canal network of a *Vesikulepis* body scale. Data collected using 40X objective. Scale bars in µm.

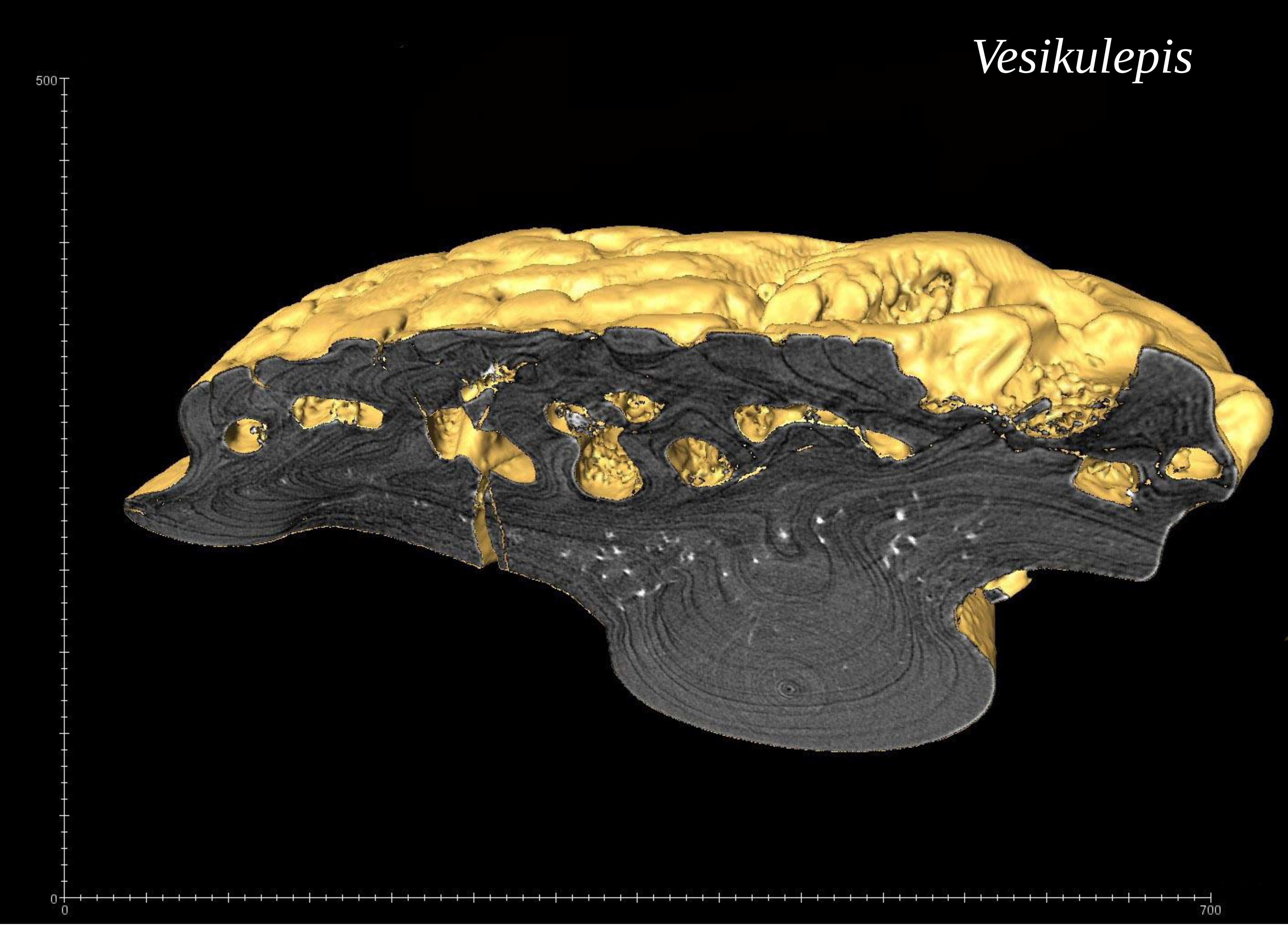

Fig. 44. Specimen NHMUK PV P73708. SRXTM slice through a *Vesikulepis* body scale. Data collected using 10X objective. Scale bars in µm.

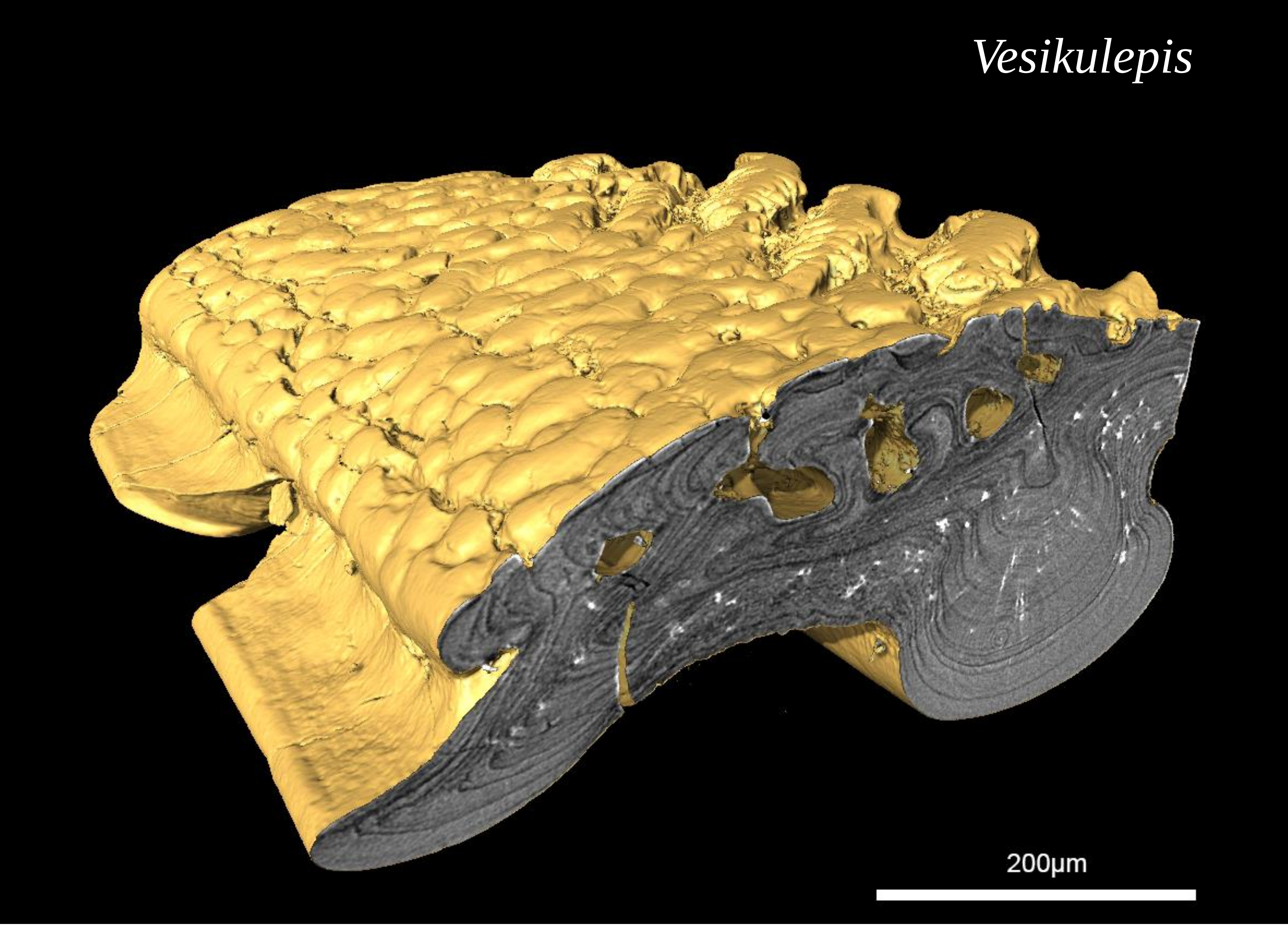

Fig. 45. Specimen NHMUK PV P73708. SRXTM slice through a *Vesikulepis* body scale. Data collected using 10X objective. Scale bars in µm.

Vesikulepis

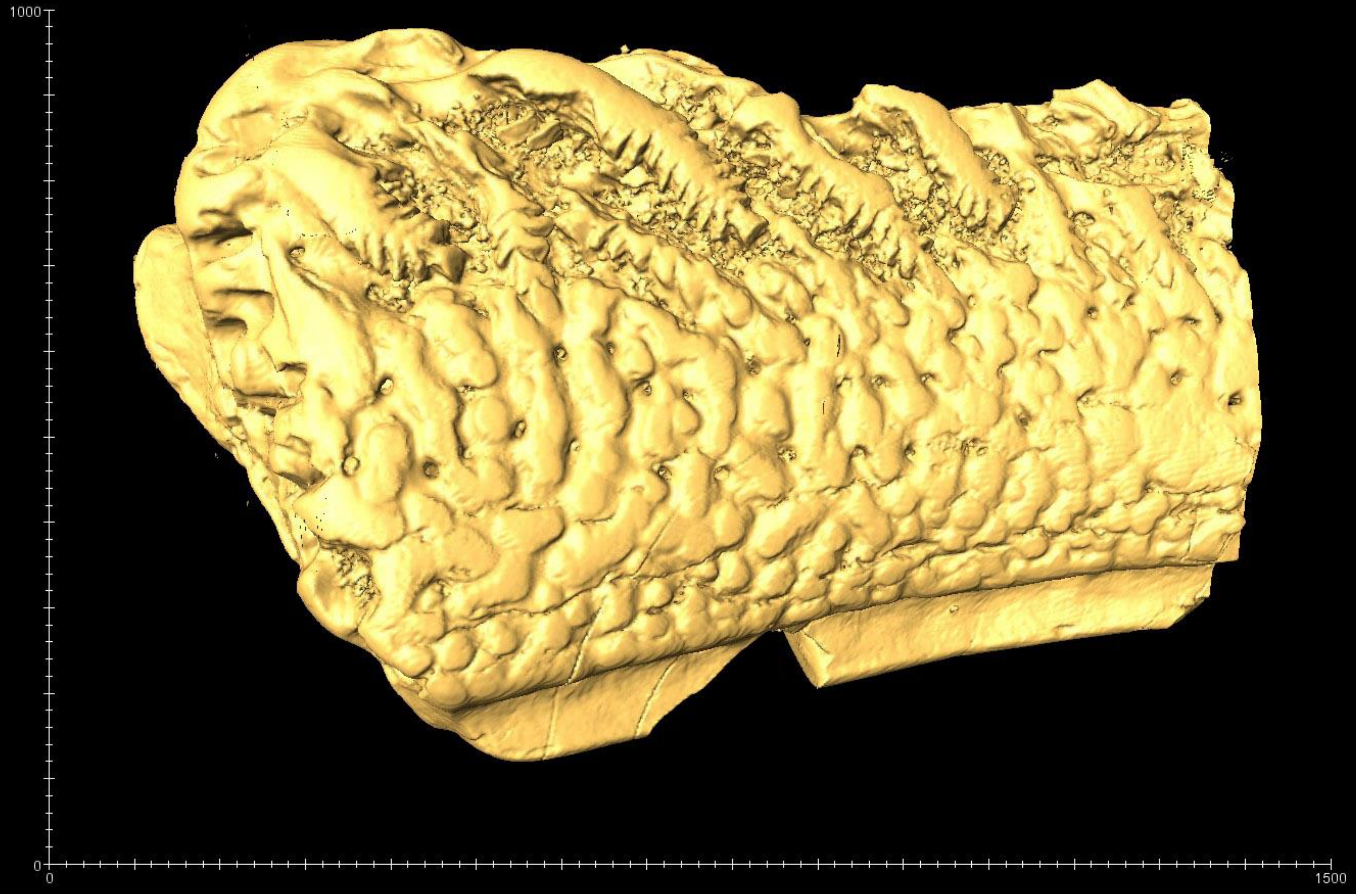

Fig. 46. Specimen NHMUK PV P73708. SRXTM isosurface of a *Vesikulepis* body scale. Data collected using 20X objective. Scale bars in μm.

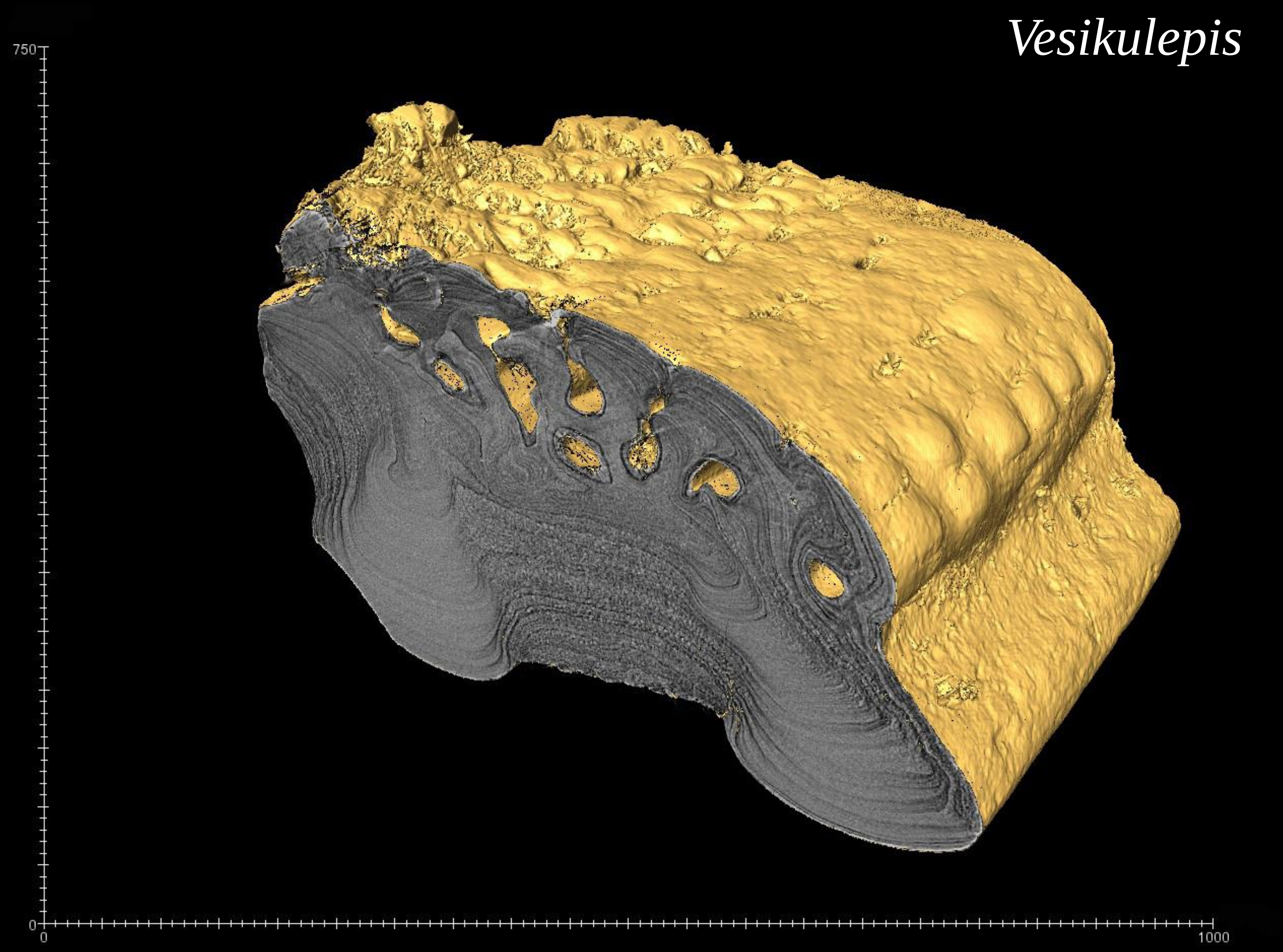

Fig. 47. Specimen NHMUK PV P73709. SRXTM slice through a *Vesikulepis* body scale. Data collected using 20X objective. Scale bars in µm.

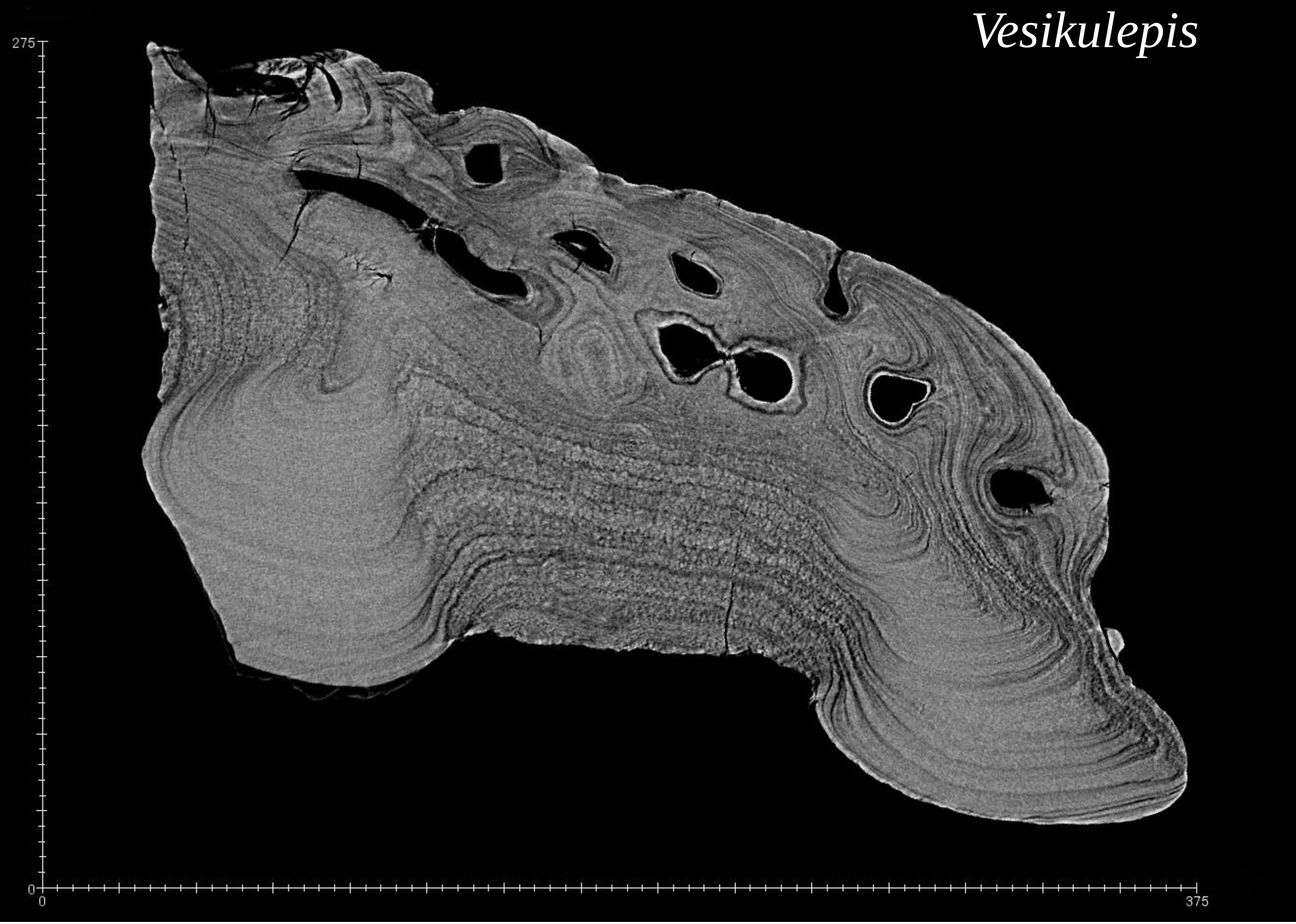

Fig. 48. Specimen NHMUK PV P73709. SRXTM slice through a *Vesikulepis* body scale. Data collected using 20X objective. Scale bars in μm.

Vesikulepis

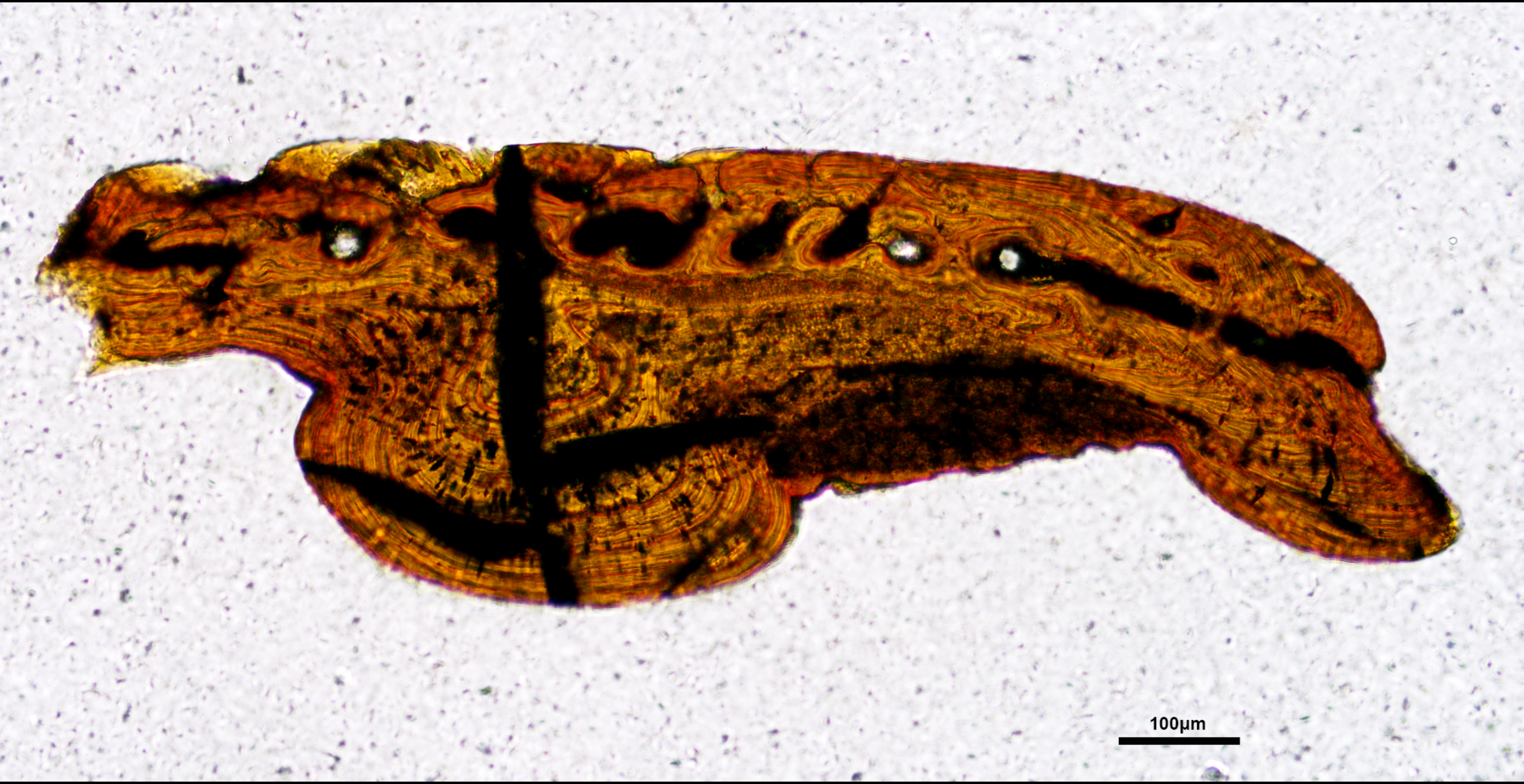

Fig. 49. Specimen NHMUK PV P73710. LM thin section of a *Vesikulepis* body scale..

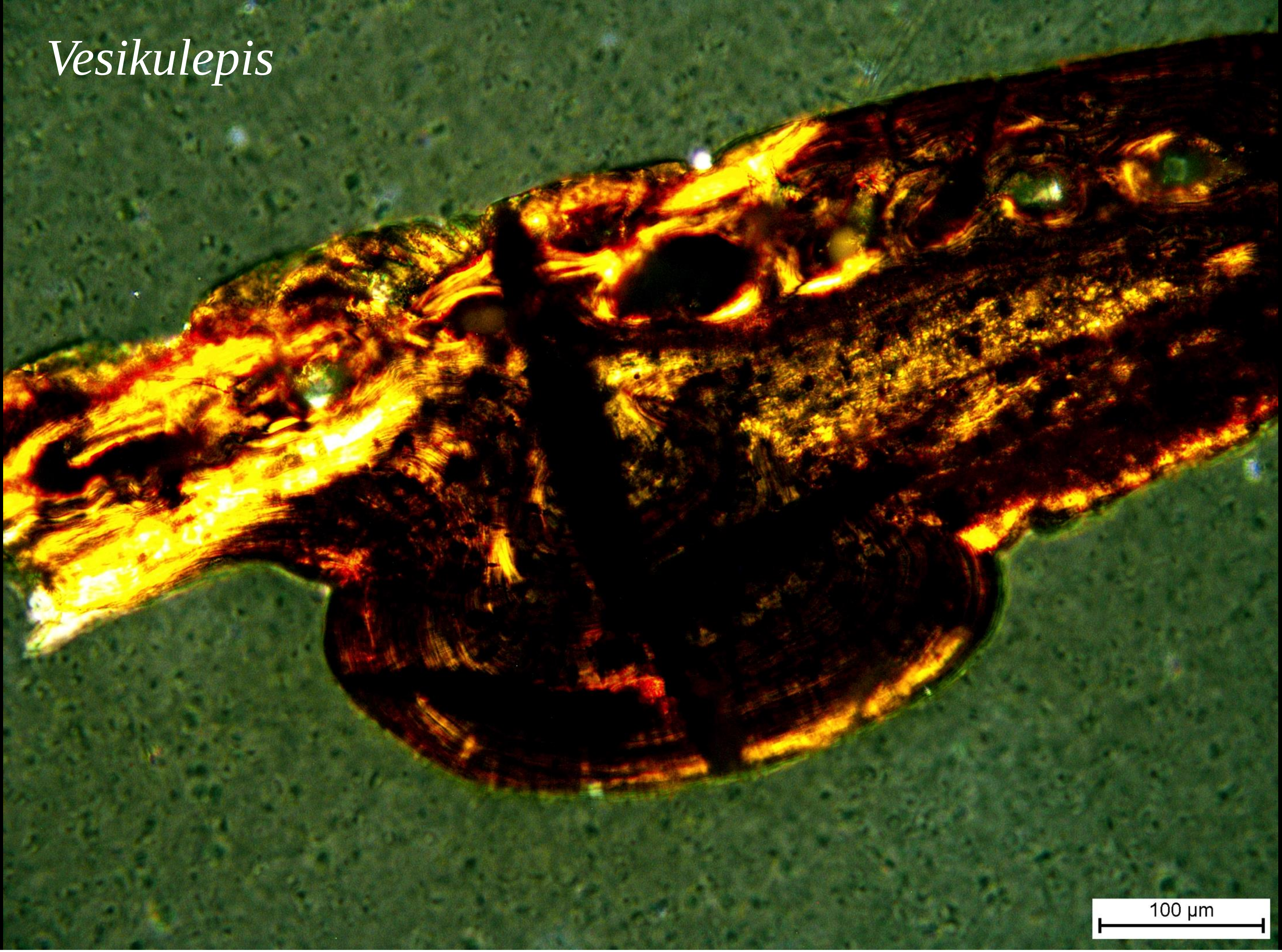

Fig. 50. Specimen NHMUK PV P73710. LM thin section of a *Vesikulepis* body scale under cross polarized light.
