# Supplementary material for: Histology and affinity of anaspids, and the early evolution of the vertebrate dermal skeleton
Source: Proc Biol Sci. 2016 Mar 16;283(1826):20152917. doi: 10.1098/rspb.2015.2917 (PMC4810860; doi:10.1098/rspb.2015.2917)
Supplement: Electronic Supplementary Information 2 - Phylogenetics [file rspb20152917supp2.pdf]

Supplementary information
2 – Phylogenetic analysis

Table of Contents

Character revisions 1
Character list 1
Character matrix 6
Phylogenetic topology tests 7
Implied weighting 8
Results 9
References 19

Character revisions

The character list is based principally on that of Donoghue et al. (2000) as well as the recent revisions of this matrix (Blom, 2012; Conway Morris and Caron, 2014; Sansom et al., 2010). The coding for all taxa has been revised (see below for a list of changes).

Characters relating to the dermal skeleton have been heavily modified in order to alleviate problems resulting from compound characters - characters that require two or more conditional qualifiers in order to specify the state. The problem with compound characters is that the qualifiers are independent variables in there own right, and as such may supply independent phylogenetic information (Brazeau, 2011). Thus coding only specific instances of relationships between variables could potentially bias any independent signal present inherently within the variables themselves (Brazeau, 2011). For example, the character: “three-layered exoskeleton consisting of a basal lamella, middle spongy (or cancellar) layer and a superficial (often ornamented) layer: absent = 0, present = 1” is a compound character that contains three qualifiers: the presence/absence of a superficial layer, the presence/absence of a spongy or cancellous basal layer the presence/absence of a basal lamella. These variables are known to occur independently of each other, yet this phylogenetic variation is not accounted for in the original coding scheme. To alleviate this problem, the character in question has been split into three independent characters.

New contingent characters have been added, to reflect the differences in dermal skeletal histology between anaspids and other skeletonising vertebrates. For example, we have added the character “tubular dentine present in dermal skeleton”, which is contingent on the character “dentine present in dermal skeleton”. This character coding strategy discriminates between the spheritic dentine present in anaspids and the ortho/meso/semidentine (i.e. dentine containing canuliculae) present in other skeletonising vertebrates whilst alleviating errors implicit in multistate treatment of the same variables (Brazeau, 2011).

Character list

(a) Brain, sensory and nervous system

1. **Neural crest absent = 0, present = 1.** Unchanged

2. **Olfactory peduncles absent = 0, present = 1**. Unchanged.
3. **Pineal organ (extra-ocular photoreceptor region expressing pineal opsins) absent = 0, present = 1.** Unchanged.
4. **Adenohypophysis absent = 0, present = 1.** Coding for galeaspids changed from ? to 1.
5. **Adenohypophysis simple = 0, compartmentalized = 1.** Unchanged.
6. **Optic tectum absent = 0, present = 1.** Unchanged.
7. **Cerebellar primordia absent = 0, present = 1.** Unchanged.
8. **Pretrematic branches in branchial nerves absent = 0, present = 1.** Unchanged.
9. **Flattened spinal chord absent = 0, present = 1.** Unchanged.
10. **Ventral and dorsal spinal nerve roots united, absent = 0, present = 1.** Unchanged.
11. **Mauthner fibres in central nervous system absent = 0, present = 1.** Unchanged.
12. **Retina absent = 0, present = 1.** Unchanged.
13. **Olfactory organ with external opening absent = 0, present = 1.** Unchanged.
14. **Nasohypophyseal opening serving respiration (nasohypophyseal duct) absent = 0, present = 1.** Unchanged
15. **Single nasohypophyseal opening, absent = 0, present = 1.** Unchanged.
16. **Position of nasohypophyseal opening: terminal = 0, dorsal = 1.** Coding for *Jamoytius* changed from 1 to ?
17. **Olfactory organ unpaired = 0, paired = 1.** Coding for Osteostraci changed from ? to 1.
18. **Extrinsic eye musculature absent = 0, present = 1.** Coding for galeaspids changed from ? to 1, for conodonts from 1 to ?
19. **Otic capsule anterior to branchial series, absent = 0, present = 1.** Unchanged.
20. **Semicircular canals in labyrinth absent = 0, present = 1.** Unchanged.
21. **Vertical semicircular canals forming loops that are separate from the roof of the utriculus, absent = 0, present = 1.** Character description qualified further by adding that the loops of the SCC are separate from the roof of the utriculus.
22. **Externally open endolymphatic ducts absent = 0, present = 1.** Coding for anaspids changed from ? to 0.
23. **Electroreceptive cells absent = 0, present = 1.** Unchanged.
24. **Sensory lines absent = 0, present = 1.** Unchanged.
25. **Sensory-lines on head only = 0, on head plus body = 1.** Coding for *Astraspis* changed from 0 to ?
26. **Sensory-line enclosed in grooves = 0, enclosed in canals = 1.** Unchanged.

(b) Mouth and branchial system

27. **Pouch-shaped gills absent = 0, present = 1.** Coding for *Astrapis,* Arandaspids, Anaspids changed from 1 to ?, for *Euphanerops* changed from 0 to ?
28. **Single confluent branchial opening, absent = 0, present = 1.** Coding for *Euphanerops* changed from 0 to ?
29. **Elongate branchial series: more than 10 gill pouches/slits = 0, fewer than 10 = 1.** Unchanged.
30. **Gill openings lateral and arranged in slanting row, absent = 0, present = 1.** Coding for *Euphanerops* changed from 1 to ?, Heterostraci changed from 0 to inapplicable.
31. **Position of gill openings: laterally = 0, ventrally = 1.** Coding for *Euphanerops* changed from 0 to ?, *Turinia* changed from ? to 0.
32. **Opercular flaps associated with gill openings, absent = 0, present = 1.** Coding for Galeaspida changed from 0 to ?
33. **Endodermal gill lamellae, absent = 0, present** = 1. Unchanged.
34. **Gill lamellae with filaments, absent = 0, present = 1. Unchanged**

35. **Mouth terminal = 0, ventral = 1.** Coding for *Loganellia* and *Turinia* changed from 0 to ?, for *Jamoytius, Euphanerops* from 1 to 0, for jawed vertebrates from 0 to 1.
36. **Oral hood absent = 0, present = 1.** Coding for Cephalochordata changed from 1 to 0, for *Astraspis* from ? to 0, for Euconodonta from 0 to ?
37. **Velum absent = 0, present = 1.** Coding for Osteostraci and Galeaspida from ? to 1.

(c) Circulatory system

38. **Multi-chamber heart absent = 0, present = 1.** Unchanged.
39. **Closed pericardium absent = 0, present = 1.** Coding for Tunicata changed from 1 to 0, for Cephalochordata from inapplicable to 0.
40. **Open blood system absent = 0, present = 1.** Unchanged.
41. **Paired dorsal aortae absent = 0, present = 1.** Unchanged.
42. **Large lateral head vein absent = 0, present = 1.** Unchanged.
43. **Lymphocytes absent = 0, present = 1.** Unchanged.
44. **Subaponeurotic vascular plexus absent = 0, present = 1.** Coding for Arandaspida changed from ? to 1.

(d) Fins and fin-folds

45. **Dorsal fin: separate dorsal fin absent = 0, present = 1.** Coding for Arandaspida changed from ? to 1 and for *Euphanerops* and *Jamoytius* from ? to 0.
46. **Dorsal fin originates at posterior of branchial series = 0, restricted to posterior of trunk and/or caudal region = 1.** Coding changed such that this character is contingent on character 45: Dorsal fin.
47. **Anal fin separate, absent = 0, present = 1.** Unchanged.
48. **Fin ray supports, absent = 0, present = 1.** Coding for *Turinia, Loganellia,* Heterostraci changed from 0 to 1, for Arandaspida from ? to 1.
49. **Paired antero-posterior skin folds absent = 0, present = 1.** Coding for Euconodonta changed from 0 to ?, for *Turinia, Loganellia* from 1 to 0.
50. **Constricted pectoral fins with endoskeletal elements absent = 0, present = 1.** Unchanged.
51. **Pelvic fins/flap, absent = 0, present = 1.** Coding for *Turinia* changed from ? to 0.
52. **Tail shape: no distinct lobes developed = 0, ventral lobe much larger than dorsal = 1, dorsal lobe much larger than ventral = 2, dorsal and ventral lobes almost equally developed = 3.** Coding for *Loganellia* changed from 3 to 1.
53. **Chordal disposition relative to tail development, isochordal = 0, hypochordal = 1, hyperchordal = 2.** Unchanged.
54. **Preanal median fold absent = 0, present = 1.** Coding for Tunicata changed from ? to inapplicable.

(e) Skeletal

55. **Ability to synthesise creatine phosphatase absent = 0, present = 1.** Unchanged.
56. **Visceral arches fused to the neurocranium absent = 0, present =1.** Unchanged.
57. **Keratinous teeth absent = 0, present = 1.** Coding for Tunicata changed from ? to inapplicable, for anaspids, Arandaspida, *Astraspis,* Galeaspida, Heterostraci, *Loganellia*, Osteostraci, *Turinia* from ? to 0.
58. **Circumoral teeth absent = 0, present = 1.** Coding for *Turinia* changed from ? to 0.
59. **Circumoral teeth arranged in radiating series, absent = 0, present = 1.** Coding for *Turinia* changed from ? to inapplicable.
60. **Trematic rings absent = 0, present = 1.** Unchanged.
61. **Arcualia absent = 0, present = 1.** Coding for Myxinoidea from ? to 1.

62. **Piston cartilage and apical plate, absent = 0, present = 1.** Unchanged.
63. **Midline retractor muscle and paired protractor muscles, absent = 0, present = 1.** Coding changed for jawed vertebrates changed from inapplicable to 0.
64. **Transversely biting teeth absent = 0, present 1.** The wording of this character description has been modified in order reduce ambiguity; coding reflects Gess et al. (2006), Coding for all ostracoderms changed from ? to 0.
65. **Jaws (dorsoventral bite), absent 0, present = 1.** Unchanged.
66. **Chondroitin 6-sulphate in cartilage, absent = 0, present = 1.** Unchanged.
67. **Braincase with lateral walls, absent = 0, present = 1.** Unchanged.
68. **Neurocranium entirely closed dorsally and covering the brain, absent = 0, present = 1.** Unchanged.
69. **Occiput enclosing vagus and glossopharyngeal nerves, absent = 0, present = 1.** Unchanged.
70. **Annular cartilage absent = 0, present = 1.** Coding for Heterostraci, Arandaspida, *Loganellia, Turinia* changed from ? to 0, for Euconodonta changed from 0 to ?
71. **Large oral disc absent = 0, present = 1.** Coding for Euconodonta changed from 0 to ?
72. **Tentacle cartilages; absent = 0, present = 1.** Coding for *Astraspis* changed from ? to 0.
73. **Trunk dermal skeleton absent = 0, present = 1.** Coding for *Euphanerops* changed from ? to 1.
74. **Perichondral bone absent = 0, present = 1.** Coding for *Euphanerops* changed from 0 to ?, for *Jamoytius* from ? to 0.
75. **Calcified cartilage absent = 0, present = 1.** Coding for *Turinia* changed from ? to 0.
76. **Cartilage composed of huge clumped chondrocytes, absent = 0, present = 1.** Unchanged.
77. **Calcified dermal skeleton absent = 0, present = 1.** Coding changed for Euconodonta from 1 to 0, for *Euphanerops* from 0 to 1.
78. **Superficial (often ornamented) layer of the dermal skeleton, absent = 0, present = 1.** Character modified from Donoghue et al. (2000) "Three-layered exoskeleton consisting of a basal lamella, middle spongy (or cancellar) layer and a superficial (often ornamented) layer." This is considered a compound character and has been atomized into three independently variable characters. Contingent on character 77 (state 1).
79. **Osteonal vascular middle layer of the dermal skeleton, absent = 0, present = 1.** Character modified from Donoghue et al. (2000), see above. The wording of this character description has been modified in order reduce ambiguity. Contingent on character 77 (state 1).
80. **Basal laminated layer of the dermal skeleton, absent = 0, present = 1.** Character modified from Donoghue et al. (2000), see above. The wording of this character description has been modified in order reduce ambiguity. Contingent on character 77 (state 1).
81. **Isopedine in dermal skeleton, absent = 0, present = 1.** New Character. Contingent on character 80 (state 1).
82. **Cellular bone, absent = 0, present = 1.** Coding changed for Euconodonta from 1 to 0, for *Euphanerops* from 0 to ? Changed Arandaspida from 0 to 1.
83. **Dentine in dermal skeleton absent = 0, present = 1.** The wording of this character description has been modified in order reduce ambiguity. Contingent on character 77 (state 1). Coding for *Jamoytius, Euphanerops*, Galeaspida changed from 0 to ?, for Euconodonta from 1 to 0.
84. **Tubular dentine in dermal skeleton, absent = 0, present = 1.** New character. contingent on character 83 (state 1).
85. **Tubular dentine present as mesodentine = 0, orthodentine = 1.** Wording changed such that this character is contingent on character 84 (state 1). Coding for Arandaspida changed

from ? to 0, for Euconodonta from 1 to inapplicable.

86. **Enameloid in dermal skeleton absent = 0, present = 1.** Character changed to reflect that no early vertebrates possess enamel, just enameloid since the former is an apomorphy of Osteichthyes. Coding changed for *Birkenia* and *Rhyncholepis* from inapplicable to 1*, Jamoytius, Euphanerops* from inapplicable to ?, for Osteostraci, Heterostraci, *Loganellia, Turinia* from 0 to 1, for Euconodonta from 1 to 0. Contingent on character 77 (state 1).
87. **Cancellar layer in exoskeleton, with honeycomb-shaped cavities, absent = 0, present = 1.** Contingent on character 79 (state 1). Changed Astraspis, Jawed vertebrates and osteostracans to 0.
88. **Scale shape: diamond-shaped = 0, rod-shaped = 1.** Coding changed for *Euphanerops, Jamoytius* from 0 to ?, for Osteostraci from 0 to 1, for Galeaspida from 0 to ?, *Euconodonta, Birkenia, Rhyncholepis, Pterygolepis* from ? to inapplicable.
89. **Costulate tubercles, absent = 0, present = 1.** New character. Defines tubercles with ridges/ribs/buttresses. Contingent on character 78 (state 1)
90. **Oak-leaf-shaped tubercles, absent = 0, present = 1.** Wording changed such that this character is contingent on character 89 (state 1).
91. **Oral plates absent = 0, present = 1.** Contingent on character 77 (state 1). Coding changed for Galeaspida from 1 to ?
92. **Denticles in pharynx absent = 0, present = 1.** Coding changed for *Astraspis*, *Jamoytius*, anaspids, *Euphanerops*, from ? to 0.
93. **Dermal head covering in adult state absent = 0, present = 1.** Contingent on character 77 (state 1). Coding for *Astraspis* changed from 0 to 1.
94. **Large unpaired ventral and dorsal dermal plates on head, absent = 0, present = 1.** Contingent on character 93 (state 1). Coding for *Astraspis* changed from 1 to 0.
95. **Pineal plate, absent = 0, present = 1.** New character. Contingent on character 77 (state 1).
96. **Massive endoskeletal head shield covering the gills dorsally, absent = 0, present = 1.** Coding changed for jawed vertebrates from 0 to 1, for Arandaspida, *Astraspis* from ? to 0.
97. **Sclerotic ossicles absent = 0, present = 1.** Coding changed for *Astraspis, Turinia* from ? to 0, for Galeaspida from 0 to ?
98. **Ossified endoskeletal sclera encapsulating the eye, absent = 0, present = 1.** Coding changed for Galeaspida from 0 to ?

(g) Miscellaneous

99. **High blood pressure, absent = 0, present = 1.** Unchanged.
100. **Hyperosmoregulation, absent = 0, present = 1.** Unchanged.
101. **Male gametes shed directly through the coelom, absent = 0, present = 1.** Unchanged.
102. **Forward migration of postotic myomeres, absent = 0, present = 1.** Unchanged.
103. **Larval phase, absent = 0, present = 1.** Unchanged

(h) Additional characters due to change in coding strategy and the previous characters upon which they are contingent

104. **Pineal opening covered = 0, uncovered = 1.**
105. **External nasal opening single = 0, paired = 1.** Character coding changed to inapplicable for Arandaspida, *Astraspis*, Heterostraci, *Loganellia*, *Turinia*.
106. **Number of semicircular canals one = 0, two = 1, three = 2.**
107. **Neuromasts in sensory lines absent = 0, present = 1.** Coding changed for Arandaspida, *Astraspis*, Heterostraci, *Loganellia* from ? to 1.
108. **Relative position of atrium and ventricle of heart: well separated = 0, close to**

each other = 1.

109. Lymphocytes antigen receptors VLR = 0, T and B = 1.

110. Paired antero-posterior skin folds extend along the trunk = 0, anterior only = 1.

111. Ventral arcualia: absent = 0, present = 1. New character.

112. Odontodes: Polyodontode = 0, monodontodes = 1. New character. Coding for Euconodonta changed from 1 to inapplicable.

113. Head skeleton macroplated = 0, microplated = 1. Contingenet on character 93 (state 1).

114. Inflected myomeres present in post-larval stage (chevron, Z or W shaped) absent = 0, present = 1.

115. Complex myomere shape: z-shaped = 0, w-shaped = 1. Simple myomeres inapplicable.

(i) Characters for anaspid interrelations.

116. Triradiate postbranchial spine absent = 0, present = 1. From Blom (2012). Contingent on character 77 (state 1).

117. Median dorsal ridge scales absent = 0, present = 1. From Blom (2012). Contingent on character 77 (state 1).

118. Hook-shaped median dorsal ridge scales absent = 0, present = 1. From Blom (2012). Contingent on character 77 (state 1).

119. Canal system in body scales absent = 0, present = 1. From Blom (2012). The wording of this character description has been modified in order reduce ambiguity. Contingent on character 73 (state 1).

120. Body scales with visceral ribs absent = 0, present = 1. New character. Contingent on character 73 (state 1).

Character matrix

The character matrix is available to download in nexus format at datadryad.org: doi:10.5061/dryad.k2qc4

Cephalochordata 0?10?00000000----??0-?00--
0000001000100010000?000000010000?00??00?00000000000----0------------000000011??????--
1?-----

Tunicata 1?10?0000?000----??0-?00--
010??000000000000000?0000000?0000?00??00?00000000000----0------------000000011??????--
0?-----

Myxinoidea
10010100110111101011000100100100100011001011 0?010000111110?111110000000100010---
-0------------00000010?00000?1--11-----

Petromyzontida
1011111010111011111100111010110011011111001011010001101111111111011001100 0010---
-0------------00011111101110?0--11-----

Heterostraci ?11???1?????0???1?0110?111111?00?100???????10?010003101?00-
?1??00????000100?1111101111111110111000?????0?11????11??01010

Astraspis
??1???????????????????0?1?0?01100???0???????1????000???1?0??????00??????0100?1111101
1110011?010?000?????0??1????11??0101?

Arandaspida ??1?????????1??????????110?00100??00???????10?01000??01?00-
????00????0001???1111111011111101100 10?????0??1????111?0??10

Jamoytius
??????????????1????????????0010???00????????0???100??01?????????0????10010??1???????
???1?0000-?000??????0????0??-110--??
Euphanerops
??????????????????????????1?0????100????????0?1110011?1?????1???0????1001??11???????
???1?0000-?000???????????01?-1?0--??
Osteostraci ?011?11?????1011110111?111101?11?110111?01?111010102201100-
?1??00?1110001110111111101011010101111????10111???111101010
Galeaspida ?111?11?????1111110111?111101?1??1101????1?1????000??01100-
????00?1110001010110110???10010?01001???????1011?????1??0??1?
Loganellia ??????????????????????111?01101???0????????11110001101?00-
????00????000100?1100-01101?01001100000????????1????00??00-00
Turinia ?????????????????????????1011?1???0???????????1000?1?1?00-
????00????000100?1100-01111?01001100000?????????????00??00-00
Jawed_vertebrates
1111111101111?0?111111111100100101100111111011110112201000-
01??0111110001110111111111100100110111111000012111?1111101010
Euconodonta ??????????????????????????1?1???????????????0?01?001101?00-
????10???????000?0----0------------000?????????????-?1?0----
Birkenia ??1?????????1?11?????0?????01100??00????????0?110001111?00-
????00?????00100?11010010-1-10010101000?????10????-?101?11101
Pharyngolepis ??1?????????1?11?????0?100?00100??00????????0?111001101?00-
????00?????00100?1??????????10010100000?????10?1??0??01?110?1
Rhyncholepis ??1?????????1?11?????0?100?01100??00????????0?111001111?00-
????00?????00100?11010010-1-10010101000?????10?1??0?101?11111
Pterygolepis ??1?????????1?11?????0?????01100??00????????0?110001111?00-
????00?????00100?1?01?0????-10010101000?????10????-?101?11101
Cowielepis ??1?????????1?11?????0?????01100??0?????????0?111001?01?00-
????00?????00100?1???????????1?0?0101000?????10????0??01?111??
Lasanius ???????????????????????????01100??00????????0???0001101?00-
????00?????00100?1????????????00000-000???????????-??-??111??

Phylogenetic topology tests

We conducted Templeton tests and one-tailed Kishino-Hasegawa tests, using Paup 4.0b 10, in order to access whether the data is decisive between the conflicting hypotheses of anaspid affinity. The results are displayed in Table 1, below. Phylogenetic topology tests reject the hypothesis that anaspids are the sister-group to jawed vertebrates, but fail to the reject alternate hypotheses of anaspid affinity.

Hypothesis	Backbone Constraint(s)	Templeton	KH	1-tailed KH	Reject
A	(Cephalochordata,((Cyclostomi, Anaspida)(Pteraspidomorphi, Thelodonti, Galeaspida, Osteostraci, jawed vertebrates)))	0.3173	0.3193	0.6383	×
B	(Cephalochordata,((Cyclostomi)((Anaspida), (Pteraspidomorphi, Thelodonti, Galeaspida, Osteostraci, jawed vertebrates))))	0.5637	0.3193	0.6383	×
C	(Cephalochordata,((Cyclostomi)((Pteraspido morphi),(Anaspida, Thelodonti, Galeaspida, Osteostraci, jawed vertebrates))))	1	1	1	×
D	(Cephalochordata,((Cyclostomi)(Pteraspido morphi, Thelodonti, Galeaspida, Osteostraci(Anaspida, jawed vertebrates))))	0.0044	0.0025	0.05	✓

Table 1. Results of phylogenetic topology tests conducted using Paup 4.0b 10. Hypothesis *D* is rejected.

Implied weighting

In order to access the impact of homoplastic characters on tree topology, we implemented implied weighting following Goloboff (1993). Under Goloboff's method, each additional step in a transformation series is penalized less strongly than the previous step. The penalty for additional steps is determined by a concavity constant (k). The lower the value of k, the less weight is attached to subsequent steps. Thus, at very low k values (k = <1.5), transformation series with more than 1 additional step are down-weighted such that they provide almost no phylogenetic information. On the other hand, at high k values (k = >30), down-weighting of transformation series with more than 1 additional step is negligible. Thus implied weighting at high values of k approximates equal weighting. There is no objective means to select the k value, thus we have used the TNT script from Smith and Ortega-Hernández (2014), which repeats an analysis over 100 different k values including 99 derived from a log normal distribution as well as equal weighting (k = ∞). The results of these analyses are listed below in TNT format (note that 0 length branches are collapsed in the strict consensus trees).

Presenting the results of the implied weighting analysis diagrammatically has proved challenging. In order to present all relationships supported by all values of k, we used consensus networks at threshold 0 using the program SplitsTree (Huson and Bryant, 2006). We produced three different consensus networks using three different sets of input trees. Firstly we input 100 SCC trees from 100 different k values (see Fig. 4C, main text). The advantage of this network is that edge lengths are proportional to the number of weighting parameters that support the branch. The disadvantage is that conflicting relationships in any given weighting parameter are input as polytomies and are thus not presented in the diagram. We produced a second network by inputting all MPT's derived from all values of k (Fig. 1). The advantage of this network is that it shows all conflicting topologies, including conflicting topologies at a single k value. The disadvantage is that edge lengths of the network are proportional to the number of trees which support the branch but not necessarily proportional to the number of k values that support the branch because different k values can produce different numbers of MPT's. The problems inherent in both of these networks can be avoided if the number of MPT's at each concavity constant is normalized (Fig. 2). This can be achieved using the following:

k_i , $i = 1, 2, \ldots N$

Where k is the concavity constant

$A_i =$ Number of MPT's at concavity constant = k_i

$$X = \prod_{i=1}^{N} A_i = A_1, A_2, \dots A_N$$

$$B_i = \frac{X}{A_i}$$

Finally, factorize B_i

$$b_i = \frac{B_i}{GCD(\underline{B})}$$

Where $\underline{B} = (B_1, B_2, \dots B_N)$ and GCD = greatest common divisor

A consensus network produced by inputting normalized MPT's will display all conflicting topologies from all values of k. Edge length (V_e) equals the sum of the proportion of MPT's with edge (*e*) at each value of k.

$C_i =$ A_i with edge = *e*

$$V_e = \sum_{1}^{100} \frac{C_i}{A_i}$$

In simplistic terms, the longer an edge, the more frequently it is recovered under different concavity constants. An edge recovered in each MPT at each concavity constant will have a length of 100. On the other hand, an edge common to only a subset of the MPT's at a single concavity constant will have a length equal to <1. Thus extremely uncommon edges are included in the splits consensus. A normalized splits consensus network can therefore be used to visualize the frequency of all edges recovered under all weighting parameters.

Results

Concavity constant = 0.118

MPT's: (0 (1 ((14 (2 3))((5 (4 6))(((7 8)(16 (19 (17 (20 (15 18))))))((10 (9 13))(11 12)))))))*
(0 (1 ((14 (2 3))((5 (4 6))(((7 8)(16 ((20 (15 18))(17 19))))((10 (9 13))(11 12)))))))*
(0 (1 ((14 (2 3))((5 (4 6))(((7 8)(16 (19 (17 (18 (15 20))))))((10 (9 13))(11 12)))))));
Strict: (0 (1 ((14 (2 3))((5 (4 6))(((7 8)(16 (17 19 (15 18 20))))((10 (9 13))(11 12)))))));

Concavity constant = 0.183

MPT's: (0 (1 ((14 (2 3))((5 (4 6))(((7 8)(16 (19 (17 (20 (15 18))))))((10 (9 13))(11 12)))))))*
(0 (1 ((14 (2 3))((5 (4 6))(((7 8)(16 ((20 (15 18))(17 19))))((10 (9 13))(11 12)))))))*
(0 (1 ((14 (2 3))((5 (4 6))(((7 8)(16 (19 (17 (18 (15 20))))))((10 (9 13))(11 12)))))));
Strict: (0 (1 ((14 (2 3))((5 (4 6))(((7 8)(16 (17 19 (15 18 20))))((10 (9 13))(11 12)))))));

Concavity constant = 0.242

MPT's: (0 (1 ((14 (2 3))((5 (4 6))(((7 8)(16 (19 (17 (20 (15 18))))))((10 (9 13))(11 12)))))))*
(0 (1 ((14 (2 3))((5 (4 6))(((7 8)(16 (17 (19 (20 (15 18))))))((10 (9 13))(11 12)))))))*

(0 (1 ((14 (2 3))((5 (4 6))(((7 8)(16 (19 (17 (18 (15 20))))))((10 (9 13))(11 12)))))));
Strict: (0 (1 ((14 (2 3))((5 (4 6))(((7 8)(16 (17 19 (15 18 20))))((10 (9 13))(11 12)))))));

Concavity constant = 0.298
MPT's: (0 (1 ((14 (2 3))((5 (4 6))(((7 8)(16 (19 (17 (20 (15 18))))))((10 (9 13))(11 12)))))))*
(0 (1 ((14 (2 3))((5 (4 6))(((7 8)(16 (17 (19 (20 (15 18))))))((10 (9 13))(11 12)))))))*
(0 (1 ((14 (2 3))((5 (4 6))(((7 8)(16 (19 (17 (18 (15 20))))))((10 (9 13))(11 12)))))));
Strict: (0 (1 ((14 (2 3))((5 (4 6))(((7 8)(16 (17 19 (15 18 20))))((10 (9 13))(11 12)))))));

Concavity constant = 0.354
MPT's: (0 (1 ((14 (2 3))((5 (4 6))(((7 8)(16 (19 (17 (20 (15 18))))))((10 (9 13))(11 12)))))))*
(0 (1 ((14 (2 3))((5 (4 6))(((7 8)(16 (17 (19 (20 (15 18))))))((10 (9 13))(11 12)))))))*
(0 (1 ((14 (2 3))((5 (4 6))(((7 8)(16 (19 (17 (18 (15 20))))))((10 (9 13))(11 12)))))));
Strict: (0 (1 ((14 (2 3))((5 (4 6))(((7 8)(16 (17 19 (15 18 20))))((10 (9 13))(11 12)))))));

Concavity constant = 0.409
MPT's: (0 (1 ((14 (2 3))((5 (4 6))(((7 8)(16 (19 (17 (20 (15 18))))))((10 (9 13))(11 12)))))))*
(0 (1 ((14 (2 3))((5 (4 6))(((7 8)(16 (17 (19 (20 (15 18))))))((10 (9 13))(11 12)))))))*
(0 (1 ((14 (2 3))((5 (4 6))(((7 8)(16 (19 (17 (18 (15 20))))))((10 (9 13))(11 12)))))));
Strict: (0 (1 ((14 (2 3))((5 (4 6))(((7 8)(16 (17 19 (15 18 20))))((10 (9 13))(11 12)))))));

Concavity constant = 0.464
MPT's: (0 (1 ((14 (2 3))((5 (4 6))(((7 8)(16 (19 (17 (20 (15 18))))))((10 (9 13))(11 12)))))))*
(0 (1 ((14 (2 3))((5 (4 6))(((7 8)(16 (17 (19 (20 (15 18))))))((10 (9 13))(11 12)))))))*
(0 (1 ((14 (2 3))((5 (4 6))(((7 8)(16 (19 (17 (18 (15 20))))))((10 (9 13))(11 12)))))));
Strict: (0 (1 ((14 (2 3))((5 (4 6))(((7 8)(16 (17 19 (15 18 20))))((10 (9 13))(11 12)))))));

Concavity constant = 0.521
MPT's: (0 (1 ((14 (2 3))((5 (4 6))(((7 8)(16 (17 (19 (20 (15 18))))))((10 (9 13))(11 12)))))))*
(0 (1 ((14 (2 3))((5 (4 6))(((7 8)(16 (19 (17 (20 (15 18))))))((10 (9 13))(11 12)))))))*
(0 (1 ((14 (2 3))((5 (4 6))(((7 8)(16 (19 (17 (18 (15 20))))))((10 (9 13))(11 12)))))));
Strict: (0 (1 ((14 (2 3))((5 (4 6))(((7 8)(16 (17 19 (15 18 20))))((10 (9 13))(11 12)))))));

Concavity constant = 0.577
MPT's: (0 (1 ((14 (2 3))((5 (4 6))(((7 8)(16 (19 (17 (20 (15 18))))))((10 (9 13))(11 12)))))))*
(0 (1 ((14 (2 3))((5 (4 6))(((7 8)(16 (17 (19 (20 (15 18))))))((10 (9 13))(11 12)))))))*
(0 (1 ((14 (2 3))((5 (4 6))(((7 8)(16 (19 (17 (18 (15 20))))))((10 (9 13))(11 12)))))));
Strict: (0 (1 ((14 (2 3))((5 (4 6))(((7 8)(16 (17 19 (15 18 20))))((10 (9 13))(11 12)))))));

Concavity constant = 0.635
MPT's: (0 (1 ((14 (2 3))((5 (4 6))(((7 8)(16 (19 (17 (20 (15 18))))))((10 (9 13))(11 12)))))))*
(0 (1 ((14 (2 3))((5 (4 6))(((7 8)(16 (17 (19 (20 (15 18))))))((10 (9 13))(11 12)))))))*
(0 (1 ((14 (2 3))((5 (4 6))(((7 8)(16 (19 (17 (18 (15 20))))))((10 (9 13))(11 12)))))));
Strict: (0 (1 ((14 (2 3))((5 (4 6))(((7 8)(16 (17 19 (15 18 20))))((10 (9 13))(11 12)))))));

Concavity constant = 0.694
MPT's: (0 (1 ((14 (2 3))((5 (4 6))(((7 8)(16 (17 (19 (20 (15 18))))))((10 (9 13))(11 12)))))))*
(0 (1 ((14 (2 3))((5 (4 6))(((7 8)(16 (19 (17 (20 (15 18))))))((10 (9 13))(11 12)))))))*
(0 (1 ((14 (2 3))((5 (4 6))(((7 8)(16 (19 (17 (18 (15 20))))))((10 (9 13))(11 12)))))));
Strict: (0 (1 ((14 (2 3))((5 (4 6))(((7 8)(16 (17 19 (15 18 20))))((10 (9 13))(11 12)))))));

Concavity constant = 0.754
MPT's: (0 (1 ((14 (2 3))((5 (4 6))(((7 8)(16 (19 (17 (20 (15 18))))))((10 (9 13))(11 12)))))))*
(0 (1 ((14 (2 3))((5 (4 6))(((7 8)(16 (17 (19 (20 (15 18))))))((10 (9 13))(11 12)))))))*
(0 (1 ((14 (2 3))((5 (4 6))(((7 8)(16 (19 (17 (18 (15 20))))))((10 (9 13))(11 12)))))));
Strict: (0 (1 ((14 (2 3))((5 (4 6))(((7 8)(16 (17 19 (15 18 20))))((10 (9 13))(11 12)))))));

Concavity constant = 0.815
MPT's: (0 (1 ((14 (2 3))((5 (4 6))(((7 8)(16 (19 (17 (20 (15 18))))))((10 (9 13))(11 12)))))))*

(0 (1 ((14 (2 3))((5 (4 6))(((7 8)(16 (17 (19 (20 (15 18))))))((10 (9 13))(11 12)))))))*
(0 (1 ((14 (2 3))((5 (4 6))(((7 8)(16 (19 (17 (18 (15 20))))))((10 (9 13))(11 12)))))));
Strict: (0 (1 ((14 (2 3))((5 (4 6))(((7 8)(16 (17 19 (15 18 20))))((10 (9 13))(11 12)))))));

Concavity constant = 0.878
MPT's: (0 (1 ((14 (2 3))((5 (4 6))(((7 8)(16 (19 (17 (20 (15 18))))))((10 (9 13))(11 12)))))))*
(0 (1 ((14 (2 3))((5 (4 6))(((7 8)(16 (17 (19 (20 (15 18))))))((10 (9 13))(11 12)))))))*
(0 (1 ((14 (2 3))((5 (4 6))(((7 8)(16 (19 (17 (18 (15 20))))))((10 (9 13))(11 12)))))));
Strict: (0 (1 ((14 (2 3))((5 (4 6))(((7 8)(16 (17 19 (15 18 20))))((10 (9 13))(11 12)))))));

Concavity constant = 0.943
MPT's: (0 (1 ((14 (2 3))((5 (4 6))(((7 8)(16 (19 (17 (20 (15 18))))))((10 (9 13))(11 12)))))))*
(0 (1 ((14 (2 3))((5 (4 6))(((7 8)(16 (17 (19 (20 (15 18))))))((10 (9 13))(11 12)))))))*
(0 (1 ((14 (2 3))((5 (4 6))(((7 8)(16 (19 (17 (18 (15 20))))))((10 (9 13))(11 12)))))));
Strict: (0 (1 ((14 (2 3))((5 (4 6))(((7 8)(16 (17 19 (15 18 20))))((10 (9 13))(11 12)))))));

Concavity constant = 1.008
MPT's: (0 (1 ((14 (2 3))((5 (4 6))((16 ((19 (20 (7 8)))(17 (15 18))))((10 (9 13))(11 12)))))));
Strict: (0 (1 ((14 (2 3))((5 (4 6))((16 (19 (20 (7 8))(17 (15 18))))((10 (9 13))(11 12)))))));

Concavity constant = 1.076
MPT's: (0 (1 ((14 (2 3))((5 (4 6))((16 ((19 (20 (7 8)))(17 (15 18))))((10 (9 13))(11 12)))))));
Strict: (0 (1 ((14 (2 3))((5 (4 6))((16 (19 (20 (7 8))(17 (15 18))))((10 (9 13))(11 12)))))));

Concavity constant = 1.145
MPT's: (0 (1 ((14 (2 3))((5 (4 6))((16 ((19 (20 (7 8)))(17 (15 18))))((10 (9 13))(11 12)))))));
Strict: (0 (1 ((14 (2 3))((5 (4 6))((16 (19 (20 (7 8))(17 (15 18))))((10 (9 13))(11 12)))))));

Concavity constant = 1.217
MPT's: (0 (1 ((14 (2 3))((5 (4 6))((16 ((19 (20 (7 8)))(17 (15 18))))((10 (9 13))(11 12)))))));
Strict: (0 (1 ((14 (2 3))((5 (4 6))((16 (19 (20 (7 8))(17 (15 18))))((10 (9 13))(11 12)))))));

Concavity constant = 1.290
MPT's: (0 (1 ((14 (2 3))((5 (4 6))((16 ((19 (20 (7 8)))(17 (15 18))))((10 (9 13))(11 12)))))));
Strict: (0 (1 ((14 (2 3))((5 (4 6))((16 (19 (20 (7 8))(17 (15 18))))((10 (9 13))(11 12)))))));

Concavity constant = 1.365
MPT's: (0 (1 ((14 (2 3))((5 (4 6))((16 ((19 (20 (7 8)))(17 (15 18))))((10 (9 13))(11 12)))))));
Strict: (0 (1 ((14 (2 3))((5 (4 6))((16 (19 (20 (7 8))(17 (15 18))))((10 (9 13))(11 12)))))));

Concavity constant = 1.442
MPT's: (0 (1 ((14 (2 3))((5 (4 6))((16 ((19 (20 (7 8)))(17 (15 18))))((10 (9 13))(11 12)))))));
Strict: (0 (1 ((14 (2 3))((5 (4 6))((16 (19 (20 (7 8))(17 (15 18))))((10 (9 13))(11 12)))))));

Concavity constant = 1.522
MPT's: (0 (1 ((14 (2 3))((5 (4 6))((16 ((19 (20 (7 8)))(17 (15 18))))((10 (9 13))(11 12)))))));
Strict: (0 (1 ((14 (2 3))((5 (4 6))((16 (19 (20 (7 8))(17 (15 18))))((10 (9 13))(11 12)))))));

Concavity constant = 1.604
MPT's: (0 (1 ((14 (2 3))((5 (4 6))((16 ((19 (20 (7 8)))(17 (15 18))))((10 (9 13))(11 12)))))));
Strict: (0 (1 ((14 (2 3))((5 (4 6))((16 (19 (20 (7 8))(17 (15 18))))((10 (9 13))(11 12)))))));

Concavity constant = 1.688
MPT's: (0 (1 ((14 (2 3))((5 (4 6))((16 ((19 (20 (7 8)))(17 (15 18))))((10 (9 13))(11 12)))))));
Strict: (0 (1 ((14 (2 3))((5 (4 6))((16 (19 (20 (7 8))(17 (15 18))))((10 (9 13))(11 12)))))));

Concavity constant = 1.775
MPT's: (0 (1 ((14 (2 3))((5 (4 6))((16 ((19 (20 (7 8)))(17 (15 18))))((10 (9 13))(11 12)))))));
Strict: (0 (1 ((14 (2 3))((5 (4 6))((16 (19 (20 (7 8))(17 (15 18))))((10 (9 13))(11 12)))))));

Concavity constant = 1.864
MPT's: (0 (1 ((14 (2 3))((5 (4 6))((16 ((19 (20 (7 8)))(17 (15 18))))((10 (9 13))(11 12)))))));
Strict: (0 (1 ((14 (2 3))((5 (4 6))((16 (19 (20 (7 8))(17 (15 18))))((10 (9 13))(11 12)))))));

Concavity constant = 1.956
MPT's: (0 (1 ((14 (2 3))((5 (4 6))((16 ((19 (20 (7 8)))(17 (15 18))))((10 (9 13))(11 12)))))));
Strict: (0 (1 ((14 (2 3))((5 (4 6))((16 (19 (20 (7 8))(17 (15 18))))((10 (9 13))(11 12)))))));

Concavity constant = 2.051
MPT's: (0 (1 ((14 (2 3))((5 (4 6))((16 ((19 (20 (7 8)))(17 (15 18))))((10 (9 13))(11 12)))))));
Strict: (0 (1 ((14 (2 3))((5 (4 6))((16 (19 (20 (7 8))(17 (15 18))))((10 (9 13))(11 12)))))));

Concavity constant = 2.149
MPT's: (0 (1 ((14 (2 3))((5 (4 6))((16 ((19 (20 (7 8)))(17 (15 18))))((10 (9 13))(11 12)))))));
Strict: (0 (1 ((14 (2 3))((5 (4 6))((16 (19 (20 (7 8))(17 (15 18))))((10 (9 13))(11 12)))))));

Concavity constant = 2.251
MPT's: (0 (1 ((14 (2 3))((5 (4 6))((16 ((19 (20 (7 8)))(17 (15 18))))((10 (9 13))(11 12)))))));
Strict: (0 (1 ((14 (2 3))((5 (4 6))((16 (19 (20 (7 8))(17 (15 18))))((10 (9 13))(11 12)))))));

Concavity constant = 2.355
MPT's: (0 (1 ((14 (2 3))((5 (4 6))((16 ((19 (20 (7 8)))(17 (15 18))))((10 (9 13))(11 12)))))));
Strict: (0 (1 ((14 (2 3))((5 (4 6))((16 (19 (20 (7 8))(17 (15 18))))((10 (9 13))(11 12)))))));

Concavity constant = 2.363
MPT's: (0 (1 ((14 (2 3))((5 (4 6))((16 ((19 (20 (7 8)))(17 (15 18))))((10 (9 13))(11 12)))))));
Strict: (0 (1 ((14 (2 3))((5 (4 6))((16 (19 (20 (7 8))(17 (15 18))))((10 (9 13))(11 12)))))));

Concavity constant = 2.574
MPT's: (0 (1 ((14 (2 3))((5 (4 6))((16 ((19 (20 (7 8)))(17 (15 18))))((10 (9 13))(11 12)))))));
Strict: (0 (1 ((14 (2 3))((5 (4 6))((16 (19 (20 (7 8))(17 (15 18))))((10 (9 13))(11 12)))))));

Concavity constant = 2.689
MPT's: (0 (1 ((14 (2 3))((5 (4 6))((16 ((19 (20 (7 8)))(17 (15 18))))((10 (9 13))(11 12)))))));
Strict: (0 (1 ((14 (2 3))((5 (4 6))((16 (19 (20 (7 8))(17 (15 18))))((10 (9 13))(11 12)))))));

Concavity constant = 2.808
MPT's: (0 (1 ((14 (2 3))((5 (4 6))((16 ((19 (20 (7 8)))(17 (15 18))))((10 (9 13))(11 12)))))));
Strict: (0 (1 ((14 (2 3))((5 (4 6))((16 (19 (20 (7 8))(17 (15 18))))((10 (9 13))(11 12)))))));

Concavity constant = 2.930
MPT's: (0 (1 ((14 (2 3))((5 (4 6))((16 ((19 (20 (7 8)))(17 (15 18))))((10 (9 13))(11 12)))))));
Strict: (0 (1 ((14 (2 3))((5 (4 6))((16 (19 (20 (7 8))(17 (15 18))))((10 (9 13))(11 12)))))));

Concavity constant = 3.058
MPT's: (0 (1 ((14 (2 3))((5 (4 6))((16 ((20 (7 8))(19 (17 (15 18)))))((10 (9 13))(11 12)))))));
Strict: (0 (1 ((14 (2 3))((5 (4 6))((16 (19 (20 (7 8))(17 (15 18))))((10 (9 13))(11 12)))))));

Concavity constant = 3.189
MPT's: (0 (1 ((14 (2 3))((5 (4 6))((16 ((20 (7 8))(19 (17 (15 18)))))((10 (9 13))(11 12)))))));
Strict: (0 (1 ((14 (2 3))((5 (4 6))((16 (19 (20 (7 8))(17 (15 18))))((10 (9 13))(11 12)))))));

Concavity constant = 3.325
MPT's: (0 (1 ((14 (2 3))((5 (4 6))((16 ((20 (7 8))(19 (17 (15 18)))))((10 (9 13))(11 12)))))));
Strict: (0 (1 ((14 (2 3))((5 (4 6))((16 (19 (20 (7 8))(17 (15 18))))((10 (9 13))(11 12)))))));

Concavity constant = 3.466
MPT's: (0 (1 ((14 (2 3))((5 (4 6))((16 ((20 (7 8))(19 (17 (15 18)))))((10 (9 13))(11 12)))))));

Strict: (0 (1 ((14 (2 3))((5 (4 6))((16 (19 (20 (7 8))(17 (15 18))))((10 (9 13))(11 12)))))));

Concavity constant = 3.612
MPT's: (0 (1 ((14 (2 3))((5 (4 6))((16 ((20 (7 8))(19 (17 (15 18)))))((10 (9 13))(11 12)))))));
Strict: (0 (1 ((14 (2 3))((5 (4 6))((16 (19 (20 (7 8))(17 (15 18))))((10 (9 13))(11 12)))))));

Concavity constant = 3.764
MPT's: (0 (1 ((14 (2 3))((5 (4 6))((16 ((20 (7 8))(19 (17 (15 18)))))((10 (9 13))(11 12)))))));
Strict: (0 (1 ((14 (2 3))((5 (4 6))((16 (19 (20 (7 8))(17 (15 18))))((10 (9 13))(11 12)))))));

Concavity constant = 3.921
MPT's: (0 (1 ((14 (2 3))((5 (4 6))((16 ((20 (7 8))(19 (17 (15 18)))))((10 (9 13))(11 12)))))));
Strict: (0 (1 ((14 (2 3))((5 (4 6))((16 (19 (20 (7 8))(17 (15 18))))((10 (9 13))(11 12)))))));

Concavity constant = 4.084
MPT's: (0 (1 ((14 (2 3))((5 (4 6))((16 ((20 (7 8))(19 (17 (15 18)))))((10 (9 13))(11 12)))))));
Strict: (0 (1 ((14 (2 3))((5 (4 6))((16 (19 (20 (7 8))(17 (15 18))))((10 (9 13))(11 12)))))));

Concavity constant = 4.253
MPT's: (0 (1 ((14 (2 3))((5 (4 6))((16 ((20 (7 8))(19 (17 (15 18)))))((10 (9 13))(11 12)))))));
Strict: (0 (1 ((14 (2 3))((5 (4 6))((16 (19 (20 (7 8))(17 (15 18))))((10 (9 13))(11 12)))))));

Concavity constant = 4.429
MPT's: (0 (1 ((14 (2 3))((5 (4 6))((16 ((20 (7 8))(19 (17 (15 18)))))((10 (9 13))(11 12)))))));
Strict: (0 (1 ((14 (2 3))((5 (4 6))((16 (19 (20 (7 8))(17 (15 18))))((10 (9 13))(11 12)))))));

Concavity constant = 4.612
MPT's: (0 (1 ((14 (2 3))((5 (4 6))((16 ((20 (7 8))(19 (17 (15 18)))))((10 (9 13))(11 12)))))));
Strict: (0 (1 ((14 (2 3))((5 (4 6))((16 (19 (20 (7 8))(17 (15 18))))((10 (9 13))(11 12)))))));

Concavity constant = 4.802
MPT's: (0 (1 ((14 (2 3))((5 (4 6))((16 ((20 (7 8))(19 (17 (15 18)))))((10 (9 13))(11 12)))))));
Strict: (0 (1 ((14 (2 3))((5 (4 6))((16 (19 (20 (7 8))(17 (15 18))))((10 (9 13))(11 12)))))));

Concavity constant =5.000
MPT's: (0 (1 ((14 (2 3))((5 (4 6))((16 ((20 (7 8))(19 (17 (15 18)))))((10 (9 13))(11 12)))))));
Strict: (0 (1 ((14 (2 3))((5 (4 6))((16 (19 (20 (7 8))(17 (15 18))))((10 (9 13))(11 12)))))));

Concavity constant = 5.205
MPT's: (0 (1 ((14 (2 3))((5 (4 6))((16 ((20 (7 8))(19 (17 (15 18)))))((10 (9 13))(11 12)))))));
Strict: (0 (1 ((14 (2 3))((5 (4 6))((16 (19 (20 (7 8))(17 (15 18))))((10 (9 13))(11 12)))))));

Concavity constant =5.420
MPT's: (0 (1 ((14 (2 3))((5 (4 6))((16 ((20 (7 8))(19 (17 (15 18)))))((10 (9 13))(11 12)))))));
Strict: (0 (1 ((14 (2 3))((5 (4 6))((16 (19 (20 (7 8))(17 (15 18))))((10 (9 13))(11 12)))))));

Concavity constant =5.643
MPT's: (0 (1 ((14 (2 3))((5 (4 6))((16 ((20 (7 8))(19 (17 (15 18)))))((10 (9 13))(11 12)))))));
Strict: (0 (1 ((14 (2 3))((5 (4 6))((16 (19 (20 (7 8))(17 (15 18))))((10 (9 13))(11 12)))))));

Concavity constant = 5.877
MPT's: (0 (1 ((14 (2 3))((5 (4 6))((16 ((20 (7 8))(19 (17 (15 18)))))((10 (9 13))(11 12)))))));
Strict: (0 (1 ((14 (2 3))((5 (4 6))((16 (19 (20 (7 8))(17 (15 18))))((10 (9 13))(11 12)))))));

Concavity constant = 6.120
MPT's: (0 (1 ((14 (2 3))((5 (4 6))((16 ((20 (7 8))(19 (17 (15 18)))))((10 (9 13))(11 12)))))));
Strict: (0 (1 ((14 (2 3))((5 (4 6))((16 (19 (20 (7 8))(17 (15 18))))((10 (9 13))(11 12)))))));

Concavity constant = 6.375

MPT's: (0 (1 ((14 (2 3))((5 (4 6))((16 ((20 (7 8))(19 (17 (15 18)))))((10 (9 13))(11 12)))))));
Strict: (0 (1 ((14 (2 3))((5 (4 6))((16 (19 (20 (7 8))(17 (15 18))))((10 (9 13))(11 12)))))));

Concavity constant = 6.641
MPT's: (0 (1 ((14 (2 3))((5 (4 6))((16 ((20 (7 8))(19 (17 (15 18)))))((10 (9 13))(11 12)))))));
Strict: (0 (1 ((14 (2 3))((5 (4 6))((16 (19 (20 (7 8))(17 (15 18))))((10 (9 13))(11 12)))))));

Concavity constant = 9.919
MPT's: (0 (1 ((14 (2 3))((5 (4 6))((16 ((19 (20 (7 8)))(17 (15 18))))((10 (9 13))(11 12)))))));
Strict: (0 (1 ((14 (2 3))((5 (4 6))((16 (19 (20 (7 8))(17 (15 18))))((10 (9 13))(11 12)))))));

Concavity constant = 7.211
MPT's: (0 (1 ((14 (2 3))((5 (4 6))((16 ((20 (7 8))(19 (17 (15 18)))))((10 (9 13))(11 12)))))));
Strict: (0 (1 ((14 (2 3))((5 (4 6))((16 (19 (20 (7 8))(17 (15 18))))((10 (9 13))(11 12)))))));

Concavity constant = 7.517
MPT's: (0 (1 ((14 (2 3))((5 (4 6))((16 ((20 (7 8))(19 (17 (15 18)))))((10 (9 13))(11 12)))))));
Strict: (0 (1 ((14 (2 3))((5 (4 6))((16 (19 (20 (7 8))(17 (15 18))))((10 (9 13))(11 12)))))));

Concavity constant = 7.838
MPT's: (0 (1 ((14 (2 3))((5 (4 6))((16 ((20 (7 8))(19 (17 (15 18)))))((10 (9 13))(11 12)))))));
Strict: (0 (1 ((14 (2 3))((5 (4 6))((16 (19 (20 (7 8))(17 (15 18))))((10 (9 13))(11 12)))))));

Concavity constant = 8.175
MPT's: (0 (1 ((14 (2 3))((5 (4 6))((16 ((19 (20 (7 8)))(17 (15 18))))((10 (9 13))(11 12)))))));
Strict: (0 (1 ((14 (2 3))((5 (4 6))((16 (19 (20 (7 8))(17 (15 18))))((10 (9 13))(11 12)))))));

Concavity constant = 8.529
MPT's: (0 (1 ((14 (2 3))((5 (4 6))((16 ((20 (7 8))(19 (17 (15 18)))))((10 (9 13))(11 12)))))));
Strict: (0 (1 ((14 (2 3))((5 (4 6))((16 (19 (20 (7 8))(17 (15 18))))((10 (9 13))(11 12)))))));

Concavity constant = 8.902
MPT's: (0 (1 ((14 (2 3))((5 (4 6))((16 ((20 (7 8))(19 (17 (15 18)))))((10 (9 13))(11 12)))))));
Strict: (0 (1 ((14 (2 3))((5 (4 6))((16 (19 (20 (7 8))(17 (15 18))))((10 (9 13))(11 12)))))));

Concavity constant = 9.296
MPT's: (0 (1 ((14 (2 3))((5 (4 6))((16 ((19 (20 (7 8)))(17 (15 18))))((10 (9 13))(11 12)))))));
Strict: (0 (1 ((14 (2 3))((5 (4 6))((16 (19 (20 (7 8))(17 (15 18))))((10 (9 13))(11 12)))))));

Concavity constant = 9.711
MPT's: (0 (1 ((14 (2 3))((5 (4 6))((16 ((19 (20 (7 8)))(17 (15 18))))((10 (9 13))(11 12)))))));
Strict: (0 (1 ((14 (2 3))((5 (4 6))((16 (19 (20 (7 8))(17 (15 18))))((10 (9 13))(11 12)))))));

Concavity constant = 10.14
MPT's: (0 (1 ((14 (2 3))((5 (4 6))((16 ((19 (20 (7 8)))(17 (15 18))))((10 (9 13))(11 12)))))));
Strict: (0 (1 ((14 (2 3))((5 (4 6))((16 (19 (20 (7 8))(17 (15 18))))((10 (9 13))(11 12)))))));

Concavity constant = 10.61
MPT's: (0 (1 ((14 (2 3))((5 (4 6))((16 ((19 (20 (7 8)))(17 (15 18))))((10 (9 13))(11 12)))))));
Strict: (0 (1 ((14 (2 3))((5 (4 6))((16 (19 (20 (7 8))(17 (15 18))))((10 (9 13))(11 12)))))));

Concavity constant = 11.10
MPT's: (0 (1 ((14 (2 3))((5 (4 6))((16 ((19 (20 (7 8)))(17 (15 18))))((10 (9 13))(11 12)))))));
Strict: (0 (1 ((14 (2 3))((5 (4 6))((16 (19 (20 (7 8))(17 (15 18))))((10 (9 13))(11 12)))))));

Concavity constant = 11.62
MPT's: (0 (1 ((14 (2 3))((5 (4 6))((16 ((19 (20 (7 8)))(17 (15 18))))((10 (9 13))(11 12)))))));
Strict: (0 (1 ((14 (2 3))((5 (4 6))((16 (19 (20 (7 8))(17 (15 18))))((10 (9 13))(11 12)))))));

Concavity constant = 12.18
MPT's: (0 (1 ((14 (2 3))((5 (4 6))((16 ((19 (20 (7 8)))(17 (15 18))))((10 (9 13))(11 12)))))));
Strict: (0 (1 ((14 (2 3))((5 (4 6))((16 (19 (20 (7 8))(17 (15 18))))((10 (9 13))(11 12)))))));

Concavity constant = 12.77
MPT's: (0 (1 ((14 (2 3))((5 (4 6))((16 ((19 (20 (7 8)))(17 (15 18))))((10 (9 13))(11 12)))))));
Strict: (0 (1 ((14 (2 3))((5 (4 6))((16 (19 (20 (7 8))(17 (15 18))))((10 (9 13))(11 12)))))));

Concavity constant = 13.40
MPT's: (0 (1 ((14 (2 3))((5 (4 6))((16 ((19 (20 (7 8)))(17 (15 18))))((10 (9 13))(11 12)))))));
Strict: (0 (1 ((14 (2 3))((5 (4 6))((16 (19 (20 (7 8))(17 (15 18))))((10 (9 13))(11 12)))))));

Concavity constant = 14.08
MPT's: (0 (1 ((14 (2 3))((5 (4 6))((16 ((19 (20 (7 8)))(17 (15 18))))((10 (9 13))(11 12)))))));
Strict: (0 (1 ((14 (2 3))((5 (4 6))((16 (19 (20 (7 8))(17 (15 18))))((10 (9 13))(11 12)))))));

Concavity constant = 14.80
MPT's: (0 (1 ((14 (2 3))((5 (4 6))((16 ((19 (20 (7 8)))(17 (15 18))))((10 (9 13))(11 12)))))));
Strict: (0 (1 ((14 (2 3))((5 (4 6))((16 (19 (20 (7 8))(17 (15 18))))((10 (9 13))(11 12)))))));

Concavity constant = 15.58
MPT's: (0 (1 ((14 (2 3))((5 (4 6))((16 ((19 (20 (7 8)))(17 (15 18))))((10 (9 13))(11 12)))))));
Strict: (0 (1 ((14 (2 3))((5 (4 6))((16 (19 (20 (7 8))(17 (15 18))))((10 (9 13))(11 12)))))));

Concavity constant = 16.42
MPT's: (0 (1 ((14 (2 3))((5 (4 6))((16 ((19 (20 (7 8)))(17 (15 18))))((10 (9 13))(11 12)))))));
Strict: (0 (1 ((14 (2 3))((5 (4 6))((16 (19 (20 (7 8))(17 (15 18))))((10 (9 13))(11 12)))))));

Concavity constant = 17.32
MPT's: (0 (1 ((14 (2 3))((5 (4 6))((16 ((19 (20 (7 8)))(17 (15 18))))((10 (9 13))(11 12)))))));
Strict: (0 (1 ((14 (2 3))((5 (4 6))((16 (19 (20 (7 8))(17 (15 18))))((10 (9 13))(11 12)))))));

Concavity constant = 18.30
MPT's: (0 (1 ((14 (2 3))((5 (4 6))((16 ((19 (20 (7 8)))(17 (15 18))))((10 (9 13))(11 12)))))));
Strict: (0 (1 ((14 (2 3))((5 (4 6))((16 (19 (20 (7 8))(17 (15 18))))((10 (9 13))(11 12)))))));

Concavity constant = 19.37
MPT's: (0 (1 ((14 (2 3))((5 (4 6))((16 ((19 (20 (7 8)))(17 (15 18))))((10 (9 13))(11 12)))))));
Strict: (0 (1 ((14 (2 3))((5 (4 6))((16 (19 (20 (7 8))(17 (15 18))))((10 (9 13))(11 12)))))));

Concavity constant = 20.53
MPT's: (0 (1 ((14 (2 3))((5 (4 6))((16 ((19 (20 (7 8)))(17 (15 18))))((10 (9 13))(11 12)))))));
Strict: (0 (1 ((14 (2 3))((5 (4 6))((16 (19 (20 (7 8))(17 (15 18))))((10 (9 13))(11 12)))))));

Concavity constant = 21.81
MPT's: (0 (1 ((14 (2 3))((5 (4 6))((16 ((19 (20 (7 8)))(17 (15 18))))((10 (9 13))(11 12)))))));
Strict: (0 (1 ((14 (2 3))((5 (4 6))((16 (19 (20 (7 8))(17 (15 18))))((10 (9 13))(11 12)))))));

Concavity constant = 23.22
MPT's: (0 (1 ((14 (2 3))((5 (4 6))((16 ((19 (20 (7 8)))(17 (15 18))))((10 (9 13))(11 12)))))));
Strict: (0 (1 ((14 (2 3))((5 (4 6))((16 (19 (20 (7 8))(17 (15 18))))((10 (9 13))(11 12)))))));

Concavity constant = 24.77
MPT's: (0 (1 ((14 (2 3))((5 (4 6))((16 ((19 (20 (7 8)))(17 (15 18))))((10 (9 13))(11 12)))))));
Strict: (0 (1 ((14 (2 3))((5 (4 6))((16 (19 (20 (7 8))(17 (15 18))))((10 (9 13))(11 12)))))));

Concavity constant = 26.50
MPT's: (0 (1 ((14 (2 3))((5 (4 6))((16 ((19 (20 (7 8)))(17 (15 18))))((10 (9 13))(11 12)))))));
Strict: (0 (1 ((14 (2 3))((5 (4 6))((16 (19 (20 (7 8))(17 (15 18))))((10 (9 13))(11 12)))))));

Concavity constant = 28.44
MPT's: (0 (1 ((14 (2 3))((5 (4 6))((16 ((19 (20 (7 8)))(17 (15 18))))((10 (9 13))(11 12)))))));
Strict: (0 (1 ((14 (2 3))((5 (4 6))((16 (19 (20 (7 8))(17 (15 18))))((10 (9 13))(11 12)))))));

Concavity constant = 30.63
MPT's: (0 (1 ((14 (2 3))((5 (4 6))((16 ((19 (20 (7 8)))(17 (15 18))))((10 (9 13))(11 12)))))));
Strict: (0 (1 ((14 (2 3))((5 (4 6))((16 (19 (20 (7 8))(17 (15 18))))((10 (9 13))(11 12)))))));

Concavity constant = 33.13
MPT's: (0 (1 ((14 (2 3))((5 (4 6))((16 ((19 (20 (7 8)))(17 (15 18))))((10 (9 13))(11 12)))))));
Strict: (0 (1 ((14 (2 3))((5 (4 6))((16 (19 (20 (7 8))(17 (15 18))))((10 (9 13))(11 12)))))));

Concavity constant = 35.99
MPT's: (0 (1 ((14 (2 3))((5 (4 6))((16 ((19 (20 (7 8)))(17 (15 18))))((10 (9 13))(11 12)))))));
Strict: (0 (1 ((14 (2 3))((5 (4 6))((16 (19 (20 (7 8))(17 (15 18))))((10 (9 13))(11 12)))))));

Concavity constant = 39.33
MPT's: (0 (1 ((14 (2 3))((5 (4 6))((16 ((19 (20 (7 8)))(17 (15 18))))((10 (9 13))(11 12)))))));
Strict: (0 (1 ((14 (2 3))((5 (4 6))((16 (19 (20 (7 8))(17 (15 18))))((10 (9 13))(11 12)))))));

Concavity constant = 43.26
MPT's: (0 (1 ((14 (2 3))((5 (4 6))((16 ((19 (20 (7 8)))(17 (15 18))))((10 (9 13))(11 12)))))));
Strict: (0 (1 ((14 (2 3))((5 (4 6))((16 (19 (20 (7 8))(17 (15 18))))((10 (9 13))(11 12)))))));

Concavity constant = 47.98
MPT's: (0 (1 ((14 (2 3))((5 (4 6))((16 ((19 (20 (7 8)))(17 (15 18))))((10 (9 13))(11 12)))))));
Strict: (0 (1 ((14 (2 3))((5 (4 6))((16 (19 (20 (7 8))(17 (15 18))))((10 (9 13))(11 12)))))));

Concavity constant = 53.76
MPT's: (0 (1 ((14 (2 3))((5 (4 6))((16 ((19 (20 (7 8)))(17 (15 18))))((10 (9 13))(11 12)))))));
Strict: (0 (1 ((14 (2 3))((5 (4 6))((16 (19 (20 (7 8))(17 (15 18))))((10 (9 13))(11 12)))))));

Concavity constant = 61.05
MPT's: (0 (1 ((14 (2 3))((5 (4 6))((16 ((19 (20 (7 8)))(17 (15 18))))((10 (9 13))(11 12)))))));
Strict: (0 (1 ((14 (2 3))((5 (4 6))((16 (19 (20 (7 8))(17 (15 18))))((10 (9 13))(11 12)))))));

Concavity constant = 70.57
MPT's: (0 (1 ((14 (2 3))((5 (4 6))((16 ((19 (20 (7 8)))(17 (15 18))))((10 (9 13))(11 12)))))));
Strict: (0 (1 ((14 (2 3))((5 (4 6))((16 (19 (20 (7 8))(17 (15 18))))((10 (9 13))(11 12)))))));

Concavity constant = 83.68
MPT's: (0 (1 ((14 (2 3))((5 (4 6))((16 ((19 (20 (7 8)))(17 (15 18))))((10 (9 13))(11 12)))))));
Strict: (0 (1 ((14 (2 3))((5 (4 6))((16 (19 (20 (7 8))(17 (15 18))))((10 (9 13))(11 12)))))));

Concavity constant = 103.1
MPT's: (0 (1 ((14 (2 3))((5 (4 6))((16 ((19 (20 (7 8)))(17 (15 18))))((10 (9 13))(11 12)))))));
Strict: (0 (1 ((14 (2 3))((5 (4 6))((16 (19 (20 (7 8))(17 (15 18))))((10 (9 13))(11 12)))))));

Concavity constant = 136.2
MPT's: (0 (1 ((14 (2 3))((5 (4 6))((16 ((19 (20 (7 8)))(17 (15 18))))((10 (9 13))(11 12)))))));
Strict: (0 (1 ((14 (2 3))((5 (4 6))((16 (19 (20 (7 8))(17 (15 18))))((10 (9 13))(11 12)))))));

Concavity constant = 211.3
MPT's: (0 (1 ((14 (2 3))((5 (4 6))((16 ((20 (7 8))(19 (17 (15 18)))))((10 (9 13))(11 12)))))));
Strict: (0 (1 ((14 (2 3))((5 (4 6))((16 (19 (20 (7 8))(17 (15 18))))((10 (9 13))(11 12)))))));

Concavity constant = Equal
MPT's: (0 (1 ((14 (2 3))((5 (4 6))((16 (19 ((20 (7 8))(17 (15 18)))))((10 (9 13))(11 12)))))))*

(0 (1 ((14 (2 3))((5 (4 6))(((20 (7 8))((10 (9 13))(11 12)))(16 (19 (17 (15 18)))))))));

Strict: (0 (1 ((14 (2 3))((5 (4 6))(16 19 (20 (7 8))((10 (9 13))(11 12))(17 (15 18)))))));

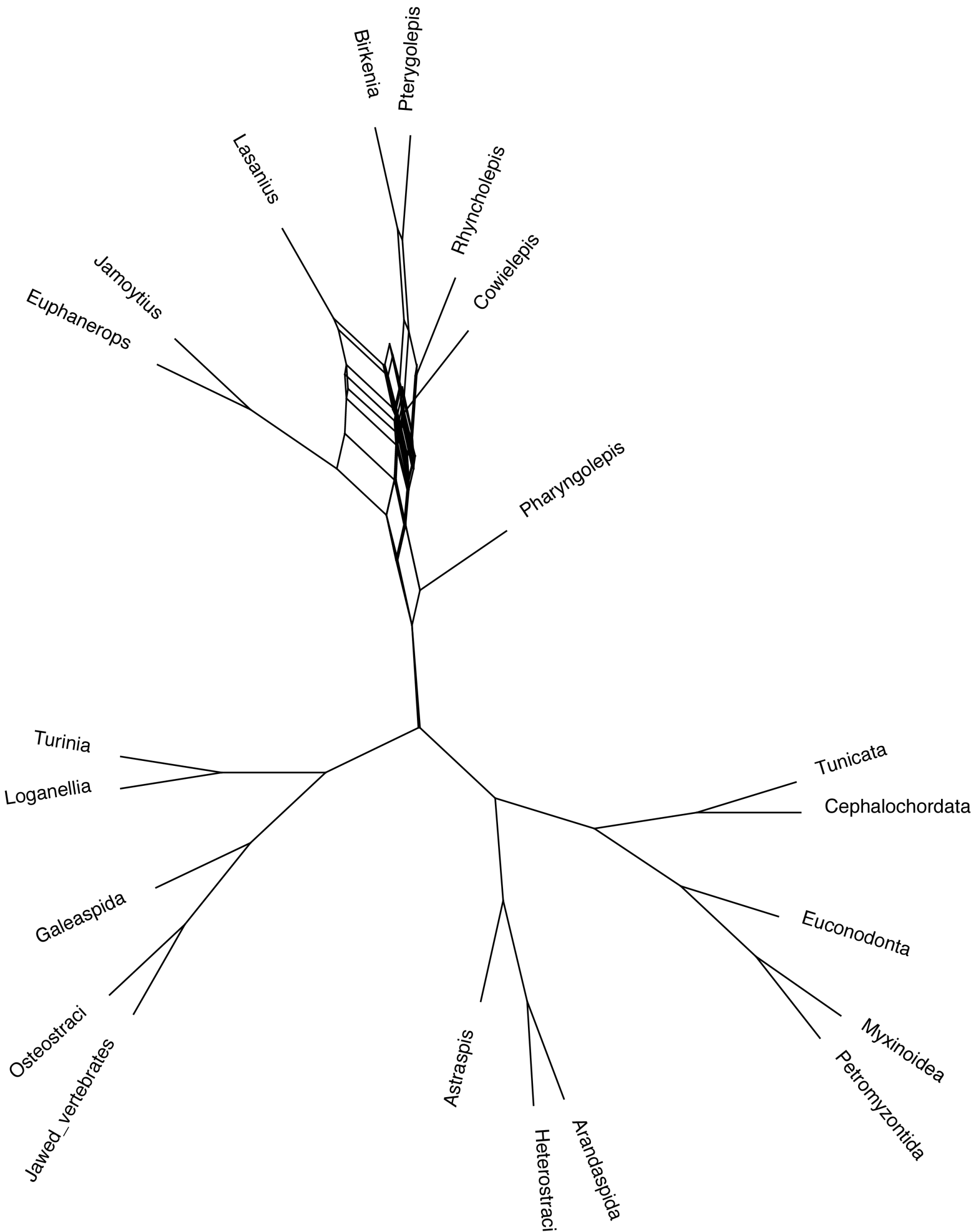

Fig. 1 Splits consensus network of all MPT's derived from 100 analyses at 100 different concavity constants (including k = ∞). Edge lengths are proportional to the number of MPT's in which the branch is recovered.

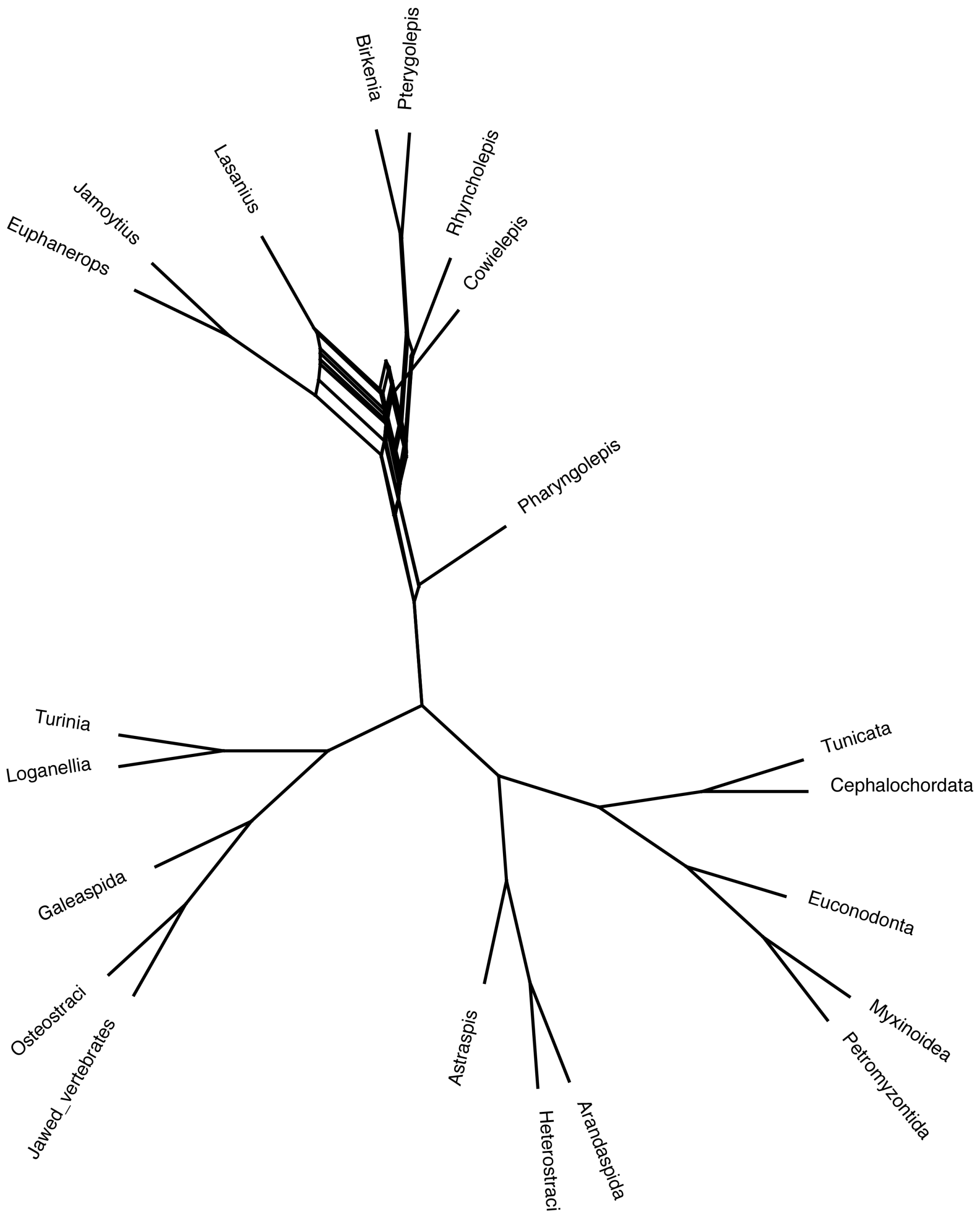

Fig. 2. Normalized splits consensus network of all MPT's derived from 100 analyses at 100 different concavity constants (including k = ∞). Edge length equals the sum of the proportion of MPT's with edge (*e*) at each value of k.

References

Blom H. 2012. New birkeniid anaspid from the Lower Devonian of Scotland and its phylogenetic implications. Palaeontology 55(3):641-652.

Brazeau MD. 2011. Problematic character coding methods in morphology and their effects. Biological Journal of the Linnean Society 104(3):489-498.

Conway Morris S, Caron J-B. 2014. A primitive fish from the Cambrian of North America. Nature 512(7515):419-422.

Donoghue PCJ, Forey PL, Aldridge RJ. 2000. Conodont affinity and chordate phylogeny. Biological Reviews of the Cambridge Philosophical Society 75(2):191-251.

Gess RW, Coates MI, Rubidge BS. 2006. A lamprey from the Devonian period of South Africa. Nature 443(7114):981-984.

Goloboff PA. 1993. Estimating character weights during tree search. Cladistics 9(1):83-91.

Huson DH, Bryant D. 2006. Application of phylogenetic networks in evolutionary studies. Molecular biology and evolution 23(2):254-267.

Sansom RS, Freedman K, Gabbott SE, Aldridge RJ, Purnell MA. 2010. Taphonomy and affinity of an enigmatic Silurian vertebrate, Jamoytius kerwoodi White. Palaeontology 53(6):1393-1409.

Smith MR, Ortega-Hernández J. 2014. Hallucigenia/'s onychophoran-like claws and the case for Tactopoda. Nature 514(7522):363-366.
